# Synthesis of aromatic glycoconjugates. Building blocks for the construction of combinatorial glycopeptide libraries

**DOI:** 10.3762/bjoc.10.256

**Published:** 2014-10-22

**Authors:** Markus Nörrlinger, Thomas Ziegler

**Affiliations:** 1 Institute of Organic Chemistry, University of Tuebingen, Auf der Morgenstelle 18, 72076 Tuebingen, Germany

**Keywords:** amino acids, aniline, carbohydrates, glycoconjugates, glycopeptides

## Abstract

New aromatic glycoconjugate building blocks based on the trifunctional 3-aminomethyl-5-aminobenzoic acid backbone and sugars linked to the backbone by a malonyl moiety were prepared via peptide coupling. The orthogonally protected glycoconjugates, bearing an acetyl-protected glycoside, were converted into their corresponding acids which are suitable building blocks for combinatorial glycopeptide synthesis.

## Introduction

**Figure 1:**
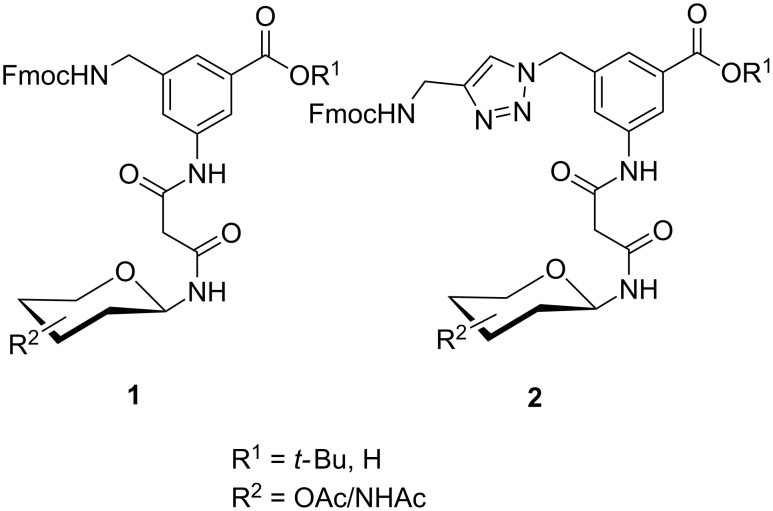
Malonyl-linked aromatic glycoconjugate building blocks for spot synthesis of combinatorial glycopeptides libraries.

Glycans or other complex oligosaccharide structures, present on the surface of every prokaryotic and eukaryotic cell, are important for a large number of biological recognition processes like, for example, intercellular communication, signal transduction, pathogen recognition or immunological responses [1–4]. In order to investigate these processes it is essential that a large amount of the respective polysaccharide structure is available. Unfortunately, isolation of pure oligosaccharides from natural sources is difficult due to the micro heterogenity of naturally occurring saccharides. For this reason chemical oligosaccharide synthesis is the only alternative for providing sufficient amounts of pure material for detailed biological studies. However, the synthetic preparation of complex oligosaccharides is still difficult despite the great achievements in this field during the past decades. Therefore, the application of oligosaccharide mimetics which may be synthesized more easily in larger amounts appears to be a useful tool to investigate, for instance, specific carbohydrate–protein or carbohydrate–carbohydrate interactions. Recently our group has prepared a series of trifunctional glycopeptide building blocks with aliphatic backbones, which allow for the automated construction of combinatorial libraries of highly divers glycopeptides suitable for studying carbohydrate–protein interactions [5–8]. Hitherto, our focus was on glycosylated amino acid building blocks derived from aspartic acid and from the PNA-like *N*-(2-aminoethyl)glycine (AEG) backbone to which the sugar moieties were attached through either simple alkyl chains [5–6], amino alcohols [7–8] or 1,2,3-triazoles [9–11]. These building blocks were used for combinatorial solid phase or automated spot synthesis of libraries of highly glycosylated peptides and shown to specifically bind to lectins [5,8,10]. Here, we now describe the preparation of a series of glycopeptide building blocks which allow for the construction of glycopeptide libraries with an aromatic backbone based on 3-aminomethyl-5-aminobenzoic acid to which the sugar moieties are attached through a malonyl linker. For that purpose we designed the two building blocks **1** and **2** (Figure 1) which both can be converted into the respective glycopeptides using standard Fmoc strategies [10].

## Results and Discussion

**Scheme 1:**
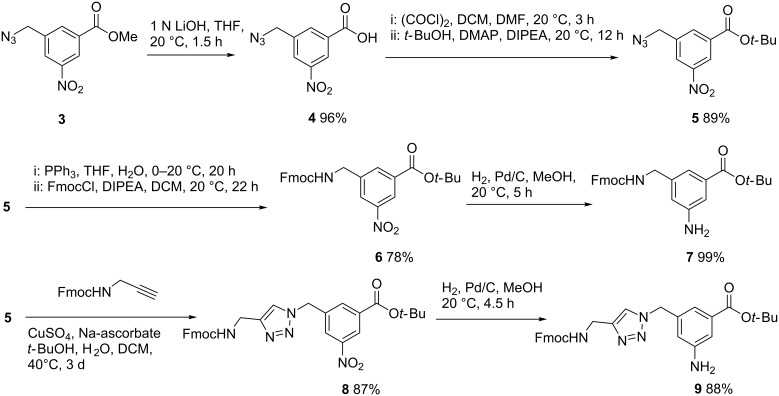
Synthesis of the aromatic backbone building blocks **7** and **9**.

Our synthesis of building blocks **1** and **2** started from known 3-azidomethyl-5-nitrobenzoic acid methyl ester **3** which was prepared from commercially available dimethyl 5-nitroisophthalate in 64% overall yield [12–13]. Saponification of the methyl ester in **3** with aqueous LiOH solution in THF afforded the corresponding carboxylic acid **4** in 96% yield. Next, acid **4** was converted into *tert*-butyl ester **5** in 89% overall yield by a two-step procedure via the corresponding intermediate acid chloride (Scheme 1). Selective reduction of the azido group in **5** without affecting the nitro group was achieved with a Staudinger reaction [14]. Thus, treatment of **5** with triphenylphosphine in aqueous THF gave *tert*-butyl 3-aminomethyl-5-nitrobenzoate which was not isolated but immediately converted into the corresponding Fmoc-protected derivative **6** in 78% overall yield. Final hydrogenation of the latter with Lindlar catalyst afforded **7** almost quantitatively. Likewise, copper(I)-catalyzed 1,3-dipolar cycloaddition (Click reaction) [15–18] of **5** with Fmoc-protected propargylamine afforded first *t*-butyl benzoate **8** in 87% yield. Hydrogenation of the latter with Pd on charcoal then gave **9** in 88% yield. It should be noted that hydrogenation of **6** and **8** had to be carefully optimized with respect to the reaction conditions in order to completely suppress the hydrogenation of the Fmoc group (Scheme 1).

For the construction of the two desired glycopetide building blocks **1** and **2** we needed a series of 1-malonylamidoglycopyranoses **12** for condensation with the aromatic backbone building blocks **7** and **9**. For that purpose we chose four sugar ligands in the *gluco*, *galacto*, *N-*acetylglucosamine and galactosamine series which were prepared as outlined in Table 1. Glycosylamines **10a–d** were prepared from the corresponding glycosylazides by hydrogenation according to known procedures [19–23]. Next, glycosylamines **10** were condensed with *tert*-butyl malonate [24], *N,N,N′,N′*-tetramethyl-*O-*(1*H*-benzotriazol-1-yl)uronium hexafluorophosphate (HBTU) and triethylamine in THF to give monosaccharides **11** in 53–76% yield. The medium yields in case of **11b–d** (Table 1, entries 2–4) were due to some decomposition during chromatographic purification. Nevertheless, in our hands, HBTU was superior to other peptide coupling reagents because it resulted in the highest yields of compounds **11**. HBTU was also previously used for the preparation of a similar *t*-butyl succinate of glucosamine [25]. Upon treatment with trifluoroacetic acid in dichloromethane, esters **11** were converted into the corresponding free acids **12** in 91–98% yield (Table 1).

**Table 1:** Synthesis of glycosides **11** and **12**.

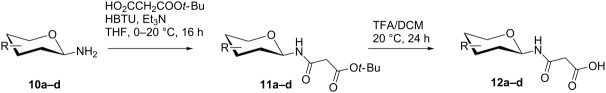				

Entry	Starting Material [ref]	Products	Yield **11** (%)	Yield **12** (%)

1	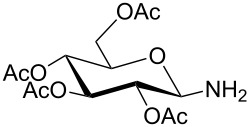 **10a** [19–20]	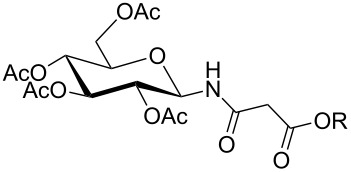 R = *t*-Bu **11a** R = H **12a**	**11a** 76	**12a** 91
2	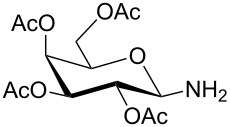 **10b** [20]	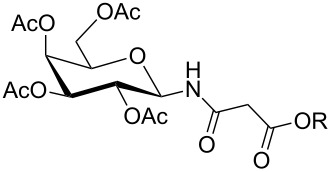 R = *t*-Bu **11b** R = H **12b**	**11b** 56	**12b** 96
3	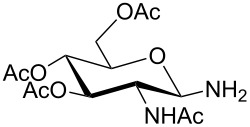 **10c** [21]	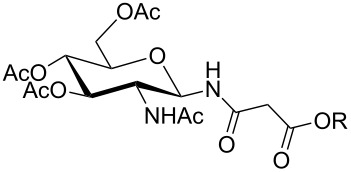 R = *t*-Bu **11c** R = H **12c**	**11c** 56	**12c** 98
4	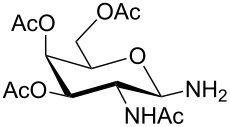 **10d** [22–23]	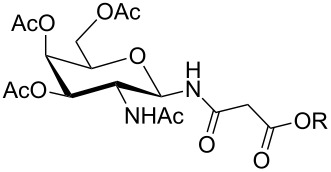 R = *t*-Bu **11d** R = H **12d**	**11d** 53	**12d** 98

Finally, glycopeptide building blocks **1** and **2** were prepared as follows (Table 2). Both Fmoc-protected benzoic acid derivatives **7** and **9** (Scheme 1) were each condensed with each of the four malonylamidoglycosides **12a–d** (Table 1) to afford eight intermediate *tert*-butyl esters **13** and **14** in 45–67% yield. For the condensation step between **7** and **9** with **12**, respectively, we chose 1-ethyl-3-(3-dimethylaminopropyl)carbodiimide (EDCI) instead of HBTU as the coupling reagent because the side product (1-(3-(dimethylamino)propyl)-3-ethylurea) released from EDCI was easily removed from the crude reaction mixture by washing with aqueous citric acid. The medium yields for these condensation steps were due to some difficulties to completely separate the products from concomitant minor unidentified byproducts which showed similar mobilities during chromatographic purification. Next, the *tert*-butyl ester groups of compounds **13** and **14** were hydrolysed with a 2:1 mixture of formic acid and dichloromethane at room temperature to give the corresponding free acids, i.e., building blocks **1a–d** and **2a–d** in 68–95% yield (Table 2).

**Table 2:** Synthesis of glycopeptide building blocks **1** and **2**.

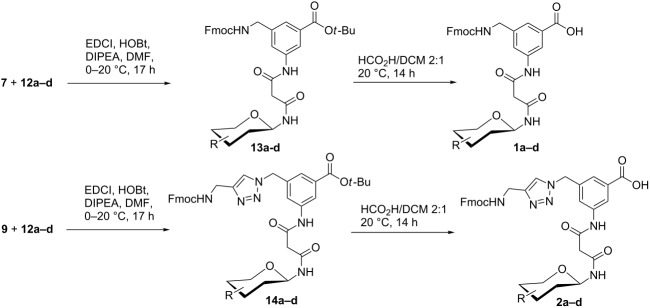				

Entry	Products **13**, **14**	Yield (%)	Products **1**, **2**	Yield (%)

1	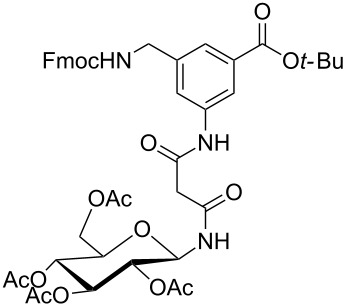 **13a**	54	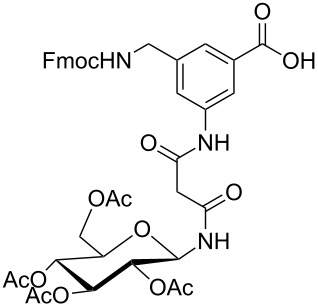 **1a**	95
2	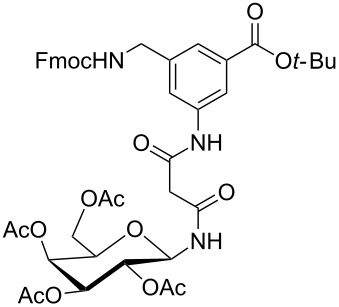 **13b**	57	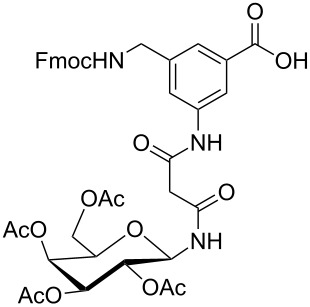 **1b**	90
3	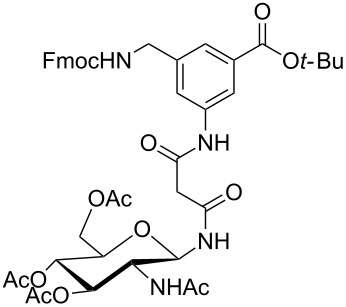 **13c**	48	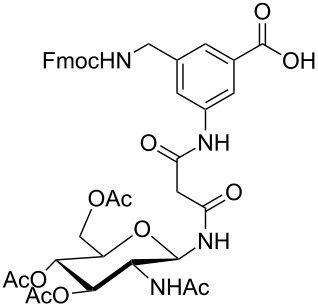 **1c**	68
4	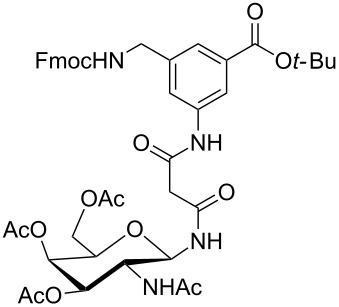 **13d**	61	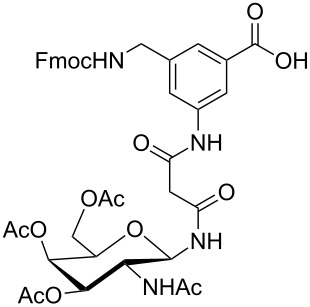 **1d**	94
5	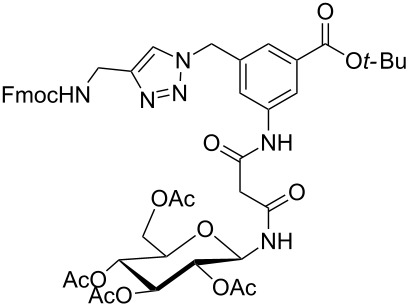 **14a**	62	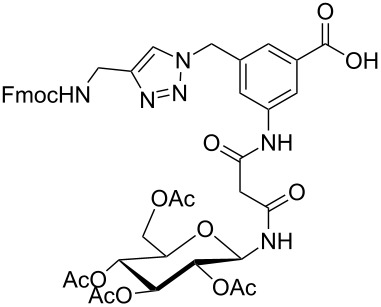 **2a**	92
6	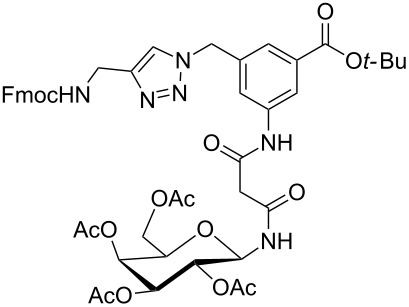 **14b**	45	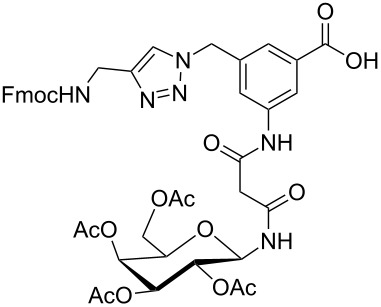 **2b**	84
7	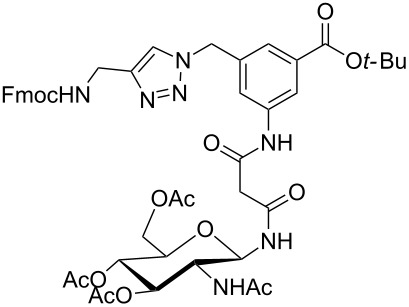 **14c**	67	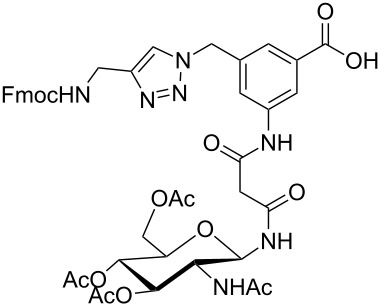 **2c**	91
8	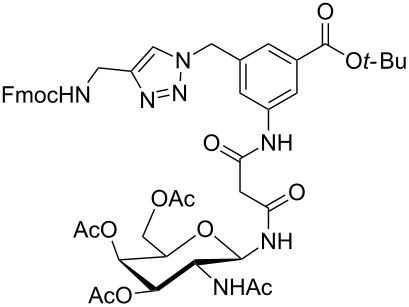 **14d**	65	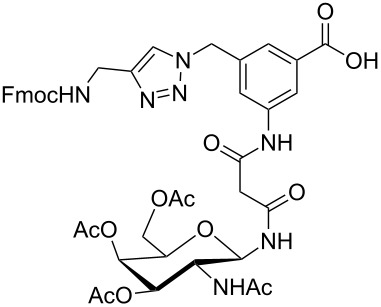 **2d**	94

**Scheme 2:**
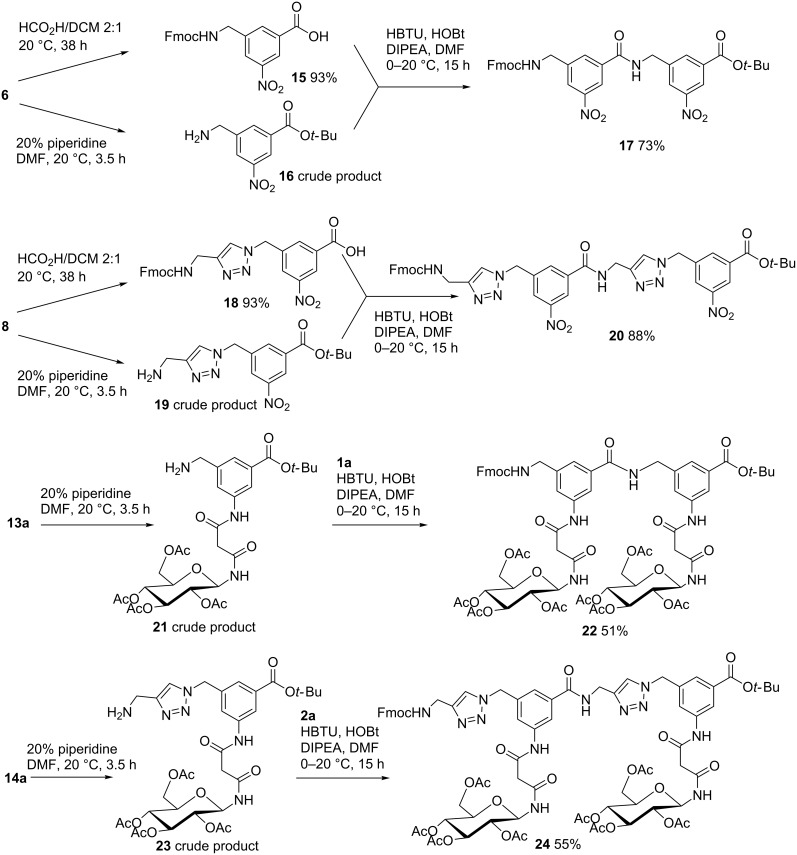
Synthesis of dimers **17**, **20**, **22** and **24**.

In order to demonstrate that building blocks **1** and **2** are indeed suitable for the construction of combinatorial glycopeptide libraries we chose glucose-containing derivatives **1a** and **2a** for an exemplified preparation of the corresponding fully protected dimers. For comparison reasons and the possibility to later construct glycopeptides containing non-glycosylated chain links, we also prepared two dipeptides from nitro-benzoates **6** and **8** in the following way (Scheme 2). Treatment of **6** and **8** with a 2:1 mixture of formic acid and dichloromethane at room temperature for 38 h, as described for the preparation of building blocks **1** and **2** (see Table 2), afforded the benzoic acid derivatives **15** and **18** in 93% yield for each. Likewise, treatment of **5** and **8** with 20% piperidine in DMF at room temperature for 3.5 h gave crude aminomethyl compounds **16** and **19** which were used for the next step without further purification. Final coupling of **15** with **16** and **18** with **19** using HBTU, 1-hydroxybenzotriazole (HOBt) and diisopropylethylamine (DIPEA) in DMF as the condenation agent gave non-glycosylated fully protected dipeptides **17** and **20** in 73% and 88% yield, respectively (Scheme 2). Likewise, the Fmoc protecting groups in glucosylated building blocks **13a** and **14a** were first removed with piperidine in DMF to give crude aminomethyl derivates **21** and **23**. Next, the latter were coupled with **1a** (for **21**) and **2a** (for **23**) under similar conditions as described for the non-glycosylated counterparts above to afford glycol-dipeptides **22** and **24** in 51% and 55% yield, respectively. As was observed for EDCI-promoted preparation of glycosylated derivatives **13** and **14** (see Table 2), the yields of the two gluco-dipeptides were only in the medium range due to traces of unidentified byproducts which could not easily removed during chromatographic purification. Nevertheless, the only medium yields in this case can be circumvented in solid phase syntheses of such glycopeptides where an excess of one building block can be applied and no chromatographic purification is necessary. Such combinatorial solid phase syntheses with the building blocks described here are now underway.

## Conclusion

We have described the preparation of a series of new aromatic glycopeptoids and have demonstrated their usefulness for the preparation of corresponding glycosylated or non-glycosylated dipeptides. The benzoic acid derived building blocks described here will be used in combination with previously described similar glycopeptoids based on asparaginic acid or PNA-like backbones for automated SPOT syntheses of combinatorial glycopeptide libraries which will be screened for their capability to bind to specific proteins [5–11]. Those results will be published elsewhere.

## Supporting Information

File 1 Experimental data.

File 2 NMR Spectra.

## References

[1] Benett H S (1963). J Histochem Cytochem.

[2] Lee Y C, Lee R T (1995). Acc Chem Res.

[3] Dwek R A (1996). Chem Rev.

[4] Solis D, Romero A, Menendez M, Jimenez-Barbero J, Gabius H-J (2009). The Sugar Code.

[5] Ziegler T, Röseling D, Subramanian L R (2002). Tetrahedron: Asymmetry.

[6] Daiber R, Ziegler T (2013). ARKIVOC.

[7] Schips C, Ziegler T (2005). J Carbohydr Chem.

[8] Ziegler T, Schips C (2006). Nat Protoc.

[9] Pietrzik N, Schips C, Ziegler T (2008). Synthesis.

[10] Günther K, Schips C, Ziegler T (2008). J Carbohydr Chem.

[11] Günther K U, Ziegler T (2014). Synthesis.

[12] Harris T D, Rajopadhye M, Damphousse P J, Glowacka D, Yu K, Bourque J P, Barrett J A, Damphousse D J, Heminway S J, Lazewatsky J (1996). Bioorg Med Chem Lett.

[13] Watzke A, Gutierrez-Rodriguez M, Köhn M, Wacker R, Schroeder H, Breinbauer R, Kuhlmann J, Alexandrov K, Niemeyer C M, Goody R S (2006). Bioorg Med Chem.

[14] Staudinger H, Meyer J (1919). Helv Chim Acta.

[15] Rostovtsev V V, Green L, Fokin V V, Sharpless K B (2002). Angew Chem, Int Ed.

[16] Tornoe C W, Christensen C, Meldal M (2002). J Org Chem.

[17] Bock V D, Hiemstra H, van Maarseveen J H (2006). Eur J Org Chem.

[18] Dedola S, Nepogodiev S A, Field R A (2007). Org Biomol Chem.

[19] Helferich B, Mitrowsky A (1952). Chem Ber.

[20] Badia C, Souard F, Vicent C (2012). J Org Chem.

[21] Neumann J, Thiem J (2010). Eur J Org Chem.

[22] Dunstan D, Hough L (1972). Carbohydr Res.

[23] Tanaka M, Yamashina I (1973). Carbohydr Res.

[24] Tararov V I, Korosteylev A, König G, Börner A (2006). Synth Commun.

[25] Fujita Y, Abdel-Aal A-B M, Wimmer N, Batzloff M R, Good M F, Toth I (2008). Bioorg Med Chem.

