# Supplementary material for: Synthesis of aromatic glycoconjugates. Building blocks for the construction of combinatorial glycopeptide libraries
Source: Beilstein J Org Chem. 2014 Oct 22;10:2453–60. doi: 10.3762/bjoc.10.256 (PMC4222372; doi:10.3762/bjoc.10.256)
Supplement: File 1 — Experimental data. [file Beilstein_J_Org_Chem-10-2453-s001.pdf]

Supporting Information

for

Synthesis of aromatic glycoconjugates. Building blocks for the construction of combinatorial glycopeptide libraries

Markus Nörrlinger, Thomas Ziegler*

Address: Institute of Organic Chemistry, University of Tuebingen, Auf der Morgenstelle 18, 72076 Tuebingen, Germany

Email: Thomas Ziegler - thomas.ziegler@uni-tuebingen.de

* Corresponding author

Experimental data

General

All solvents were dried and distilled prior to their use. Reactions were performed under Ar and monitored by TLC on Polygram Sil G/UV silica gel plates from Machery & Nagel. Detection was effected by charring with H_2SO_4 (5% in EtOH) or by inspection of the TLC plates under UV light. NMR spectra were recorded on a Bruker ARX 400 spectrometer at 400 MHz for proton spectra and 100 MHz for carbon spectra. Tetramethylsilane was used at the internal standard. Chemical shifts δ are given in ppm and coupling constants in Hz. All NMR spectra were treated as first-order spectra. HRMS was performed on a Bruker Daltonics APEX 2 FT–ICR spectrometer. FAB MS was performed on a Finnigan MAT TSQ 70 spectrometer and

ionization with Xe. Elemental analyses were performed on a Hekatech Euro 3000 CHN analyzer. Optical rotations were measured with a Perkin-Elmer Polarimeter 341. Melting points were determined with a Büchi B-540 apparatus and are uncorrected. Preparative chromatography was performed on silica gel (0.032–0.063 mm) from Machery & Nagel using different mixtures of solvents as eluent.

Starting materials

The following aromate **3**, the Fmoc-propargylamine and the glycosylamines **10a-d** were prepared to literature procedures. 3-Azidomethyl-5-nitrobenzoic acid methyl ester **3** [1-2], Fmoc-propargylamine [3] [2,3,4,6-Tetra-*O*-acetyl-β-D-glucopyranosylamine **10a** [4-6], 2,3,4,6-Tetra-*O*-acetyl-β-D-galactopyranosylamine **10b** [5-6], 2-Acetamido-2-deoxy-3,4,6-Tri-*O*-acetyl-β-D-glucopyranosylamine **10c** [6-7], 2-Acetamido-2-deoxy-3,4,6-Tri-*O*-acetyl-β-D-galactopyranosylamine **10d** [6,8].

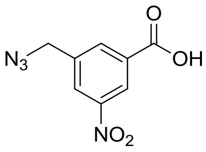

4

3-Azidomethyl-5-nitrobenzoic acid (4)

A solution of 1.53 g **3** [1-2] (6.48 mmol; 1 eq) in 20 ml dry THF and 13 ml 1 N LiOH-soln. (12.96 mmol; 2 eq) was stirred at r.t. for 1 ½ h. Afterwards the THF was evaporated, the residue was dissolved in 100 ml DCM and acidified with 75 ml 1 M HCl-soln. The organic layer was dried over Na_2SO_4 and the solvent was removed under reduced pressure yielding 1.38 g of pure title compound **4** (6.21 mmol; 96 %)

as white solid. ^{1}H-NMR (DMSO-d_6) δ 13.79 (s, 1H, CO_2H), 8.56 (s, 1H, H-aryl), 8.47 (s, 1H, H-aryl), 8.33 (s, 1H, H-aryl), 4.76 (s, 2H, N_3CH_2); ^{13}C-NMR (DMSO-d_6) δ 165.3 (1C, C=O), 148.1, 139.1, 134.8, 132.9, 126.7, 123.2 (6C, C-aryl), 52.01 (N_3CH_2); FAB-MS *m/z* 220.8 $[M-H]^-$; IR (KBr): 3442 cm^{-1}, 3091 cm^{-1}, 2114 cm^{-1}, 1696 cm^{-1}, 1619 cm^{-1}, 1590 cm^{-1}, 1536 cm^{-1}; Anal. Calcd for $C_8H_6N_4O_4$ (222.16): C 43.25; H 2.72; N 25.22; found: C 43.37; H 2.75; N 25.15.

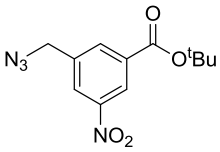

5

3-Azidomethyl-5-nitrobenzoic acid *tert*-butyl ester (5)

In a 250 ml round bottom flask equipped with a gas inlet and a stirring bar 4.43 g **4** (19.94 mmol; 1 eq) was suspended in 100 ml dry DCM under an atmosphere of nitrogen. To the suspension were added dropwise 5.13 ml $(COCl)_2$ (59.82 mmol; 3 eq) and 0.4 ml dry DMF. The solution was stirred at r.t. for 3 h. Afterwards the solvent was evaporated under reduced pressure and the residue was dissolved in 38 ml ^{t}BuOH (399.00 mmol; 20 eq). Thereafter 2.44 g DMAP (19.94 mmol; 1 eq) and 34 ml DIPEA (199.40 mmol; 10 eq) were added and the mixture was stirred at r.t for 12 h. The solvent was evaporated and the residue was dissolved in 400 ml DCM. The organic layer was washed with 1 M NaOH-soln. (3 x 100 ml) and 1 M HCl-soln. (2 x 100 ml), dried over Na_2SO_4 and the solvent was removed under reduced pressure. Purification of the residue via column chromatography eluting with PE/EA 8:1 afforded 4.96 g of pure title compound **5** (17.83 mmol; 89 %) as pale yellow solid. R_f: 0.31 (PE/EA 8:1); Mp: 35.3 °C (EtOH); ^{1}H-NMR ($CDCl_3$) δ 8.73 (s, 1H, H-aryl), 8.35

(s, 1H, H-aryl), 8.25 (s, 1H, H-aryl), 4.56 (s, 2H, N_3CH_2), 1.63 (s, 9H, $CO_2C(CH_3)_3$); ^{13}C-NMR ($CDCl_3$) δ 163.2 (1C, C=O), 148.7, 138.2, 134.5, 134.4, 126.2, 124.1 (6C, C-aryl), 83.1 (1C, $CO_2C(CH_3)$), 53.6 (1C, N_3CH_2), 28.2 (3C, $CO_2C(CH_3)_3$); FAB-MS: *m/z* 279.1 $[M+H]^+$; IR (KBr): 3091 cm^{-1}, 2984 cm^{-1}, 2109 cm^{-1}, 1717 cm^{-1}, 1623 cm^{-1}, 1590 cm^{-1}, 1540 cm^{-1}; Anal. Calcd for $C_{12}H_{14}N_4O_4$(278.26): C 51.80; H 5.07; N 20.13; found: C 52.13; H 5.09; N 20.19.

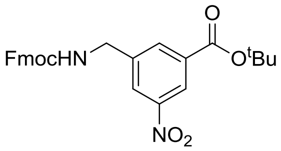

6

3-[(9*H*-Fluoren-9-ylmethoxycarbonyl)aminomethyl]-5-nitrobenzoic acid *tert*-butyl ester (6)

In a 100 ml round bottom flask equipped with a gas inlet and a stirring bar 2.00 g **5** (7.19 mmol; 1 eq) was dissolved in 30 ml dry THF under an atmosphere of nitrogen. To the solution was cooled to 0°C and 2.07 g PPh_3 (7.91 mmol; 1.1 eq) was added. The mixture was stirred at r.t. for 15 h. Afterwards 5 ml H_2O was added and the solution was stirred at r.t. for 5 h. The solvent was evaporated and the residue was dissolved in 40 ml dry DCM and 4.37 ml DIPEA (33.78 mmol; 4.7 eq) and 2.79 g FmocCl (10.78 mmol; 1.5 eq) were added. The solution was stirred at r.t. for 22 h. Thereafter the solution was diluted with 300 ml DCM and transferred to a separatory funnel. The organic layer was washed with 1 M HCl-soln. (1 x 100 ml), sat. $NaHCO_3$-soln. (1 x 100 ml) and sat. NaCl-soln. (1 x 50 ml), dried over Na_2SO_4 and the solvent was removed under reduced pressure. Purification of the residue via column chromatography eluting with PE/EE 4:1→1:1 and recrystallization from *n*-hexane/$CHCl_3$ afforded 2.66 g of pure title compound **6** (5.61 mmol; 78 %) as white

solid. R_f: 0.70 (PE/EA 1:1); Mp: 143.4 °C (*n*-hexane/ $CHCl_3$); ^{1}H-NMR ($CDCl_3$) δ 8.66 (s, 1H, H-aryl), 8.29 (s. 1H, H-aryl), 8.22 (s, 1H, H-aryl), 7.76 (d, 2H, *J* = 7.4 Hz, H-aryl), 7.59 (d, 2H, *J* = 7.3 Hz, H-aryl), 7.40 (t, 2H, *J* = 7.2 Hz, H-aryl), 7.31 (t, 2H, *J* = 7.2 Hz, H-aryl), 5.39 (s, 1H, N*H*), 4.50–4.47 (m, 4H, Fmoc-CH_2, benzene-CH_2), 4.22 (t, 1H, *J* = 6.4 Hz, Fmoc-C*H*), 1.62 (s, 9H, $CO_2C(CH_3)_3$); ^{13}C-NMR ($CDCl_3$) δ 163.5, 156.6 (2C, C=O), 148.6, 143.8, 141.4, 141.3, 134.1, 134.0, 127.2, 125.6, 125.0, 123.5, 120.1 (11C, C-aryl), 82.9 (1C, $CO_2C(CH_3)_3$), 67.1 (1C, Fmoc-CH_2), 47.3 (1C, Fmoc-*C*H), 44.2 (1C, benzene-CH_2), 28.2 (3C, $CO_2C(CH_3)_3$); FT-ICR-MS Anal. Calcd for $C_{27}H_{26}N_2O_6Na$ $[M+Na]^+$: *m/z* 497.168308; found: *m/z* 497.168682; Anal. Calcd for $C_{27}H_{26}N_2O_6$(474.51): C 68.34; H 5.52; N 5.90; found: C 68.27; H 5.51 N 5.76.

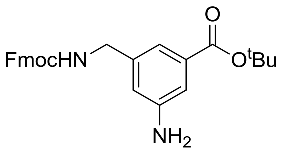

7

3-[(9*H*-Fluoren-9-ylmethoxycarbonyl)aminomethyl]-5-aminobenzoic acid *tert*-butyl ester (7)

To a suspension of 210 mg **6** (0.44 mmol; 1 eq) in 50 ml MeOH was added 50 mg lindlar catalyst. The resulting mixture was deaerated, whereupon it was submitted to a hydrogen atmosphere at normal pressure for 5 h. The final mixture was filtered through a pad of Celite and the solvent was removed under reduced pressure. Purification of the residue via column chromatography eluting with $CHCl_3$/acetone 200:1 afforded 194 mg of pure title compound **7** (0.44 mmol; 99 %) as white solid. R_f: 0.36 (PE/EA 1:1); ^{1}H-NMR ($CDCl_3$) δ .76 (d, 2H, *J* = 7.5 Hz, H-aryl), 7.60 (d, 2H, *J* = 7.4 Hz, H-aryl), 7.40 (t, 2H, *J* = 7.4 Hz, H-aryl), 7.33–7.26 (m, 4H, H-aryl), 7.21 (t, 1H,

J = 7.4 Hz, H-aryl), 6.76 (s, 1H, N*H*), 5.10 (s, 1H, N*H*), 4.45 (d, 2H, *J* = 6.9 Hz, Fmoc-C*H*$_2$), 4.33 (d, 2H, *J* = 5.9 Hz, benzene-C*H*$_2$), 4.23 (t, 1H, *J* = 6.9 Hz, Fmoc-C*H*), 3.76 (s, 2H, N*H*$_2$), 1.57 (s, 9H, $CO_2C(CH_3)_3$); ^{13}C-NMR ($CDCl_3$) δ 165.8, 156.6 (2C, C=O), 146.9, 144.0, 141.4, 139.8, 133.6, 127.8, 127.2, 125.2, 120.1, 118.6, 118.0, 115.1 (12C, C-aryl), 81.2 (1C, $CO_2C(CH_3)_3$), 66.9 (1C, Fmoc-*C*H$_2$), 47.4 (1C, Fmoc-*C*H), 45.0 (1C, benzene-*C*H$_2$), 28.3 (3C, $CO_2C(CH_3)_3$); ESI-TOF-MS Anal. Calcd for $C_{27}H_{28}N_2O_4Na$ $[M+Na]^+$: *m/z* 467.19413; found: *m/z* 467.19442.

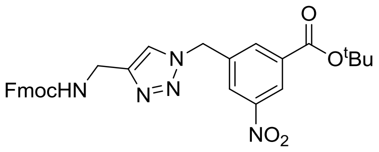

8

3-{[4-[(9*H*-Fluoren-9-ylmethoxycarbonyl)aminomethyl]-1*H*-1,2,3-triazol-1-yl]methyl}-5-nitrobenzoic acid *tert*-butyl ester (8)

To a solution of 1.00 g **5** (3.59 mmol; 1 eq) and 1.00 g Fmoc-propargylamine (3.59 mmol; 1 eq) in 40 ml DCM-^{t}BuOH (1:1) were added 0.18 g $Cu(II)SO_4 \cdot 5\ H_2O$ (0.72 mmol; 0.2 eq) and 0.29 g sodiumascorbate (1.44 mmol; 0.4 eq) in 20 ml H_2O. The mixture was stirred at 40°C for 3 d. The solvent was evaporated and the residue was dissolved in 200 ml DCM. The organic layer was washed with H_2O (1 x 20 ml), sat. NaCl-soln. (1 x 20 ml), dried over Na_2SO_4 and the solvent was removed under reduced pressure. Purification of the residue via column chromatography eluting with PE/EA 1:2 and recrystallization from *n*-hexane/$CHCl_3$ afforded 1.74 g of pure title compound **8** (3.13 mmol; 87 %) as white solid. R_f: 0.30 (PE/EA 1:2); Mp: 111.5°C (*n*-hexane/ $CHCl_3$); ^{1}H-NMR ($CDCl_3$) δ 8.73–8.72 (m, 1H, H-aryl), 8.24 (d, 2H, *J* = 15.3 Hz, H-aryl), 7.74 (d, 2H, *J* = 7.5 Hz, H-aryl), 7.56–7.53 (m, 3H, H-aryl), 7.39–7.35 (m,

2H, H-aryl), 7.26 (t, 2H, *J* = 7.38 Hz, H-aryl), 5.62–5.58 (m, 3H, benzene-C*H*$_2$, N*H*), 4.46 (d, 2H, *J* = 5.9 Hz, triazol-C*H*$_2$), 4.37 (d, 2H, *J* = 7.0 Hz, Fmoc-C*H*$_2$), 4.17 (t, 1H, *J* = 6.9 Hz, Fmoc-C*H*), 1.60 (s, 9H, $CO_2C(CH_3)_3$); ^{13}C-NMR ($CDCl_3$) δ 162.9, 156.6 (2C, C=O), 148.7, 146.1, 143.9, 141.4, 137.0, 134.8, 134.5, 127.8, 127.1, 126.4, 125.1, 124.7, 122.5, 120.1 (14C, C-aryl), 83.3 (1C, $CO_2C(CH_3)_3$), 67.0 (1C, Fmoc-*C*H$_2$), 52.9 (1C, benzene-*C*H$_2$), 47.2 (1C, Fmoc-*C*H), 36.5 (1C, triazol-*C*H$_2$), 28.2 (3C, $CO_2C(CH_3)_3$); FT-ICR-MS Anal. Calcd for $C_{30}H_{29}N_5O_6Na$ [M+Na]$^+$: *m/z* 578.201005; found: *m/z* 578.201133; Anal. Calcd for $C_{30}H_{29}N_5O_6$(555.58): C 64.85; H 5.26; N 12.61; found: C 64.70; H 5.26; N 12.59.

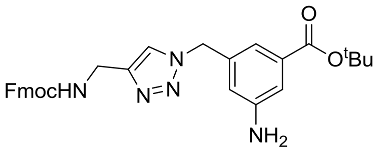

9

3-{[4-[(9*H*-Fluoren-9-ylmethoxycarbonyl)aminomethyl]-1*H*-1,2,3-triazol-1-yl]methyl}-5-aminobenzoic acid *tert*-butyl ester (9)

To a solution of 170 mg **8** (0.31 mmol; 1 eq) in 50 ml MeOH was added 50 mg Pd/C. The resulting mixture was deaerated, whereupon it was submitted to a hydrogen atmosphere at normal pressure for 4 1/2 h. The final mixture was filtered through a pad of Celite and the solvent was removed under reduced pressure. Purification of the residue via column chromatography eluting with $CHCl_3$/MeOH 60:1 afforded 141 mg of pure title compound **9** (0.27 mmol; 88 %) as white solid. R_f: 0.42 ($CHCl_3$/MeOH 60:1); ^{1}H-NMR ($CDCl_3$) δ 7.73 (d, 2H, *J* = 7.5 Hz, H-aryl), 7.55 (d, 2H, *J* = 7.3 Hz, H-aryl), 7.42 (s, 1H, H-aryl), 7.37 (t, 2H, *J* = 7.4 Hz, H-aryl), 7.27 (dd, 4H, *J* = 4.0 Hz, *J* = 12.6 Hz, H-aryl), 6.62 (s, 1H, H-aryl), 5.65 (s, 1H, N*H*), 5.39 (s, 2H, benzene-C*H*$_2$), 4.43 (d, 2H, *J* = 5.7 Hz, triazol-C*H*$_2$), 4.37 (d, 2H, *J* = 6.9 Hz, Fmoc-C*H*$_2$), 4.17 (t, 1H, *J* = 6.8 Hz, Fmoc-C*H*), 3.68 (s, 2H, N*H*$_2$), 1.57 (s, 9H, $CO_2C(CH_3)_3$); ^{13}C-NMR

$(CDCl_3)$ δ 165.3, 156.5 (2C, C=O), 147.3, 145.6, 143.9, 141.4, 135.8, 133.9, 127.8, 127.1, 125.2, 122.1, 120.1, 119.0, 117.9, 116.2 (14C, C-aryl), 81.5 (1C, $CO_2\mathit{C}(CH_3)_3$), 66.9 (1C, Fmoc-$\mathit{C}H_2$), 54.0 (1C, benzene-$\mathit{C}H_2$), 47.3 (1C, Fmoc-*C*H), 36.6 (1C, triazol-$\mathit{C}H_2$), 28.2 (3C, $CO_2C(\mathit{C}H_3)_3$); FT-ICR-MS Anal. Calcd for $C_{30}H_{31}N_5O_4Na$ $[M+Na]^+$: *m/z* 548.226826; found: *m/z* 548.226661.

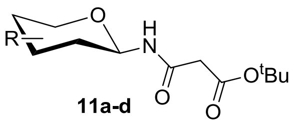

11a-d

General Procedure for synthesis of compounds 11a-d [9]

The peracetylated glycosylamin **10a-d** (1 eq) was dissolved in dry THF. The solution was cooled to 0°C and molecular sieve 3 Å was added. Afterwards a solution of mono-*tert*-butyl-malonate (1.1 eq) and HBTU (1.1 eq) in dry DMF was added. Immediately the pH was adjusted to pH 8 by adding NEt_3 and the resulting mixture was stirred at 0°C for 1 h. Thereafter the solution was stirred at r.t. for 15 h. The slurry was filtered and the solvent was removed under reduced pressure. The residue was dissolved in ethyl acetate and transferred into a separatory funnel. The organic layer was acidified with 10 % citric acid-soln., washed with sat. $NaHCO_3$-soln., sat. NaCl-soln., dried over Na_2SO_4 and the solvent was evaporated. Purification of the residue via column chromatography afforded pure title compound **11a-d** as white solid.

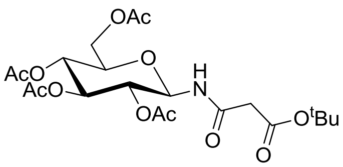

11a

N-(2,3,4,6-Tetra-*O*-acetyl-β-D-glucopyranosyl)malonamide *tert*-butylester (11a)

According to General Procedure 2,3,4,6-Tetra-*O*-acetyl-β-D-glucopyranosylamine 1.77 g **10a** (5.10 mmol; 1 eq), 0.86 ml *tert*-butyl-malonate (5.61 mmol; 1.1 eq), 2.31 g HBTU (5.61 mmol; 1.1 eq), 10 ml NEt_3 in 40 ml dry THF and 20 ml dry DMF afforded after column chromatography (PE/EA 1:1→1:2) 1.89 g (3.86 mmol; 76 %) pure title compound **11a** as white solid. R_f: 0.35 (PE/EA 1:1); Mp 137.8 °C (*n*-hexane/$CHCl_3$); $[\alpha]_D^{20}$: +7.0 (*c* 1.0, $CHCl_3$); ^{1}H-NMR ($CDCl_3$) δ 7.57 (d, 1H, $J_{1,NH}$ = 9.1 Hz, N*H*), 5.27 (m, 2H, H-3, H-1), 5.06 (t, 1H, $J_{3,4}$ = $J_{4,5}$ = 9.7 Hz, H-4), 4.99 (t, 1H, $J_{1,2}$ = $J_{2,3}$ = 9.6 Hz, H-2), 4.26 (dd, 1H, $J_{5,6a}$ = 4.3 Hz, $J_{6a,6b}$ = 12.5 Hz, H-6a), 4.07 (dd, 1H, $J_{5,6b}$ = 1.9 Hz, $J_{6a,6b}$ = 12.5 Hz, H-6b), 3.80 (ddd, 1H, $J_{5,6b}$ = 1.9 Hz, $J_{5,6a}$ = 4.2 Hz, $J_{4,5}$ = 10.1 Hz, H-5), 3.20 (s, 2H, $COCH_2CO$), 2.06, 2.02, 2.01, 1.99 (4s, 12H, CH_3), 1.44 (s, 9H, $CO_2C(CH_3)_3$); ^{13}C-NMR ($CDCl_3$) δ 170.7, 170.5, 170.1, 169.6, 167.5, 166.4 (6C, C=O), 83.0 (1C, $CO_2C(CH_3)_3$), 78.1 (C-1), 73.7 (C-5), 73.0 (C-3), 70.3 (C-2), 68.2 (C-4), 61.8 (C-6), 42.7 (1C, $COCH_2CO$), 28.0 (1C, $CO_2C(CH_3)_3$), 20.8, 20.7 (4C, CH_3); FT-ICR-MS Anal. Calcd for $C_{21}H_{31}NO_{12}Na$ $[M+Na]^+$: *m/z* 512.173847; found: *m/z* 512.174240; Anal. Calcd for $C_{21}H_{31}NO_{12}$(489.47): C 51.53; H 6.38; N 2.86; found: C 52.01; H 6.40; N 2.78.

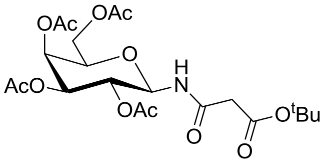

11b

***N*-(2,3,4,6-Tetra-*O*-acetyl-β-D-galctopyranosyl)malonamide *tert*-butylester (11b)**

According to General Procedure 2,3,4,6-Tetra-*O*-acetyl-β-D-galactopyranosylamine 2.13 g **10b** (6.13 mmol; 1 eq), 1.04 ml *tert*-butyl-malonate (6.74 mmol; 1.1 eq), 2.56 g HBTU (6.74 mmol; 1.1 eq), 10 ml NEt_3 in 40 ml dry THF and 20 ml dry DMF afforded after column chromatography (PE/EA 1:1→1:2) 1.68 g (3.43 mmol; 56 %) pure title compound **11b** as white solid. R_f: 0.23 (PE/EA 1:1); $[\alpha]_D^{20}$: +23.7 (*c* 1.0, $CHCl_3$); ^{1}H-NMR ($CDCl_3$) δ 7.63 (d, 1H, $J_{1,NH}$ = 8.9 Hz, N*H*), 5.42–5.41 (m, 1H, H-4), 5.25 (t, 1H, $J_{1,NH}$ = 9.1 Hz, H-1), 5.20–5.15 (m, 1H, H-2), 5.10 (dd, 1H, $J_{3,4}$ = 3.3 Hz, $J_{2,3}$ = 10.0 Hz, H-3), 4.14–4.00 (m, 3H, H-6a, H-6b, H-5), 3.21 (s, 2H, $COCH_2CO$), 2.14, 2.03, 2.02, 1.97 (4s, 12H, CH_3), 1.46 (s, 9H, CH_3); ^{13}C-NMR ($CDCl_3$) δ 170.7, 170.5, 170.2, 170.0, 167.6, 166.3 (6C, C=O), 83.0 (1C, $CO_2C(CH_3)_3$), 78.4 (C-1), 72.5 (C-5), 71.1 (C-3), 68.1 (C-2), 67.3 (C-4), 61.3 (C-6), 42.7 (1C, $COCH_2CO$), 28.1 (1C, $CO_2C(CH_3)_3$), 20.8, 20.7, 20.6 (4C, CH_3); ESI-TOF-MS: Anal. Calcd for $C_{21}H_{31}NO_{12}Na$ $[M+Na]^+$: *m/z* 512.17385; found: *m/z* 512.17357; Anal. Calcd for $C_{21}H_{31}NO_{12}$(489.47): C 51.53; H 6.38; N 2.86; found: C 51.67; H 6.35; N 2.45.

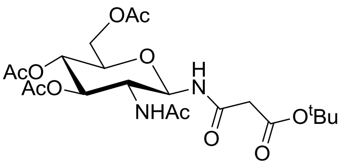

11c

N-(2-Acetamido-2-deoxy-3,4,6-tetra-O-acetyl-β-D-glucopyranosyl)malonamide *tert*-butylester (11c)

According to General Procedure 2-Acetamido-2-deoxy-3,4,6-tetra-*O*-acetyl-β-D-glucopyranosylamine 1.41 g **10c** (4.07 mmol; 1 eq), 0.69 ml *tert*-butyl-malonate (4.48 mmol; 1.1 eq), 1.70 g HBTU (4.48 mmol; 1.1 eq), 5 ml NEt_3 in 40 ml dry THF and 20 ml dry DMF afforded after column chromatography ($CHCl_3$/MeOH 100:1) 1.11 g (2.27 mmol; 56 %) pure title compound **11c** as white solid. R_f: 0.36 ($CHCl_3$/MeOH 100:1); Mp: 142.8 °C (*n*-hexane/EA); $[\alpha]_D^{20}$: + 3.0 (*c* 1.0, $CHCl_3$); ^{1}H-NMR ($CDCl_3$) δ 7.55 (d, 1H, $J_{1,NH}$ = 8.6 Hz, N*H*), 6.47 (d, 1H, $J_{2,NH}$ = 8.9 Hz, N*H*), 5.25–5.20 (m, 2H, H-1, H-3), 5.08 (t, 1H, $J_{3,4}$ = $J_{4,5}$ = 9.7 Hz, H-4), 4.27 (dd, 1H, $J_{5,6a}$ = 4.3 Hz, $J_{6a,6b}$ = 12.5 Hz, H-6a), 4.18 (dd, 1H, $J_{1,2}$ = $J_{2,3}$ = 9.9 Hz, $J_{2,NH}$ = 10.0 Hz, H-2), 4.07 (dd, 1H, $J_{5,6b}$ = 2.2 Hz, $J_{6a,6b}$ = 12.5 Hz, H-6b), 3.84 (ddd, 1H, $J_{5,6b}$ = 2.2 Hz, $J_{5,6b}$ = 4.2 Hz, $J_{4,5}$ = 10.0 Hz, H-5), 3.27–3.16 (m, 2H, COCH_2CO), 2.06, 2.01, 2.00, 1.93 (4s, 12H, CH_3), 1.43 (s, 9H, $CO_2C(CH_3)_3$); ^{13}C-NMR ($CDCl_3$) δ 172.0, 171.4, 170.8, 169.5, 167.3, 166.9 (6C, C=O), 82.6 (1C, $CO_2C(CH_3)_3$), 79.9 (C-1), 73.5 (C-5), 72.9 (C-3), 68.2 (C-4), 62.0 (C-6), 53.0 (C-2), 43.7 (1C, CO*C*H_2CO), 28.1 (1C, $CO_2C(CH_3)_3$), 23.1, 20.8, 20.7, 20.6 (4C, CH_3); ESI-TOF-MS: Anal. Calcd for $C_{21}H_{32}N_2O_{11}Na$ $[M+Na]^+$: *m/z* 511.18983; found: *m/z* 511.19053; Anal. Calcd for $C_{21}H_{32}N_2O_{11}$(488.49): C 51.63; H 6.60; N 5.73; found: C 51.49; H 6.51; N 5.60.

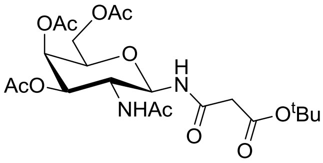

11d

_N_-(2-Acetamido-2-deoxy-3,4,6-tetra-_O_-acetyl-β-D-galactopyranosyl)malonamide _tert_-butylester (11d)

According to General Procedure 2-Acetamido-2-deoxy-3,4,6-tetra-*O*-acetyl-β-D-galactopyranosylamine 213 mg **10d** (0.63 mmol; 1 eq), 0.11 ml *tert*-butyl-malonate (0.69 mmol; 1.1 eq), 261 mg HBTU (0.69 mmol; 1.1 eq), 2 ml NEt_3 in 10 ml dry THF and 5 ml dry DMF afforded after column chromatography ($CHCl_3$/MeOH 100:1) 162 mg (0.33 mmol; 53 %) pure title compound **11d** as white solid. R_f: 0.24 ($CHCl_3$/MeOH 100:1); Mp: 115.8 °C (*n*-hexane/EA); $[\alpha]_D^{20}$: +11.4 (*c* 1.0, $CHCl_3$); ^{1}H-NMR ($CDCl_3$) δ 7.51 (d, 1H, $J_{1,NH}$ = 8.5 Hz, N*H*), 6.44 (d, 1H, $J_{2,NH}$ = 8.9 Hz, N*H*), 5.36 (d, 1H, $J_{3,4}$ = $J_{4,5}$ = 3.3 Hz, H-4), 5.25–5.19 (m, 2H, H-1, H-3), 4.34 (dd, 1H, $J_{1,2}$ = $J_{2,3}$ = 9.5 Hz, $J_{2,NH}$ = 9.1 Hz, H-2), 4.17–4.04 (m, 3H, H-6a, H-6b, H-5), 3.28–3.15 (m, 2H, $COCH_2CO$), 2.13, 2.01, 1.97, 1.93 (4s, 12H, CH_3), 1.44 (s, 9H, $CO_2C(CH_3)_3$); ^{13}C-NMR ($CDCl_3$) δ 172.3, 170.8, 170.5, 170.3, 167.6, 166.7 (6C, C=O), 82.6 (1C, $CO_2C(CH_3)_3$), 80.4 (C-1), 72.3 (C-5), 70.5 (C-3) 66.8 (C-4), 61.5 (C-6), 49.3 (C-2), 43.6 (1C, $COCH_2CO$), 28.1 (1C, $CO_2C(CH_3)_3$), 23.1, 20.8 (4C, CH_3); FT-ICR-MS: Anal. Calcd for $C_{21}H_{32}N_2O_{11}Na$ $[M+Na]^+$: *m/z* 511.189831; found: *m/z* 511.189365; Anal. Calcd for $C_{21}H_{32}N_2O_{11}$(488.49): C 51.63; H 6.60 ; N 5.73; found: C 51.35; H 6.68 ; N 5.53.

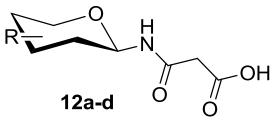

12a-d

General Procedure for *tert*-butyl ester-hydrolysis 12a-d [9]

The *tert*-Butyl ester derivate **11a-d** was stirred in TFA/DCM (3:4) at r.t. for 24 h. DCM and TFA were removed by passing a stream of N_2 through the solution. The residue was repeatedly dissolved in toluene and concentrated in vacuo (5 x 20 ml). Chromatography of the residue afforded compounds **12a-d** as white solids.

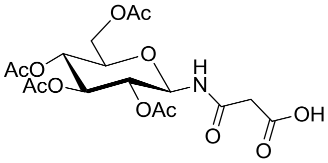

12a

2,3,4,6-Tetra-*O*-acetyl-β-D-glucopyranosylamino-3-oxomalonic acid (12a)

According to General Procedure *N*-(2,3,4,6-Tetra-*O*-acetyl-β-D-glucopyranosyl)-malonamide-*tert*-butylester 1.77 g **11a** (3.62 mmol; 1 eq) in 16 ml dry DCM and 12 ml TFA afforded after column chromatography (PE/EA 1:1→PE/EA 1:3 + 1 % HCO_2H) 1.43 g (3.29 mmol; 91 %) pure title compound **12a** as white solid. R_f: 0.52 (PE/EA 1:3 + 1 % HCO_2H); $[\alpha]_D^{20}$: + 6.5 (*c* 1.0, $CHCl_3$); ^{1}H-NMR ($CDCl_3$) δ 8.59 (s, 1H, CO_2H), 7.68 (d, 1H, $J_{1,NH}$ = 8.9 Hz, N*H*), 5.30 (t, 2H, $J_{1,2}$ = 9.4 Hz, H-1, H-3), 5.07 (t, 1H, $J_{3,4}$ = $J_{4,5}$ = 9.8 Hz, H-4), 5.01 (t, 1H, $J_{1,2}$ = $J_{2,3}$ = 9.5 Hz, H-2), 4.28 (dd, 1H, $J_{5,6b}$ = 4.5 Hz, $J_{6a,6b}$ = 12.4 Hz, H-6a), 4.09 (d, 1H, $J_{6a,6b}$ = 12.4 Hz, H-6b), 3.85 (dd, 1H, $J_{5,6b}$ = 2.0 Hz, $J_{4,5}$ = 8.0 Hz, H-5), 3.39 (s, 2H, $COCH_2CO$), 2.07, 2.04, 2.03, 2.01 (4s, 12H, CH_3); ^{13}C-NMR ($CDCl_3$) δ 171.3, 171.0, 170.2, 170.1, 169.8, 167.4 (6C, C=O), 78.0 (C-1), 73.9 (C-5), 72.9 (C-3), 70.6 (C-2), 68.2 (C-4), 61.9 (C-6), 40.8 (1C,

$COCH_2CO$), 20.8, 20.7, 20.6 (4C, CH_3); ESI-TOF-MS: Anal. Calcd for $C_{17}H_{23}NO_{12}Na$ $[M+Na]^+$: *m/z* 456.11125; found: *m/z* 456.11071.

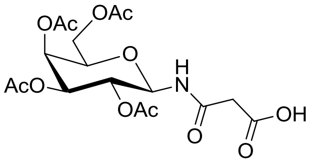

12b

2,3,4,6-Tetra-*O*-acetyl-β-D-galactopyranosylamino-3-oxomalonic acid (12b)

According to General Procedure *N*-(2,3,4,6-Tetra-*O*-acetyl-β-D-galactopyranosyl)-malonamide-*tert*-butylester 1.78 g **11b** (3.64 mmol; 1 eq) in 17 ml dry DCM and 13 ml TFA afforded after column chromatography (PE/EA 1:1→PE/EA 1:3 + 1 % HCO_2H) 1.52 g (3.51 mmol; 96 %) pure title compound **12b** as white solid. R_f: 0.53 (PE/EA 1:3 + 1 % HCO_2H); $[\alpha]_D^{20}$: + 19.8 (*c* 1.0, $CHCl_3$); ^{1}H-NMR ($CDCl_3$) δ 7.56 (d, 1H, $J_{1,NH}$ = 9.0 Hz, N*H*), 7.37 (s, 1H, CO_2H), 5.45–5.44 (m, 1H, H-4), 5.30–5.25 (m, 1H, H-1), 5.16–5.14 (m, 2H, H-2, H-3), 4.12–4.05 (m, 3H, H-6a, H-6b, H-5), 3.39 (s, 2H, $COCH_2CO$), 2.15, 2.07, 2.04, 1.99 (4s, 12H, CH_3); ^{13}C-NMR ($CDCl_3$) δ 171.6, 170.8, 170.2, 170.0, 169.9, 167.3 (6C, C=O), 78.5 (C-1), 72.6 (C-5), 70.9 (C-3), 68.3 (C-2), 67.3 (C-4), 61.3 (C-6), 40.7 (1C, $COCH_2CO$), 20.8, 20.7, 20.6 (4C, CH_3); ESI-TOF-MS: Anal. Calcd for $C_{17}H_{23}NO_{12}Na$ $[M+Na]^+$: *m/z* 456.11125; found: *m/z* 456.11071.

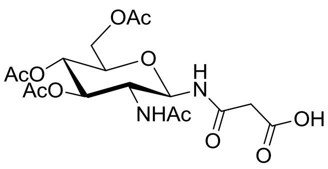

12c

2-Acetamido-2-deoxy-3,4,6-tetra-*O*-acetyl-β-D-glucopyranosylamino-3-oxomalonic acid (12c)

According to General Procedure *N*-(2-Acetamido-2-deoxy-3,4,6-tetra-*O*-acetyl-β-D-glucopyranosyl)-malonamide-*tert*-butylester 250 mg **11c** (0.51 mmol; 1 eq) in 4 ml dry DCM and 3 ml TFA afforded after column chromatography (PE/EA 1:3→EA + 1 % HCO_2H) 220 mg (0.50 mmol; 98 %) pure title compound **12c** as white solid. R_f: 0.38 (EE + 1 % HCO_2H); $[\alpha]_D^{20}$: + 13.4 (*c* 1.0, DMSO); ^{1}H-NMR (DMSO-d_6) δ 12.51 (s, 1H, CO_2H), 8.68 (d, 1H, $J_{1,NH}$ = 9.2 Hz, N*H*), 7.92 (d, 1H, $J_{2,NH}$ = 9.3 Hz, N*H*), 5.15 (t, 1H, $J_{1,2}$ = $J_{1,NH}$ = 9.5 Hz, H-1), 5.11–5.07 (m, 1H, H-3), 4.82 (t, 1H, $J_{3,4}$ = $J_{4,5}$ = 9.8 Hz, H-4), 4.17 (dd, 1H, $J_{5,6a}$ = 4.3 Hz, $J_{6a,6b}$ = 12.5 Hz, H-6a), 3.96 (dd, 1H, $J_{5,6b}$ = 2.0 Hz, $J_{6a,6b}$ = 12.4 Hz, H-6b), 3.92–3.81 (m, 2H, H-2, H-5), 3.16 (m, 2H, $COCH_2CO$), 2.00, 1.96, 1.90, 1.74 (4s, 12H, CH_3); ^{13}C-NMR (DMSO-d_6) δ 170.1, 169.5, 169.4, 169.3, 168.9, 166.3 (6C, C=O), 78.1, (C-1), 73.3 (C-3), 72.3 (C-5), 68.4 (C-4), 61.8 (C-6), 52.0 (C-2), 42.6 (1C, $COCH_2CO$), 22.6, 20.6, 20.4, 20.3 (4C, CH_3); FT-ICR-MS: Anal. Calcd for $C_{17}H_{24}N_2O_{11}Na$ $[M+Na]^+$: *m/z* 455.127231; found: *m/z* 455.127337.

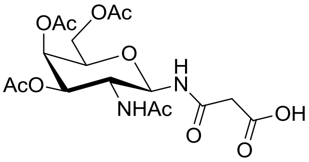

12d

2-Acetamido-2-deoxy-3,4,6-tetra-*O*-acetyl-β-D-galactopyranosylamino-3-oxomalonic acid (12d)

According to General Procedure *N*-(2-Acetamido-2-deoxy-3,4,6-tetra-*O*-acetyl-β-D-galactopyranosyl)-malonamide-*tert*-butylester 55 mg **11d** (0.11 mmol; 1 eq) in 2 ml dry DCM and 1.5 ml TFA afforded after column chromatography (PE/EA 1:5→EA + 1 % HCO_2H) 47 mg (0.11 mmol; 98 %) 47 mg (0.11 mmol; 98 %) pure title compound **12d** as white solid. R_f: 0.21 (EE + 1 % HCO_2H); $[\alpha]_D^{20}$: +20.6 (*c* 1.0, DMSO); ^{1}H-NMR (DMSO-d_6) δ 12.48 (s, 1H, CO_2H) 8.63 (d, 1H, $J_{1,NH}$ = 9.3 Hz, N*H*), 7.89 (d, 1H, $J_{2,NH}$ = 9.2 Hz, NH), 5.26 (d, 1H, $J_{3,4}$ = 3.0 Hz, H-4), 5.08 (t, 1H, $J_{1,2}$ = $J_{1,NH}$ = 9.5 Hz, H-1), 4.98 (dd, 1H, $J_{3,4}$ = 3.3 Hz, $J_{2,3}$ = 10.2 Hz, H-3), 4.11–3.94 (H-2, H-6a, H-6b, H-5), 3.20–3.09 (m, 2H, $COCH_2CO$), 2.09, 1.99, 1.89, 1.76 (4s, 12H, CH_3); ^{13}C-NMR (DMSO-d_6) δ 170.0, 169.6, 169.2, 166.6 (6C, C=O), 78.8 (C-1), 71.4 (C-5), 70.9 (C-3), 66.7 (C-4), 61.6 (C-6), 48.2 (C-2), 42.8 (1C, $COCH_2CO$), 22.7, 20.6, 20.5, 20.4 (4C, CH_3); FT-ICR-MS: Anal. Calcd for $C_{17}H_{24}N_2O_{11}Na$ $[M+Na]^+$: *m/z* 433.145286; found: 433.145250.

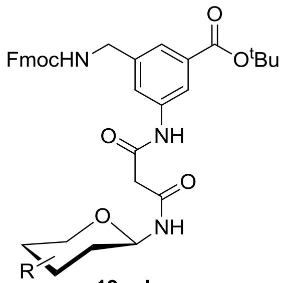

13a-d

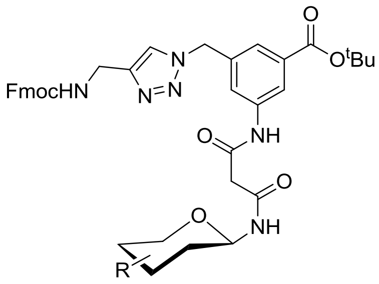

14a-d

General Procedure for synthesis of compounds 13a-d and 14a-d

In a 25 ml round bottom flask equipped with a gas inlet and a stirring bar **12a-d** (1.3 eq) was dissolved in 12 ml dry DMF under an atmosphere of nitrogen. The solution was cooled to 0°C and EDCI·HCl (1.3 eq), HOBt (1.3 eq) and DIPEA (3.9 eq) were added. The mixture was stirred at 0°C for 10 min. Afterwards **7** or **9** (1 eq) was added and the resulting solution was stirred at 0°c for 2 h. Thereafter the solution was stirred at r.t. for 15 h. The solvent was removed under reduced pressure, the residue was dissolved in ethyl acetate (70 ml) and transferred into a separatory funnel. The organic layer was acidified with 10 % citric acid-soln. (2 x 20 ml), washed with sat. $NaHCO_3$-soln. (3 x 20 ml), sat. NaCl-soln. (20 ml), dried over Na_2SO_4 and the solvent was evaporated. Purification of the residue via column chromatography afforded pure title compound **13a-d** or **14a-d** as white solid.

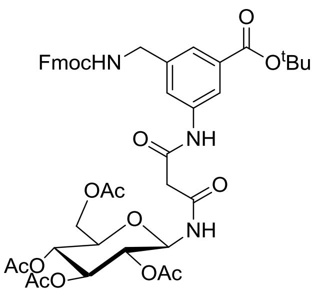

13a

3-[(9*H*-Fluoren-9-ylmethoxycarbonyl)aminomethyl]-5-[*N*-(2,3,4,6-tetra-*O*-acetyl-β-D-glucopyranosyl)malonylamino]benzoic acid *tert*-butyl ester (13a)

According to General Procedure 161 mg **7** (0.36 mmol; 1 eq) afforded after column chromatography ($CHCl_3$/MeOH 200:1) 167 mg (0.19 mmol; 54 %) pure title compound **13a** as white solid. R_f: 0.33 ($CHCl_3$/MeOH 100:1); $[\alpha]_D^{20}$: -0.7 (*c* 1.0, $CHCl_3$); ^{1}H-NMR ($CDCl_3$) δ 9.31 (s, 1H, benzene-N*H*), 7.87 (d, 2H, J = 15.3 Hz, H-aryl), 7.75 (d, 2H, J = 7.5 Hz, H-aryl), 7.66 (s, 1H, H-1N*H*COCH_2), 7.59 (d, 2H, J = 7.4 Hz, H-aryl), 7.38 (t, 2H, J = 7.3 Hz, H-aryl), 7.29 (t, 2H, J = 7.2 Hz, H-aryl), 5.47 (s, 1H, CON*H*CH_2), 5.34–5.24 (m, 2H, H-1, H-3), 5.08 (t, 1H, $J_{3,4}$ = $J_{4,5}$ = 9.8 Hz, H-4), 4.99 (t, 1H, $J_{1,2}$ = $J_{2,3}$ = 9.5 Hz, H-2), 4.41–4.39 (m, 4H, Fmoc-CH_2, benzene-CH_2), 4.27 (dd, 1H, $J_{5,6a}$ = 4.3 Hz, $J_{6a,6b}$ = 12.6 Hz, H-6a), 4.22 (t, 1H, J = 7.0 Hz, Fmoc-CH), 4.08 (dd, 1H, $J_{5,6b}$ = 1.9 Hz, $J_{6a,6b}$ = 12.5 Hz, H-6b), 3.86 (ddd, 1H, $J_{5,6b}$ = 2.1 Hz, $J_{5,6a}$ = 4.2 Hz, $J_{4,5}$ = 10.1 Hz, H-5), 3.37 (dd, 2H, J = 6.7 Hz, NHCOCH_2CO), 2.05, 2.03, 2.00 (3s, 12H, CH_3), 1.57 (s, 9H, $CO_2C(CH_3)_3$); ^{13}C-NMR ($CDCl_3$) δ 170.7, 170.6, 170.1, 169.6, 168.6, 165.2, 164.8, 156.7 (8C, C=O), 144.0, 141.3, 140.4, 138.0, 133.1, 127.8, 127.1, 125.2, 124.5, 123.0, 120.0 (11C, C-aryl), 81.7 (1C, $CO_2C(CH_3)_3$), 78.1 (C-1), 73.8 (C-3), 73.0 (C-5), 70.5 (C-2), 68.1 (C-4), 67.0 (1C, Fmoc-CH_2), 61.8 (C-6), 47.2 (1C, Fmoc-*C*H), 44.8 (1C, benzene-CH_2), 43.6 (1C, NHCOCH_2CO), 28.2 (3C, $CO_2C(CH_3)_3$), 20.8, 20.7 (4C, CH_3); ESI-TOF-MS: Anal. Calcd for $C_{44}H_{49}N_3O_{15}Na$ $[M+Na]^+$: *m/z* 882.30559; found: *m/z* 882.30656; Anal.

Calcd for $C_{44}H_{49}N_3O_{15}$(859.87): C 61.46; H 5.74; N 4.89; found: C 61.47; H 5.72; N 4.84.

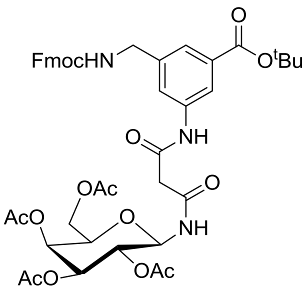

13b

3-[(9*H*-Fluoren-9-ylmethoxycarbonyl)aminomethyl]-5-[*N*-(2,3,4,6-tetra-*O*-acetyl-β-D-galactopyranosyl)malonylamino]benzoic acid *tert*-butyl ester (13b)

According to General Procedure 183 mg **7** (0.41 mmol; 1 eq) afforded after column chromatography ($CHCl_3$/MeOH 200:1) 203 mg (0.24 mmol; 57 %) pure title compound **13b** as white solid. R_f: 0.22 ($CHCl_3$/MeOH 100:1); $[\alpha]_D^{20}$: +1.5 (*c* 1.0, $CHCl_3$); ^{1}H-NMR ($CDCl_3$) δ 9.41 (s, 1H, benzene-N*H*), 7.93 (s, 1H, H-aryl), 7.84 (s, 1H, H-aryl), 7.78 (s, 1H, H-1N*H*COCH_2), 7.76–7.73 (m, 2H, H-aryl), 7.65 (s, 1H, H-aryl), 7.57 (d, 2H, *J* = 7.4 Hz, H-aryl), 7.37 (t, 2H, *J* = 7.4 Hz, H-aryl), 7.27 (t, 2H, *J* = 7.2 Hz, H-aryl), 5.57 (t, 1H, *J* = 5.9 Hz, CON*H*CH_2), 5.44–5.43 (m, 1H, H-4), 5.34–5.29 (m, 1H, H-1), 5.22–5.15 (m, 2H, H-2, H-3), 4.39 (m, 4H, Fmoc-CH_2, benzene-CH_2), 4.20 (t, 1H, *J* = 7.0 Hz, Fmoc-C*H*), 4.12–4.02 (m, 3H, H-6a, H-6b, H-5), 3.44–3.35 (m, 2H, NHCOCH_2CO), 2.12, 2.00, 1.99, 1.98 (4s, 12H, CH_3), 1.57 (s, 9H, $CO_2C(CH_3)_3$); ^{13}C-NMR ($CDCl_3$) δ 171.0, 170.5, 170.2, 169.9, 168.4, 165.2, 164.7, 156.7 (8C, C=O), 144.0, 141.4, 140.0, 138.1, 133.2, 127.8, 125.2, 124.6, 123.1, 120.1 (10C, C-aryl), 81.7 (1C, $CO_2C(CH_3)_3$), 78.5 (C-1), 72.5 (C-5), 71.0 (C-3), 68.2 (C-2), 67.3 (C-4), 67.1 (1C, Fmoc-CH_2), 61.3 (C-6), 47.3 (1C, Fmoc-CH), 44.8 (1C, benzene-CH_2), 43.6 (1C, NHCOCH_2CO), 28.3 (1C, $CO_2C(CH_3)_3$), 20.8, 20.7, 20.6,

20.5 (4C, CH_3); FT-ICR-MS: Anal. Calcd for $C_{44}H_{49}N_3O_{15}Na$ $[M+Na]^+$: *m/z* 882.305589; found: *m/z* 882.305686.

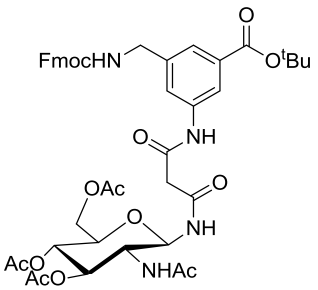

13c

3-[(9*H*-Fluoren-9-ylmethoxycarbonyl)aminomethyl]-5-[*N*-(2-Acetamido-2-deoxy-3,4,6-tetra-*O*-acetyl-β-D-glucopyranosyl)malonylamino]benzoic acid *tert*-butyl ester (13c)

According to General Procedure 178 mg **7** (0.40 mmol; 1 eq) afforded after column chromatography ($CHCl_3$/MeOH 200:1→50:1) 166 mg (0.19 mmol; 48 %) pure title compound **13c** as white solid. R_f: 0.30 ($CHCl_3$/MeOH 50:1); $[\alpha]_D^{20}$: -9.0 (*c* 1.0, $CHCl_3$); ^{1}H-NMR ($CDCl_3$) δ 9.55 (s, 1H, benzene-N*H*), 8.07 (d, 1H, $J_{1,NH}$ = 8.2 Hz, H-1N*H*$COCH_2$), 7.82 (s, 1H, H-aryl), 7.74–7.71 (m, 3H, H-aryl), 7.58–7.55 (m, 3H, H-aryl) 7.36 (t, 2H, J = 7.4 Hz, H-aryl), 7.28–7.24 (m, 2H, H-aryl), 6.86 (d, 1H, $J_{2,NH}$ = 8.6 Hz, N*H*), 5.77 (t, 1H, *J* = 5.8 Hz, CON*H*CH_2), 5.25–5.18 (m, 2 H, H-1, H-3), 5.09 (t, 1H, $J_{3,4}$ = $J_{4,5}$ = 9.6 Hz, H-4), 4.39 (d, 2H, *J* = 7.1 Hz, Fmoc-CH_2), 4.32–4.18 (m, 5 H, benzene-CH_2, H-2, H-6a, Fmoc-C*H*), 4.05 (dd, 1H, $J_{5,6b}$ = 1.8 Hz, $J_{6a,6b}$ = 12.4 Hz, H-6b), 3.78–3.75 (m, 1H, H-5), 3.43–3.32 (m, 2H, NHCOCH_2CO), 2.03, 2.02, 2.00, 1.91 (4s, 12H, CH_3), 1.55 (s, 9H, $CO_2C(CH_3)_3$); ^{13}C-NMR ($CDCl_3$) δ 172.5, 171.4, 170.8, 169.5, 169.3, 165.4, 164.5, 156.8 (8C, C=O), 144.0, 141.4, 139.8, 132.9, 127.8, 127.2, 125.3, 124.2, 122.8, 120.1, 119.6 (11C, C-aryl), 81.7 (1C, $CO_2C(CH_3)_3$), 80.0 (C-1), 73.7 (C-5), 72.9 (C-3), 68.2 (C-4), 67.1 (1C, Fmoc-CH_2), 61.9 (C-6), 52.9 (C-2), 47.3 (1C, Fmoc-CH), 44.8 (1C, benzene-CH_2), 44.4 (1C,

NHCOCH_2CO), 28.2 (3C, $CO_2C(CH_3)_3$), 28.2, 23.1, 20.8, 20.7 (4C, CH_3); FT-ICR-MS: Anal. Calcd for $C_{44}H_{50}N_4O_{14}Na$ $[M+Na]^+$: *m/z* 881.321573; found *m/z* 881.320928; Anal. Calcd for $C_{44}H_{50}N_4O_{14}$(858.89): C 61.53; H 5.87; N 6.52; found: C 61.22; H 5.86; N 6.36.

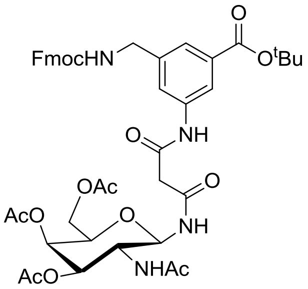

13d

3-[(9*H*-Fluoren-9-ylmethoxycarbonyl)aminomethyl]-5-[*N*-(2-Acetamido-2-deoxy-3,4,6-tetra-*O*-acetyl-β-D-galactopyranosyl)malonylamino]benzoic acid *tert*-butyl ester (13d)

According to General Procedure 194 mg **7** (0.44 mmol; 1 eq) afforded after column chromatography ($CHCl_3$/MeOH 200:1→50:1) 228 mg (0.27 mmol; 61 %) pure title compound **13d** as white solid. R_f: 0.21 ($CHCl_3$/MeOH 100:1); $[\alpha]_D^{20}$: -15.0 (*c* 1.0, $CHCl_3$); ^{1}H-NMR ($CDCl_3$) δ 9.58 (s, 1H, benzene-N*H*), 7.97 (d, 1H, $J_{1,NH}$ = 8.1 Hz, H-1N*H*COCH_2), 7.82 (s, 1H, H-aryl), 7.74–7.69 (m, 3H, H-aryl), 7.59-7.54 (m, 2H, H-aryl), 7.37 (t, 2H, *J* = 7.4 Hz, H-aryl), 7.28–7.25 (m, 3H, H-aryl), 6.86 (d, 1H, *J* = 8.4 Hz, N*H*), 5.67 (t, 1H, *J* = 5.6 Hz, CON*H*CH_2), 5.38 (d, 1H, $J_{3,4}$ = $J_{4,5}$ = 3.0 Hz, H-4), 5.20–5.14 (m, 2H, H-1, H-3), 4.45–4.27 (m, 5H, H-2, Fmoc-CH_2, benzene-CH_2), 4.20 (t, 1H, *J* = 7.0 Hz, Fmoc-C*H*), 4.14–3.98 (m, 3H, H-6a, H-6b, H-5), 3.48–3.32 (m, 2H, NHCOCH_2CO), 2.13, 2.00, 2.98, 2.93 (4s, 12H, CH_3), 1.54 (s, 9H, $CO_2C(CH_3)_3$); ^{13}C-NMR ($CDCl_3$) δ 171.0, 170.5, 170.3, 169.2, 165.5, 164.4, 156.7 (8C, C=O), 144.0, 141.4, 139.7, 138.5, 132.8, 132.7, 127.8, 127.2, 125.2, 124.2, 122.9, 120.1, 119.6 (13C, C-aryl), 81.8 (1C, $CO_2C(CH_3)_3$), 80.7 (C-1), 72.4 (C-5), 70.6 (C-3), 67.1 (1C,

Fmoc-*C*H_2), 66.6 (C-4), 61.4 (C-6), 49.3 (C-2), 47.3 (1C, Fmoc-*C*H), 44.9 (1C, benzene-*C*H_2), 44.8 (1C, NHCO*C*H_2CO), 28.2 (3C, $CO_2C(CH_3)_3$), 23.2, 20.8, 20.7 (4C, *C*H_3); FT-ICR-MS: Anal. Calcd for $C_{44}H_{50}N_4O_{14}Na$ $[M+Na]^+$: *m/z* 881.321573; found *m/z* 881.322324; Anal. Calcd for $C_{44}H_{50}N_4O_{14}$(858.89): C 61.53; H 5.87; N 6.52; found: C 61.15; H 5.82; N 6.14.

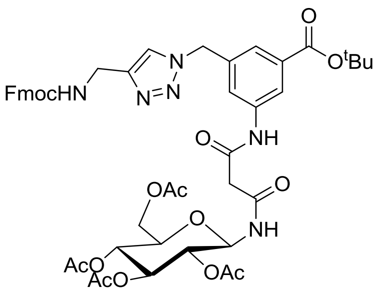

14a

3-{[4-[(9*H*-Fluoren-9-ylmethoxycarbonyl)aminomethyl]-1*H*-1,2,3-triazol-1-yl]methyl}-5-[*N*-(2,3,4,6-tetra-*O*-acetyl-β-D-glucopyranosyl)malonylamino]benzoic acid *tert*-butyl ester (14a)

According to General Procedure 118 mg **9** (0.22 mmol; 1 eq) afforded after column chromatography ($CHCl_3$/MeOH 100:1→50:1) 130 mg (0.14 mmol; 62 %) pure title compound **14a** as white solid. R_f: 0.28 ($CHCl_3$/MeOH 50:1); $[\alpha]_D^{20}$: -0.3 (*c* 1.0, $CHCl_3$); ^{1}H-NMR ($CDCl_3$) δ 9.8 (s, 1H, benzene-N*H*), 8.12–8.06 (m, 2H, H-aryl), 7.81–7.70 (m, 3H, H-aryl), 7.62 (s, 1H, H-1N*H*COCH_2), 7.55–7.51 (m, 3H, H-aryl), 7.34 (t, 2H, *J* = 7.4 Hz, H-aryl), 7.22 (t, 2H, *J* = 7.4 Hz, H-aryl), 6.05 (t, 1H, *J* = 5.6 Hz, CON*H*CH_2), 5.47–5.30 (m, 4H, triazole-C*H*$_2$, H-1, H-3), 5.11–5.03 (m, 2H, H-4, H-2), 4.40 (d, 2H, *J* = 5.7 Hz, Fmoc-C*H*$_2$), 4.32 (d, 2H, *J* = 7.0 Hz, benzene-C*H*$_2$), 4.25 (dd, 1H, $J_{5,6a}$ = 4.2 Hz, $J_{6a,6b}$ = 12.5 Hz, H-6a), 4.13 (t, 1H, *J* = 7.0 Hz, Fmoc-C*H*), 4.08–4.05 (m, 1H, H-6b), 3.89–3.87 (m, 1H, H-5), 3.40 (s, 2H, NHCOC*H*$_2$CO), 2.01 (s, 6H, C*H*$_3$), 1.99 (s, 3H, C*H*$_3$), 1.96 (s, 3H, C*H*$_3$), 1.55 (s, 9H, $CO_2C(CH_3)_3$); ^{13}C-NMR ($CDCl_3$) δ 170.7, 170.6, 170.1, 169.6, 168.4, 165.2, 164.7, 156.7 (8C, C=O), 145.8,

143.9, 141.3, 138.8, 135.6, 133.6, 127.8, 127.1, 125.2, 124.8, 123.2, 122.7, 121.1, 120.0 (14C, arom. C), 82.0 (1C, $CO_2C(CH_3)_3$), 78.1 (C-1), 73.7 (C-3), 73.0 (C-5), 70.5 (C-2), 68.1 (C-4), 66.9 (1C, Fmoc-CH_2), 61.7 (C-6), 53.8 (1C, triazole-CH_2), 47.2 (1C, Fmoc-*C*H), 43.6 (1C, $NHCOCH_2CO$), 36.4 (1C, benzene-CH_2), 28.2 (3C, $CO_2C(CH_3)_3$), 20.8, 20.7 (4C, CH_3); FT-ICR-MS: Anal. Calcd for $C_{47}H_{52}N_6O_{15}Na$ $[M+Na]^+$: *m/z* 963.338286; found: *m/z* 963.338667.

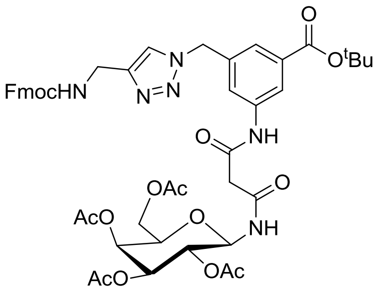

14b

3-{[4-[(9*H*-Fluoren-9-ylmethoxycarbonyl)aminomethyl]-1*H*-1,2,3-triazol-1-yl]methyl}-5-[*N*-(2,3,4,6-tetra-*O*-acetyl-β-D-galactopyranosyl)malonylamino]benzoic acid *tert*-butyl ester (14b)

According to General Procedure 156 mg **9** (0.29 mmol; 1 eq) afforded after column chromatography ($CHCl_3$/MeOH 100:1→50:1) 126 mg (0.13 mmol; 45 %) pure title compound **14b** as white solid. R_f: 0.27 ($CHCl_3$/MeOH 50:1); $[\alpha]_D^{20}$: +2.5 (*c* 1.0, $CHCl_3$); ^{1}H-NMR ($CDCl_3$) δ 9.76 (s, 1H, benzene-N*H*), 8.18 (s, 1H, H-aryl), 7.91 (d, 1H, $J_{1,NH}$ = 8.9 Hz, H-1N*H*$COCH_2$), 7.77 (s, 1H, H-aryl), 7.72 (d, 1H, *J* = 7.5 Hz, H-aryl), 7.65 (s, 1H, H-aryl), 7.55–7.53 (m, 3H, H-aryl), 7.36 (t, 2H, *J* = 7.4 Hz, H-aryl), 7.24 (t, 2H, *J* = 7.3 Hz, H-aryl), 5.94 (t, 1H, *J* = 5.7 Hz, CON*H*CH_2), 5.49 (s, 2H, triazole-CH_2), 5.45–5.44 (m, 1H, H-4), 5.34–5.29 (m, 1H, H-1), 5.24–5.16 (m, 2H, H-3, H-2), 4.42 (d, 2H, *J* = 5.7 Hz, Fmoc-CH_2), 4.34 (d, 2H, *J* = 7.0 Hz, benzene-CH_2), 4.17–4.03 (m, 4H, Fmoc-C*H*, H-6a, H-6b, H-5), 3.40 (s, 2H, $NHCOCH_2CO$), 2.13, 1.99, 1.98 (3 s, 12H, CH_3), 1.57 (s, 9H, $CO_2C(CH_3)_3$); ^{13}C-NMR ($CDCl_3$) δ 170.9,

170.5, 170.2, 170.0, 168.2, 156.6 (8C, C=O), 145.8, 143.9, 141.4, 138.9, 133.7, 127.8, 127.1, 125.2, 124.8, 123.3, 122.7, 121.3, 120.1 (13C, C-aryl), 82.1 (1C, $CO_2C(CH_3)_3$), 78.5 (C-1), 72.5 (C-5), 71.0 (C-3), 68.2 (C-2), 67.3 (C-4), 67.0 (1C, Fmoc-CH_2), 61.3 (C-6), 53.9 (1C, triazole-CH_2), 47.3 (1C, Fmoc-*C*H), 43.5 (1C, COCH_2CO), 36.5 (1C, benzene-CH_2), 28.2 (3C, $CO_2C(CH_3)_3$), 20.8, 20.7, 20.6 (4C, CH_3); FT-ICR-MS: Anal. Calcd for $C_{47}H_{52}N_6O_{15}Na$ $[M+Na]^+$: *m/z* 963.338286; found: *m/z* 963.337953.

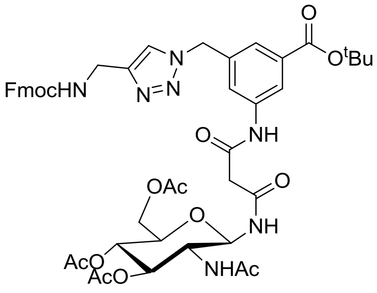

14c

3-{[4-[(9*H*-Fluoren-9-ylmethoxycarbonyl)aminomethyl]-1*H*-1,2,3-triazol-1-yl]methyl}-5-[*N*-(2-Acetamido-2-deoxy-3,4,6-tetra-*O*-acetyl-β-D-glucopyranosyl)malonylamino]benzoic acid *tert*-butyl ester (14c)

According to General Procedure 91 mg **9** (0.17 mmol; 1 eq) afforded after column chromatography ($CHCl_3$/MeOH 100:1→50:1) 108 mg (0.11 mmol; 67 %) pure title compound **14c** as white solid. R_f: 0.19 ($CHCl_3$/MeOH 50:1); $[\alpha]_D^{20}$: -15.6 (*c* 1.0, $CHCl_3$); ^{1}H-NMR ($CDCl_3$) δ 9.82 (s, 1H, benzene-N*H),* 8.13 (s, 1H, H-aryl), 8.02 (d, 1H, $J_{1,NH}$ = 8.0 Hz, H-1N*H*$COCH_2$), 7.72 (d, 2H, *J* = 7.6 Hz, H-aryl), 7.57–7.53 (m, 2H, H-aryl), 7.47 (s, 1H, H-aryl), 7.36 (t, 2H, *J* = 7.5 Hz, H-aryl), 7.32 (s, 1H, N*H*), 7.23 (t, 2H, *J* = 7.5 Hz, H-aryl), 6.17 (s, 1H, CON*H*CH_2), 5.51–5.33 (m, 3 H, triazole-CH_2, H-3), 5.22 (t, 1H, $J_{1,NH}$ = 8.0 Hz, H-1), 5.10 (t, 1H, $J_{3,4}$ = 9.7 Hz, H-4), 4.50–4.38 (m, 2H, benzene-CH_2), 4.34–4.27 (m, 3H, Fmoc-CH_2, H-2), 4.24 (dd, 1H, $J_{5,6a}$ = 4.16 Hz, $J_{6a,6b}$ = 12.5 Hz, H-6a), 4.16 (t, 1H, *J* = 7.1 Hz, Fmoc-C*H*), 4.09–4.06 (m, 1H, H-

6b), 3.84–3.81 (m, 1H, H-5), 3.41–3.26 (m, 2H, NHCOC*H*$_2$CO), 2.05, 2.03, 2.01, 1.98 (4s, 12H, C*H*$_3$), 1.56 (s, 9H, $CO_2C(CH_3)_3$); ^{13}C-NMR ($CDCl_3$) δ 173.1, 171.5, 170.8, 168.5, 169.4, 164.8, 164.4, 156.7 (8C, C=O), 145.7, 143.9, 141.4, 139.2, 135.5, 133.2, 133.1, 127.8, 127.1, 125.2, 124.0, 122.9, 122.3, 120.1 (14C, arom. C), 81.9 (1C, $CO_2C(CH_3)_3$), 80.3 (C-1), 73.6 (C-5), 72.8 (C-3), 68.4 (C-4), 67.0 (1C, Fmoc-*C*H$_2$), 61.9 (C-6), 53.8 (1C, triazole-*C*H$_2$), 52.8 (C-2), 47.3 (1C, Fmoc-*C*H), 45.1 (1C, NHCO*C*H$_2$CO), 36.4 (1C, benzene-*C*H$_2$), 28.3 (3C, $CO_2C(CH_3)_3$), 23.2, 20.9, 20.8, 20.7 (4C, *C*H$_3$); FT-ICR-MS: Anal. Calcd for $C_{47}H_{53}N_7O_{14}Na$ $[M+Na]^+$: *m/z* 962.354271; found: *m/z* 962.353677.

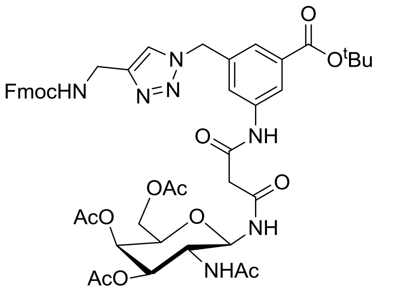

14d

3-{[4-[(9*H*-Fluoren-9-ylmethoxycarbonyl)aminomethyl]-1*H*-1,2,3-triazol-1-yl]methyl}-5-[*N*-(2-Acetamido-2-deoxy-3,4,6-tetra-*O*-acetyl-β-D-galactopyranosyl)malonylamino]benzoic acid *tert*-butyl ester (14d)

According to General Procedure100 mg **9** (0.19 mmol; 1 eq) afforded after column chromatography ($CHCl_3$/MeOH 100:1→25:1) 117 mg (0.12 mmol; 65 %) pure title compound **14d** as white solid. R_f: 0.20 ($CHCl_3$/MeOH 50:1); $[\alpha]_D^{20}$: -17.6 (*c* 1.0, $CHCl_3$); ^{1}H-NMR ($CDCl_3$) δ 9.87 (s, 1H, benzene-N*H*), 8.22 (s, 1H, H-aryl), 7.95 (d, 1H, $J_{1,NH}$ = 9.4 Hz, H-1N*H*COCH$_2$), 7.74–7.72 (m, 2H, H-aryl), 7.56–7.54 (m, 3H, H-aryl), 7.46–7.42 (m, 2H, H-aryl, N*H*), 7.36 (t, 2H, *J* = 7.4 Hz, H-aryl), 7.24 (t, 2H, *J* = 7.4 Hz, H-aryl), 7.05 (s, 1H, H-aryl), 6.15 (t, 1H, *J* = 5.6 Hz, CON*H*CH$_2$), 5.55–5.43 (m, 3H, triazole-C*H*$_2$, H-3, H-4), 5.29–5.25 (m, 1 H, triazole-C*H*$_2$), 5.14 (dd, 1H, $J_{1,2}$ =

8.2 Hz, $J_{1,NH}$ = 9.4 Hz, H-1), 4.55–4.30 (m, 5 H, H-2, benzene-C*H*$_2$, Fmoc-C*H*$_2$), 4.18–4.02 (m, 4H, Fmoc-C*H*, H-6a, H-6b, H-5), 3.47–3.22 (m, 2H, NHCOC*H*$_2$CO), 2.16, 2.09, 2.03, 1.96 (4s, 12H, C*H*$_3$), 1.58 (s, 9H, $CO_2C(CH_3)_3$); ^{13}C-NMR ($CDCl_3$) δ 173.9, 171.1, 170.6, 170.4, 169.8, 164.9, 164.2, 156.7 (8C, C=O), 145.9, 140.0, 143.9, 141.4, 139.4, 133.0, 132.0, 127.8, 127.1, 125.2, 123.7, 122.8, 120.1 (13C, C-aryl), 81.8 (1C, $CO_2C(CH_3)_3$), 81.1 (C-1), 72.4 (C-5), 70.5 (C-3), 67.0 (1C, Fmoc-*C*H$_2$), 66.7 (C-4), 61.5 (C-6), 53.8 (1C, triazole-*C*H$_2$), 49.0 (C-2), 47.3 (1C, Fmoc-*C*H), 45.8 (1C, benzene-*C*H$_2$), 28.3 (3C, $CO_2C(CH_3)_3$), 23.4, 20.9, 20.8, 20.7 (4C, *C*H$_3$); FT-ICR-MS: Anal. Calcd for $C_{47}H_{53}N_7O_{14}Na$ $[M+Na]^+$: *m/z* 962.354271; found: *m/z* 962.354687.

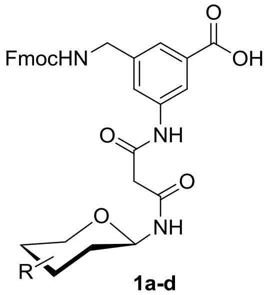

1a-d

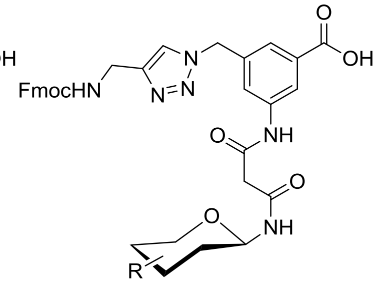

2a-d

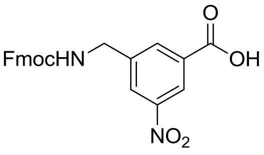

15

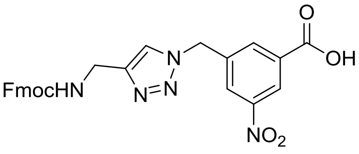

18

General Procedure for *tert*-butyl ester-hydrolysis 1a-d, 2a-d, 15 and 18.

The *tert*-Butyl ester derivate **6**, **8**, **13a-d** or **14a-d** (1 eq) was stirred in HCO_2H/DCM (2:1) at r.t. for 14 h. DCM and HCO_2H were removed by passing a stream of N_2 through the solution. The residue was repeatedly dissolved in toluene and concentrated in vacuo (5 x 20 ml). Chromatography of the residue afforded compounds **1a-d**, **2a-d, 15** or **18** as white solids.

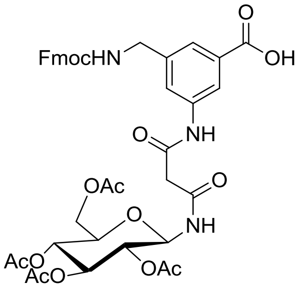

1a

3-[(9*H*-Fluoren-9-ylmethoxycarbonyl)aminomethyl]-5-[*N*-(2,3,4,6-tetra-*O*-acetyl-β-D-glucopyranosyl)malonylamino]benzoic acid (1a)

According to General Procedure 87 mg **13a** (0.10 mmol; 1 eq) in 3 ml HCO_2H/DCM (2:1) afforded after column chromatography (PE/acetone 1:1→PE/acetone 1:1 + 1 % HCO_2H) 77 mg (0.09 mmol; 95 %) pure title compound **1a** as white solid. R_f: 0.29 (PE/acetone 1:1 + 1 % HCO_2H); $[\alpha]_D^{20}$: +8.5 (*c* 1.0, DMSO); ^{1}H-NMR (DMSO-d_6) δ 13.01 (s, 1H, CO_2H), 10.31 (s, 1H, benzene-N*H*), 8.89 (d, 1H, $J_{1,NH}$ = 9.2 Hz, H-1N*H*COCH$_2$), 8.14 (s, 1H, H-aryl), 7.98 (s, 1H, CON*H*CH$_2$), 7.88 (d, 2H, *J* = 7.2 Hz, H-aryl), 7.69 (s, 3H, H-aryl), 7.59 (s, 1H, H-aryl), 7.41 (t, 2 H, *J* = 7.2 Hz, H-aryl), 7.32 (t, 2H, *J* = 7.3 Hz, H-aryl), 5.45–5.34 (m, 2H, H-1, H-3), 4.94–4.83 (m, 2H, H-4, H-2), 4.32–4.11 (m, 7H, Fmoc-CH_2, Fmoc-C*H*, benzene-CH_2, H-6a, H-5), 3.99 (d, 1H, $J_{6a,6b}$ = 11.5 Hz, H-6b), 3.30 (s, 2H, NHCOCH_2CO), 2.00 (s, 6H, CH_3), 1.96, 1.93 (2s, 6H, CH_3); ^{13}C-NMR (DMSO-d_6) δ 170.0, 169.5, 169.4, 169.2, 167.2, 167.0, 165.3, 156.4 (8C, C=O), 143.9, 140.9, 140.7, 139.1, 131.6, 127.6, 127.1, 125.2, 123.0, 121.8, 120.1, 118.5 (12C, C-aryl), 76.9 (C-1), 72.8 (C-3), 72.2 (C-5), 70.6 (C-2), 67.9 (C-4), 65.6 (1C, Fmoc-CH_2), 61.8 (C-6), 46.7 (1C, Fmoc-*C*H), 44.7 (1C, benzene-*C*H$_2$), 43.7 (1C, NHCO*C*H$_2$CO), 20.6, 20.4, 20.3 (4C, *C*H$_3$); ESI-TOF-MS: Anal. Calcd for $C_{40}H_{41}N_3O_{15}Na$ $[M+Na]^+$: *m/z* 826.24299; found: *m/z* 826.24282; Anal. Calcd for $C_{40}H_{41}N_3O_{15}$(803.76): C 59.77; H 5.23; N 5.23; found: C 59.51; H 5.16; N 4.82.

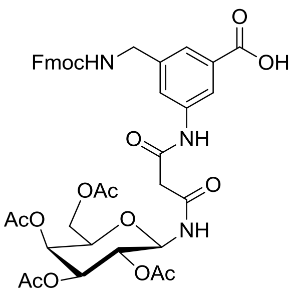

1b

3-[(9*H*-Fluoren-9-ylmethoxycarbonyl)aminomethyl]-5-[*N*-(2,3,4,6-tetra-*O*-acetyl-β-D-galactopyranosyl)malonylamino]benzoic acid (1b)

According to General Procedure 152 mg **13b** (0.18 mmol; 1 eq) in 6 ml HCO_2H/DCM (2:1) afforded after column chromatography ($CHCl_3$ + 1 % HCO_2H) 126 mg (0.16 mmol; 90 %) pure title compound **1b** as white solid. R_f: 0.45 ($CHCl_3$ + 1 % HCO_2H); $[\alpha]_D^{20}$: -6.8 (*c* 1.0, $CHCl_3$); ^{1}H-NMR (DMSO-d_6) δ 12.97 (s, 1H, CO_2H), 10.26 (s, 1H, benzene-N*H*), 8.93 (d, 1H, $J_{1,NH}$ = 9.6 Hz, H-1N*H*COCH_2), 8.14 (s, 1H, H-aryl), 7.99 (t, 1H, *J* = 6.1 Hz, CON*H*CH_2), 7.89 (d, 2H, *J* = 7.5 Hz, H-aryl), 7.70–7.68 (m, 3H, H-aryl), 7.58 (s, 1H, H-aryl), 7.41 (t, 2H, *J* = 7.4 Hz, H-aryl), 7.32 (t, 2H, *J* = 7.3 Hz, H-aryl), 5.38 (t, 1H, $J_{1, NH}$ = 9.5 Hz, H-1), 5.31–5.28 (m, 2H, H-3, H-4), 5.06–5.01 (m, 1H, H-2), 4.35–4.30 (m, 3H, H-5, Fmoc-CH_2), 4.25–4.20 (m, 3H, Fmoc-C*H*, benzene-CH_2), 4.04 (dd, 1H, $J_{5,6a}$ = 5.6 Hz, $J_{6a,6b}$ = 11.3 Hz, H-6a), 3.97 (dd, 1H, $J_{5,6b}$ = 6.8 Hz, $J_{6a,6b}$ = 11.3 Hz, H-6b), 3.32–3.24 (m, 2H, NHCOCH_2CO), 2.11, 1.99, 1.98, 1.91 (4s, 12H, CH_3); ^{13}C-NMR (DMSO-d_6) δ 170.0, 169.9, 169.5, 169.4, 167.1, 167.0, 165.3, 156.4 (8C, C=O), 143.9, 141.0, 140.7, 139.1, 131.3, 127.7, 127.1, 125.2, 123.0, 121.9, 120.1, 118.5 (12C, C-aryl), 77.16 (C-1), 71.4 (C-5), 70.8 (C-3), 68.2 (C-2), 67.6 (C-4), 65.6 (1C, Fmoc-*C*H_2), 61.5 (C-6), 46.7 (1C, Fmoc-CH), 44.6 (1C, benzene-*C*H_2), 43.7 (1C, NHCO*C*H_2CO), 20.6, 20.5, 20.4 (4C, *C*H_3); FT-ICR-MS: Anal. Calcd for $C_{40}H_{41}N_3O_{15}Na$ $[M+Na]^+$: *m/z* 826.242989; found: *m/z* 826.242409;

Anal. Calcd for $C_{40}H_{41}N_3O_{15}$(803.76): C 59.77; H 5.23; N 5.23; found: C 59.33; H 5.19; N 4.87.

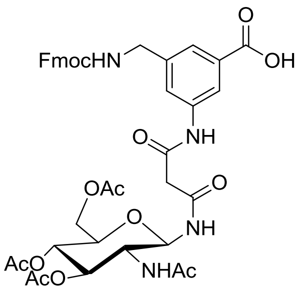

1c

3-[(9*H*-Fluoren-9-ylmethoxycarbonyl)aminomethyl]-5-[*N*-(2-Acetamido-2-deoxy-3,4,6-tetra-*O*-acetyl-β-D-glucopyranosyl)malonylamino]benzoic acid (1c)

According to General Procedure 110 mg **13c** (0.13 mmol; 1 eq) in 4.5 ml HCO_2H/DCM (2:1) afforded after column chromatography ($CHCl_3$ + 1 % HCO_2H) 70 mg (0.09 mmol; 68 %) pure title compound **1c** as white solid. R_f: 0.19 ($CHCl_3$ + 1 % HCO_2H); $[\alpha]_D^{20}$: +4.5 (*c* 1.0, DMSO); ^{1}H-NMR (DMSO-d_6) δ 12.99 (s, 1H, CO_2H), 10.26 (s. 1H, benzene-N*H*), 8.72 (d, 1H, $J_{1,NH}$ = Hz, 9.2 Hz, H-1N*H*COCH$_2$), 8.14 (s, 1H, H-aryl), 8.00–7.95 (m, 2H, CON*H*CH$_2$, N*H*), 7.90 (s, 1H, H-aryl), 7.88 (s, 1H, H-aryl), 7.71–7.67 (m, 3H, H-aryl), 7.58 (s, 1H, H-aryl), 7.41 (t, 2H, *J* = 7.4 Hz, H-aryl), 7.32 (t, 2H, *J* = 7.4 Hz, H-aryl), 5.18 (t, 1H, $J_{1,NH}$ = 9.5 Hz, H-1), 5.11 (t, 1H, $J_{3,4}$ = 9.8 Hz, H-3), 4.84 (t, 1H, $J_{3,4}$ = 9.8 Hz, H-4), 4.31 (d, 2H, *J* = 7.0 Hz, Fmoc-C*H*$_2$), 4.25–4.16 (m, 4H, Fmoc-C*H*, benzene-C*H*$_2$, H-6b), 3.98–3.90 (m, 2H, H-6b, H-2), 3.88–3.83 (m, 1H, H-5), 3.29 (s, 2H, NHCOC*H*$_2$CO), 2.00, 1.97, 1.91, 1.75 (4s, 12H, C*H*$_3$); ^{13}C-NMR (DMSO-d_6) δ 170.1, 169.6, 169.5, 169.4, 167.1, 167.1, 165.3, 156.4 (8C, C=O), 143.9, 141.0, 140.7, 139.1, 131.4, 127.7, 127.1, 125.2, 123.0, 121.9, 120.2, 118.5 (12CC-aryl), 78.1 (C-1), 73.3 (C-3), 72.3 (C-5), 68.4 (C-4), 65.6 (1C, Fmoc-*C*H$_2$), 61.8 (C-6), 52.0 (C-2), 46.7 (1C, Fmoc-*C*H), 44.64 (benzene-*C*H$_2$), 43.7 (1C,

NHCOCH_2CO), 22.6, 20.6, 20.4, 20.4 (4C, CH_3); ESI-TOF-MS: Anal. Calcd for $C_{40}H_{42}N_4O_{14}Na$ [M+Na]$^+$: *m/z* 801.26248; found: *m/z* 801.26279.

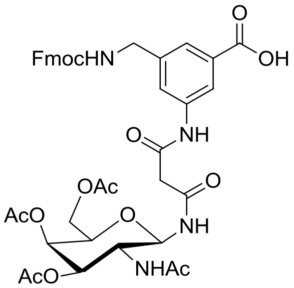

1d

3-[(9*H*-Fluoren-9-ylmethoxycarbonyl)aminomethyl]-5-[*N*-(2-Acetamido-2-deoxy-3,4,6-tetra-*O*-acetyl-β-D-galactopyranosyl)malonylamino]benzoic acid (1d)

According to General Procedure 176 mg **13d** (0.20 mmol; 1 eq) in 6 ml HCO_2H/DCM (2:1) afforded after column chromatography ($CHCl_3$/MeOH 50:1→ $CHCl_3$/MeOH 10:1 + 1 % HCO_2H) 155 mg (0.19 mmol; 94 %) pure title compound **1d** as white solid. R_f: 0.37 ($CHCl_3$/MeOH 10:1 + 1 % HCO_2H); $[\alpha]_D^{20}$: +12.2 (*c* 1.0, DMSO); ^{1}H-NMR (DMSO-d_6) δ 13.12 (s, 1H, CO_2H), 20.23 (s, 1H, benzene-N*H*), 8.66 (d, 1H, $J_{1,NH}$ = 9.2 Hz, H-1N*H*COCH$_2$), 8.12 (s, 1H, H-aryl), 7.99 (t, 1H, *J* = 6.1 Hz, CON*H*CH$_2$), 7.94 (d, 1H, *J* = 9.1 Hz, N*H*), 7.90–7.88 (m, 2H, H-aryl), 7.71–7.67 (m, 3H, H-aryl), 7.58 (s, 1H, H-aryl), 7.41 (t, 2H, *J* = 7.4 Hz, H-aryl), 7.32 (t, 2H, *J* = 7.3 Hz, H-aryl), 5.27 (d, 1H, $J_{3,4}$ = 3.3 Hz, H-4), 5.11 (t, 1H, $J_{1,NH}$ = 9.5 Hz, H-1), 5.02 (dd, 1H, $J_{3,4}$ = 3.3 Hz, $J_{2,3}$ = 11.2 Hz, H-3), 4.31 (d, 2H, *J* = 7.0 Hz, Fmoc-CH_2), 4.25–4.20 (m, 3H, Fmoc-C*H*, benzene-CH_2), 4.13–3.94 (m, 4H, H-5, H-2, H-6a, H-6b), 3.28 (s, 2H, NHCOCH_2CO), 2.10, 1.98, 1.90, 1.77 (4s, 12H, CH_3); ^{13}C-NMR (DMSO-d_6) δ 170.0, 169.9, 169.6, 167.0, 165.3, 156.3 (8C, C=O), 143.9, 140.8, 140.7, 139.0, 127.6, 127.1, 125.2, 123.0, 121.6, 120.1, 118.6 (11C, C-aryl), 78.9 (C-1), 71.4 (C-5), 70.8 (C-3), 66.7 (C-4), 65.6 (1C, Fmoc-CH$_2$), 61.7 (C-6), 48.2 (C-2), 46.7 (1C, Fmoc-CH), 44.6 (1C, NHCOCH$_2$CO), 43.7 (1C, benzene-CH$_2$), 22.7, 20.6, 20.5, 20.4 (4C, CH$_3$);

FT-ICR-MS: Calcd for $C_{40}H_{42}N_4O_{14}Na$ $[M+Na]^+$: *m/z* 825.258973; found: *m/z* 825.258697.

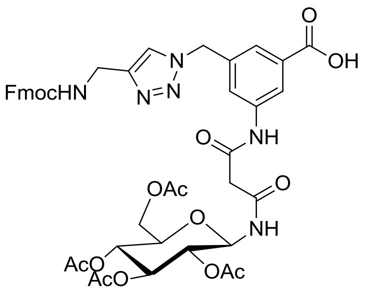

2a

3-{[4-[(9*H*-Fluoren-9-ylmethoxycarbonyl)aminomethyl]-1*H*-1,2,3-triazol-1-yl]methyl}-5-[*N*-(2,3,4,6-tetra-*O*-acetyl-β-D-glucopyranosyl)malonylamino]benzoic acid (2a)

According to General Procedure 114 mg **14a** (0.12 mmol; 1 eq) in 3 ml HCO_2H/DCM (2:1) afforded after column chromatography (PE/acetone 1:1→PE/acetone 2:3 + 1 % HCO_2H) 98 mg (0.11 mmol, 92 %) pure title compound **2a** as white solid. R_f: 0.28 (PE/acetone 2:3 + 1 % HCO_2H); $[\alpha]_D^{20}$: +4.0 (*c* 1.0, MeOH); ^{1}H-NMR (DMSO-d$_6$) δ 10.33 (s, 1H, benzene-N*H*), 8.87 (d, 1H, $J_{1,NH}$ = 9.5 Hz, H-1N*H*COCH$_2$), 7.92 (s, 1H, H-aryl), 7.88 (d, 2H, *J* = 7.5 Hz, H-aryl), 7.83 (t, 1H, *J* = 5.8 Hz, CON*H*CH$_2$), 7.70–7.66 (m, 3H, H-aryl), 7.62 (s, 1H, H-aryl), 7.40 (t, 1H, *J* = 7.5 Hz, H-aryl), 7.29 (t, 1H, *J* = 7.5 Hz, H-aryl), 5.62 (s, 1H, triazole-C*H*$_2$), 5.43–5.33 (m, 2H, H-1, H-3), 4.92–4.81 (m, 2H, H-4, H-2), 4.30 (d, 2H, *J* = 6.9 Hz, Fmoc-C*H*$_2$), 4.24–4.08 (m, 5H, Fmoc-C*H*, benzene-C*H*$_2$, H-6a, H-5), 3.98 (d, 1H, $J_{6a,6b}$ = 10.7 Hz, H-6b), 3.28 (s, 2H, NHCOC*H*$_2$CO), 2.00, 1.99, 1.95, 1.93 (4s, 12H, C*H*$_3$); ^{13}C-NMR (DMSO-d$_6$) δ 172.3, 171.6, 171.5, 171.3, 169.8, 168.7, 167.2, 158.7 (8C, C=O), 147.4, 145.2, 142.6, 140.6, 137.9, 133.4, 128.8, 128.1, 126.2, 125.7, 124.4, 124.3, 122.0, 120.9 (14C, C-aryl), 79.0 (C-1), 74.7 (C-5), 74.6 (C-3), 71.9 (C-2), 69.6 (C-4), 67.8 (1C, Fmoc-*C*H$_2$), 63.1 (C-6), 54.5 (1C, triazole-*C*H$_2$), 48.8 (1C, Fmoc-*C*H), 48.4 (1C, NHCO*C*H$_2$CO),

37.0 (benzene-CH_2), 20.7, 20.6, 20.5 (4C, CH_3); ESI-TOF-MS: Calcd for $C_{43}H_{44}N_6O_{15}Na$ $[M+Na]^+$: *m/z* 907.27569; found: 907.27724.

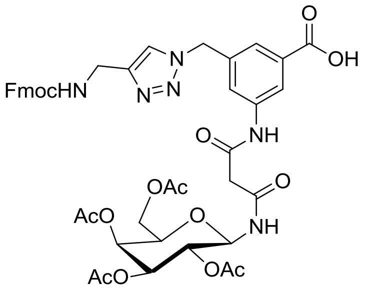

2b

3-{[4-[(9*H*-Fluoren-9-ylmethoxycarbonyl)aminomethyl]-1*H*-1,2,3-triazol-1-yl]methyl}-5-[*N*-(2,3,4,6-tetra-*O*-acetyl-β-D-galactopyranosyl)malonylamino]benzoic acid (2b)

According to General Procedure 88 mg **14b** (0.09 mmol; 1 eq) in 3 ml HCO_2H/DCM (2:1) afforded after column chromatography ($CHCl_3$/MeOH 100:1 + 1 % HCO_2H→50:1 + 1 % HCO_2H) 70 mg (0.08 mmol; 84 %) pure title compound **2b** as white solid. R_f: 0.33 ($CHCl_3$/MeOH 50:1 + 1 % HCO_2H); $[\alpha]_D^{20}$: +20.0 (*c* 1.0, DMSO); ^{1}H-NMR (DMSO-d_6) δ 13.19 (s, 1H, CO_2H), 10.32 (s, 1 H, benzene-N*H*), 8.93 (d, 1H, $J_{1,NH}$ = 9.6 Hz, H-1N*H*COCH_2), 8.19 (s, 1H, H-aryl), 7.92 (s, 1H, H-aryl), 7.89–7.83 (m, 3H, H-aryl, CON*H*CH_2), 7.71–7.62 (m, 3H, H-aryl), 7.62 (s, 1H, H-aryl), 7.40 (t, 2H, *J* = 7.4 Hz, H-aryl), 7.29 (t, 2H, *J* = 7.4 Hz, H-aryl), 5.62 (s, 2H, triazole-CH_2), 5.37 (t, 1H, $J_{1,NH}$ = 9.4 Hz, H-1), 5.31–5.28 (m, 2H, H-3, H-4), 5.06–5.01 (m, 1H, H-2), 4.34–4.29 (m, 3H, H-5, Fmoc-CH_2), 4.24–4.19 (m, 3 H, Fmoc-C*H*, benzene-CH_2), 4.04 (dd, 1H, $J_{6,6a}$ = 5.9 Hz, $J_{6a,6b}$ = 11.4 Hz, H-6a), 3.97 (dd, 1H, $J_{5,6b}$ = 6.8 Hz, $J_{6a,6b}$ = 11.4 Hz, H-6b), 3.31–3.23 (m, 2H, NHCOCH_2CO), 2.11, 1.99, 1.97, 1.91 (4s, 12H, CH_3); ^{13}C-NMR (DMSO-d_6) δ 169.9, 169.8, 169.5, 169.4, 166.9, 166.8, 165.4 (8C, C=O), 145.6, 143.9, 140.7, 139.5, 137.2, 127.6, 127.1, 125.2, 123.7, 122.9, 122.2, 120.1, 119.5 (13C, C-aryl), 77.2 (C-1), 71.4 (C-5), 70.8 (C-3), 68.2 (C-2), 67.6 (C-4),

65.5 (1C, Fmoc-CH_2), 61.5 (C-6), 52.3 (1C, triazole-CH_2), 46.7 (1C, Fmoc-*C*H), 44.6 (1C, NHCOCH_2CO), 36.0 (1C, benzene-CH_2), 20.6, 20.5, 20.4 (4C, CH_3); FT-ICR-MS: Calcd for $C_{43}H_{44}N_6O_{15}Na$ $[M+Na]^+$: *m/z* 907.275686; found: *m/z* 907.275151.

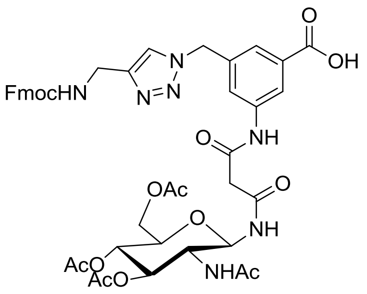

2c

3-{[4-[(9*H*-Fluoren-9-ylmethoxycarbonyl)aminomethyl]-1*H*-1,2,3-triazol-1-yl]methyl}-5-[*N*-(2-Acetamido-2-deoxy-3,4,6-tetra-*O*-acetyl-β-D-glucopyranosyl)malonylamino]benzoic acid (2c)

According to General Procedure 60 mg **14c** (0.06 mmol; 1 eq) in 3 ml HCO_2H/DCM (2:1) afforded after column chromatography ($CHCl_3$/MeOH 30:1→10:1 + 1 % HCO_2H) 52 mg (0.05 mmol; 91 %) pure title compound **2c** as white solid. R_f: 0.24 ($CHCl_3$/MeOH 30:1 + 1 % HCO_2H); $[\alpha]_D^{20}$: +3.0 (*c* 1.0, DMSO); ^{1}H-NMR (DMSO-d_6) δ 13.10 (s, 1H, CO_2H), 10.32 (s, 1 H, benzene-N*H*), 8.72 (d, 1H, $J_{1,NH}$ = 9.1 Hz, H-1N*H*COCH$_2$), 8.21 (s, 1H, H-aryl), 7.97–7.93 (m, 2H, H-aryl, NH), 7.89–7.84 (m, 3 H, H-aryl, CON*H*CH$_2$), 7.71–7.67 (m, 3H, H-aryl), 7.62 (s, 1H, H-aryl), 7.40 (t, 2H, *J* = 7.4 Hz, H-aryl), 7.29 (t, 2H, *J* = 7.4 Hz, H-aryl), 5.63 (s, 2H, triazole-CH_2), 5.17 (t, 1H, $J_{1,NH}$ = 9.5 Hz, H-1), 5.11 (t, 1H, $J_{3,4}$ = 9.8 Hz, H-3), 4.83 (t, 1H, $J_{3,4}$ = 9.8 Hz, H-4), 4.30 (d, 2H, *J* = 6.8 Hz, Fmoc-CH_2), 4.23–4.16 (m, 4H, benzene-CH_2, Fmoc-C*H*, H-6a), 3.97–3.90 (m, 2H, H-6b, H-2), 3.86–3.83 (m, 1H, H-5), 3.28 (s, 2H, NHCOCH_2CO), 2.00, 1.97, 1.91, 1.74 (4s, 12H, CH_3); ^{13}C-NMR (DMSO-d_6) δ 170.1, 169.6, 169.5, 169.3, 167.0, 166.8, 165.4, 156.2 (8C, C=O), 145.6, 143.9, 140.7, 139.5, 137.3, 131.8, 127.6, 127.1, 125.2, 123.7, 122.9, 122.4, 120.1, 119.5 (14C, C-

aryl), 78.1 (C-1), 73.3 (C-3), 72.3 (C-5), 68.3 (C-4), 65.5 (1C, Fmoc-*C*H$_2$), 61.8 (C-6), 52.3 (1C, triazole-*C*H$_2$), 52.0 (C-2), 46.7 (1C, Fmoc-*C*H), 44.6 (1C, NHCO*C*H$_2$CO), 36.0 (1C, benzene-*C*H$_2$), 22.6, 20.6, 20.4, 20.3 (4C, *C*H$_3$); FT-ICR-MS: Calcd for $C_{43}H_{45}N_7O_{14}Na$ [M+Na]$^+$: *m/z* 906.291670; found: *m/z* 906.291523.

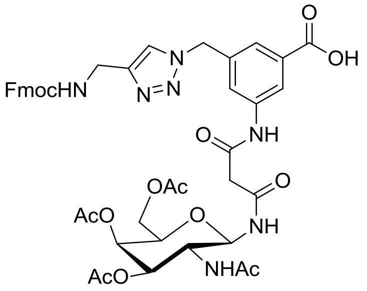

2d

3-{[4-[(9*H*-Fluoren-9-ylmethoxycarbonyl)aminomethyl]-1*H*-1,2,3-triazol-1-yl]methyl}-5-[*N*-(2-Acetamido-2-deoxy-3,4,6-tetra-*O*-acetyl-β-D-galactopyranosyl)malonylamino]benzoic acid (2d)

According to General Procedure 107 mg **14d** (0.11 mmol; 1 eq) in 3 ml HCO_2H/DCM (2:1) afforded after column chromatography ($CHCl_3$/MeOH 30:1→10:1 + 1 % HCO_2H) 94 mg (0.10 mmol; 94 %) pure title compound **2d** as white solid. R_f: 0.16 ($CHCl_3$/MeOH 30:1 + 1 % HCO_2H);$[\alpha]_D^{20}$: +12.2 (*c* 1.0, DMSO); ^{1}H-NMR (DMSO-d$_6$) δ 13.31 (s, 1H, CO$_2$*H*), 10.29 (s, 1H, benzene-N*H*), 8.66 (d, 1H, $J_{1,NH}$ = 9.2 Hz, H-1N*H*COCH$_2$), 8.18 (s, 1H, H-aryl), 7.95–7.92 (m, 2H, CON*H*CH$_2$, H-aryl), 7.88–7.83 (m, 3H, N*H*, H-aryl), 7.71–7.67 (m, 3H, H-aryl), 7.62 (s, 1H, H-aryl), 7.40 (t, 2H, *J* = 7.4 Hz, H-aryl), 7.29 (t, 2H, *J* = 7.4 Hz, H-aryl), 5.61 (s, 2H, triazole-C*H*$_2$), 5.27 (d, 1H, $J_{3,4}$ = 3.3 Hz, H-4), 5.10 (t, 1H, $J_{1,NH}$ = 9.2 Hz, H-1), 5.01 (dd, 1H, $J_{3,4}$ = 3.3 Hz, $J_{2,3}$ = 11.2 Hz, H-3), 4.30 (d, 2H, *J* = 7.0 Hz, Fmoc-C*H*$_2$), 4.24–4.19 (m, 3H, benzene-C*H*$_2$, Fmoc-C*H*), 4.12–3.94 (m, 4H, H-5, H-2, H-6a, H-6b), 3.27 (s, 2H, NHCOC*H*$_2$CO), 2.09, 1.98, 1.90, 1.76 (4s, 12H, C*H*$_3$); ^{13}C-NMR (DMSO-d$_6$) δ 170.0, 169.9, 169.6, 166.9, 165.4, 156.2 (8C, C=O), 145.6, 143.9, 140.7, 139.4, 137.0, 127.6, 127.1,

125.2, 123.7, 122.9, 121.9, 120.1, 119.6 (13C, C-aryl), 78.9 (C-1), 71.4 (C-5), 70.8 (C-3), 66.7 (C-4), 65.5 (1C, Fmoc-CH_2), 61.7 (C-6), 52.4 (1C, triazole-CH_2), 48.2 (C-2), 46.7 (1C, Fmoc-*C*H), 44.6 (1C, NHCOCH_2CO), 36.0 (1C, benzene-CH_2), 22.7, 20.6, 20.5, 20.4 (4C, CH_3); FT-ICR-MS: Calcd for $C_{43}H_{45}N_7O_{14}Na$ $[M+Na]^+$: *m/z* 906.291670; found: *m/z* 906.292359.

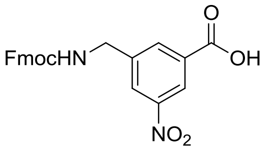

15

3-[(9*H*-Fluoren-9-ylmethoxycarbonyl)aminomethyl]-5-nitrobenzoic acid (15)

According to General Procedure 120 mg **6** (0.25 mmol; 1 eq) in 6 ml HCO_2H/DCM (2:1) afforded after column chromatography ($CHCl_3$/MeOH 50:1→25:1 + 1 % HCO_2H) 99 mg (0.24 mmol; 93 %) pure title compound **15** as white solid. R_f: 0.42 ($CHCl_3$/MeOH 25:1 + 1 % HCO_2H); ^{1}H-NMR (DMSO-d_6) δ 13.72 (s, 1H, CO_2H), 8.50 (s, 1H, H-aryl), 8.35 (s, 1H, H-aryl), 8.27 (s, 1H, H-aryl), 8.10 (t, 1H, J = 6.1 Hz, CON*H*CH_2), 7.88 (d, 2H, J = 7.5 Hz, H-aryl), 7.69 (d, 2H, J = 7.4 Hz, H-aryl), 7.40 (t, 2H, J = 7.4 Hz, H-aryl), 7.31 (t, 2H, J = 7.4 Hz, H-aryl), 4.39–4.35 (m, 4H, Fmoc-CH_2, benzene-CH_2), 4.24 (t, 1H, J = 6.7 Hz, Fmoc-C*H*); ^{13}C-NMR (DMSO-d_6) δ 165.5, 156.4 (2C, C=O), 148.0, 143.1, 140.8, 133.8, 132.6, 127.1, 125.6, 125.1, 122.3, 120.2 (10C, C-aryl), 65.6 (1C, Fmoc-CH_2), 46.7 (1C, Fmoc-*C*H), 43.0 (1C, benzene-CH_2); FT-ICR-MS: Calcd for $C_{23}H_{18}N_2O_6Na$ $[M+Na]^+$: *m/z* 441.105707; found *m/z* 441.105876.

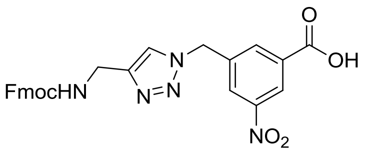

18

3-{[4-[(9*H*-Fluoren-9-ylmethoxycarbonyl)aminomethyl]-1*H*-1,2,3-triazol-1-yl]methyl}-5-nitrobenzoic acid (18)

According to General Procedure 308 mg **8** (0.55 mmol; 1 eq) in 18 ml HCO_2H/DCM (2:1) afforded after column chromatography ($CHCl_3$/MeOH 50:1→25:1 + 1 % HCO_2H) 258 mg (0.52 mmol; 93 %) pure title compound **18** as white solid. R_f: 0.63 ($CHCl_3$/MeOH 25:1 + 1 % HCO_2H); ^{1}H-NMR (DMSO-d_6) δ 13.86 (s, 1H, CO_2H), 8.56 (s, 1H, H-aryl), 8.46 (s, 1H, H-aryl), 8.31 (s, 1H, H-aryl), 8.08 (s, 1H, H-aryl), 7.88–7.85 (m, 3H, H-aryl, CON*H*CH_2), 7.67 (d, 2H, *J* = 7.4 Hz, H-aryl), 7.40 (t, 2H, *J* = 7.4 Hz, H-aryl), 7.29 (t, 2H, *J* = 7.4 Hz, H-aryl), 5.83 (s, 2H, triazole-CH_2), 4.30 (d, 2H, *J* = 7.0 Hz, Fmoc-CH_2), 4.25 (d, 2H, *J* = 5.8 Hz, benzene-CH_2), 4.20 (t, 1H, *J* = 6.9 Hz, Fmoc-C*H*); ^{13}C-NMR (DMSO-d_6) δ 165.2, 156.2 (2C, C=O), 148.1, 145.7, 143.8, 140.7, 138.9, 134.9, 133.1, 127.6, 127.0, 126.8, 125.2, 123.5, 123.3, 120.1 (14C, C-aryl), 65.5 (1C, Fmoc-*C*H_2), 51.3 (1C, triazole-*C*H_2), 46.7 (1C, Fmoc-*C*H), 35.9 (1C, benzene-*C*H_2); FT-ICR-MS: Calcd for $C_{26}H_{21}N_5O_6Na$ $[M+Na]^+$: *m/z* 522.138405; found: *m/z* 522.138737; Anal. Calcd for $C_{26}H_{21}N_5O_6$(499.47): C 62.52; H 4.24; N 14.02; found: C 62.78; H 4.12; N 14.01.

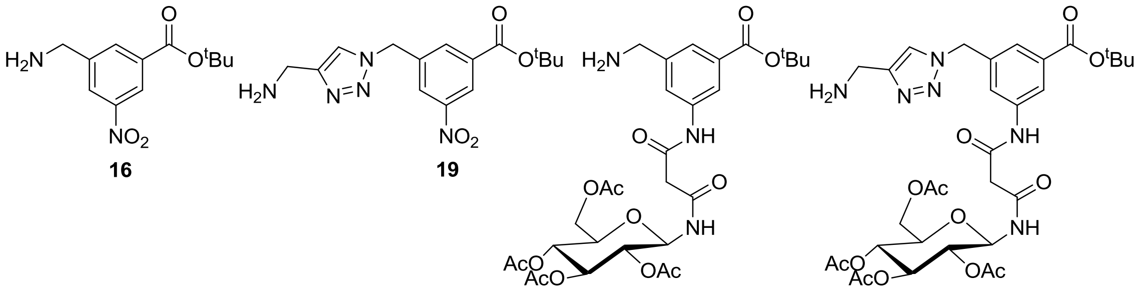

16, 19, 21, 23

General Procedure for synthesis of compounds **16**, **19**, **21 and 23**

The Fmoc-protected amine **6**, **8**, **13a** or **14a** (1 eq) was stirred in 2 ml 20 % piperidine/DMF at r.t. for 3 ½ h until TLC indicated complete consumption of the starting material. The solvent was removed under reduced pressure and the residue was co-evaporated with toluene (5 x 20 ml) to afford crude title compound **16**, **19**, **21** and **23** which was used without further purification.

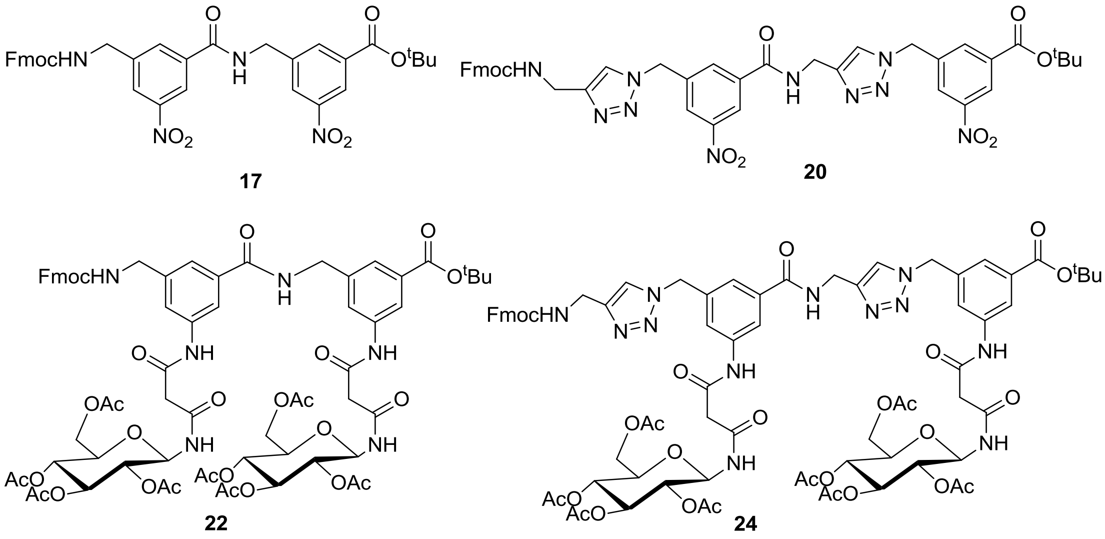

17, 20, 22, 24

General Procedure for synthesis of compounds 17, 20, 22 and 24.

In a 25 ml round bottom flask equipped with a gas inlet and a stirring bar **15, 18, 1a** or **2a** (1 eq) was dissolved in 12 ml dry DMF under an atmosphere of nitrogen. The solution was cooled to 0°C and HBTU (1.3 eq), HOBt (1.3 eq) and DIPEA (3.9 eq) were added. The mixture was stirred at 0°C for 10 min. Afterwards **16**, **19**, **21** or **23** (1

eq) was added and the resulting solution was stirred at 0°c for 2 h. Thereafter the solution was stirred at r.t. for 13 h. The solvent was removed under reduced pressure, the residue was dissolved in ethyl acetate (70 ml) and transferred into a separatory funnel. The organic layer was acidified with 10 % citric acid-soln. (2 x 20 ml), washed with sat. $NaHCO_3$-soln. (3 x 20 ml), sat. NaCl-soln. (20 ml), dried over Na_2SO_4 and the solvent was evaporated. Purification of the residue via column chromatography afforded pure title compound **17, 20, 22** or **24** as white solid.

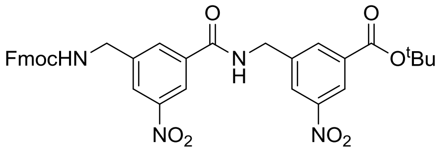

17

3-{[3-[(9*H*-Fluoren-9-ylmethoxycarbonyl)aminomethyl]-5-nitrobenzoyl]aminomethyl}-5-nitrobenzoic acid *tert*-butyl ester (17)

According to General Procedure 96 mg **15** (0.23 mmol; 1 eq) afforded after column chromatography (PE/EA 1:1) 110 mg (0.17 mmol; 73 %) pure title compound **17** as white solid. R_f: 0.37 (PE/EA 1:1); Mp 131.1 °C (*n*-hexane/$CHCl_3$); ^{1}H-NMR (DMSO-d_6) δ 9.64 (t, 1H, *J* = 5.9 Hz, N*H*), 8.65 (s, 1H, H-aryl), 8.47–8.45 (m, 2H, H-aryl), 8.29–8.25 (m, 3H, H-aryl), 8.08 (t, 1H, *J* = 6.1 Hz, N*H*), 7.88 (d, 2H, *J* = 7.5 Hz, H-aryl), 7.68 (d, 2H, *J* = 7.4 Hz, H-aryl), 7.39 (t, 2H, *J* = 7.4 Hz, H-aryl), 7.29 (t, 2H, *J* = 7.3 Hz, H-aryl), 4.69 (d, 2H, *J* = 5.7 Hz, benzene'-CH_2), 4.37–4.34 (m, 4H, Fmoc-CH_2, benzene-CH_2), 4.23 (t, 1H, *J* = 6.7 Hz, Fmoc-C*H*), 1.56 (s, 9H, $CO_2C(CH_3)_3$); ^{13}C-NMR (DMSO-d_6) δ 164.4, 163.0, 156.4 (3C, C=O), 148.0, 147.9, 143.8, 142.8, 142.4, 140.7, 135.2, 134.0, 132.8, 132.6, 128.9, 127.6, 127.3, 127.0, 126.3, 125.1, 124.5, 122.1, 121.4, 120.4, 120.1, 120.0 (22C, C-aryl), 82.3 (1C, $CO_2C(CH_3)_3$), 65.6 (1C, Fmoc-*C*H_2), 46.7 (1C, Fmoc-*C*H), 43.2 (1C, benzene'-*C*H_2), 42.2 (1C, benzene-

CH_2), 27.6 (3C, $CO_2C(CH_3)_3$); FT-ICR-MS: Calcd for $C_{35}H_{32}N_4O_9Na$ $[M+Na]^+$: *m/z* 675.206150; found: *m/z* 675.206367; Anal. Calcd for $C_{35}H_{32}N_4O_9$(652.65): C 64.41; H 4.94; N 8.58; found: C 64.76; H 4.94; N 8.29.

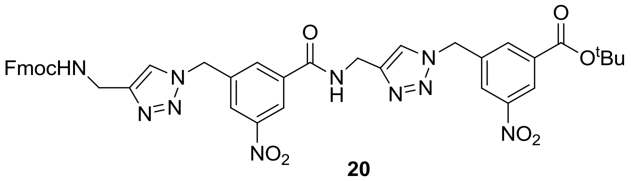

20

3-{3-[[4-[(9*H*-Fluoren-9-ylmethoxycarbonyl)aminomethyl]-1*H*-1,2,3-triazol-1-yl]methyl]-5-nitrobenzoyl]aminomethyl-1*H*-1,2,3-triazol-1-yl]methyl}-5-nitrobenzoic acid *tert*-butyl ester (20)

According to General Procedure 168 mg **18** (0.34 mmol; 1 eq) afforded after column chromatography ($CHCl_3$/MeOH 50:1→25:1) 241 mg (0.30 mmol; 88 %) pure title compound **20** as pale yellow solid. R_f: 0.27 ($CHCl_3$/MeOH 25:1); Mp 128.3 °C ($Et_2O/CHCl_3$);^{1}H-NMR ($CDCl_3$) δ 8.87 (s, 1H, N*H*), 8.64 (s, 1H, H-aryl), 8.47 (s, 1H, H-aryl), 8.22 (d, 2H, *J* = 7.7 Hz, H-aryl), 8.09 (d, 2H, *J* = 12.2 Hz, H-aryl), 7.82 (s, 1H, H-aryl), 7.67 (d, 2H, *J* = 7.5 Hz, H-aryl), 7.59 (s, 1H, H-aryl), 7.48 (d, 2H, *J* = 7.4 Hz, H-aryl), 7.31 (t, 2H, *J* = 7.4 Hz, H-aryl), 7.18 (t, 2H, *J* = 7.4 Hz, H-aryl), 6.22 (s, 1H, N*H*), 5.64 (s, 2H, benzene-CH_2), 5.52 (s, 2H, benzene'-CH_2), 4.62 (d, 2H, *J* = 5.6 Hz, triazole-CH_2), 4.39 (d, 2H, *J* = 4.2 Hz, triazole'-CH_2), 4.29 (d, 2H, *J* = 6.9 Hz, Fmoc-CH_2), 4.08 (t, 1H, *J* = 6.9 Hz, Fmoc-C*H*), 1.58 (s, 9H, $CO_2C(CH_3)_3$); ^{13}C-NMR ($CDCl_3$) δ 164.6, 162.9, 156.7 (3C, C=O), 148.7, 148.4, 146.1, 145.3, 143.9, 141.3, 137.2, 136.9, 136.4, 134.7, 133.0, 127.8, 127.1, 126.5, 125.5, 125.1, 124.6, 123.9, 122.9, 122.7, 120.1 (21C, C-aryl), 80.4 (1C, $CO_2C(CH_3)_3$), 66.9 (1C, Fmoc-*C*H_2), 53.0 (1C, benzene-*C*H_2), 52.8 (1C, benzene'-*C*H_2), 47.2 (1C, Fmoc-*C*H), 36.4 (1C, triazole-*C*H_2), 35.3 (1C, triazole'-*C*H_2), 28.2 (3C, $CO_2C(CH_3)_3$); FT-ICR-MS: Calcd for $C_{41}H_{38}N_{10}O_9Na$ $[M+Na]^+$: *m/z* 837.271544; found *m/z* 837.270940.

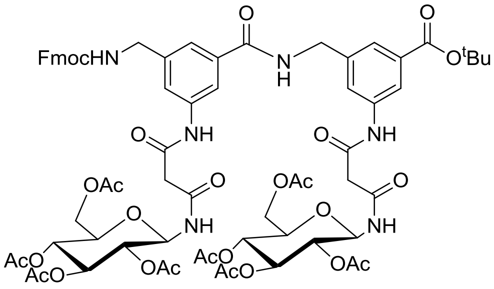

22

3-{[3-[(9*H*-Fluoren-9-ylmethoxycarbonyl)aminomethyl]-5-[*N*-(2,3,4,6-tetra-*O*-acetyl-β-D-glucopyranosyl)malonylamino]benzoyl]aminomethyl}-5-[*N*-(2,3,4,6-tetra-*O*-acetyl-β-D-glucopyranosyl)malonylamino]benzoic acid *tert*-butyl ester (22)

According to General Procedure 95 mg **1a** (0.12 mmol; 1 eq) afforded after column chromatography ($CHCl_3$/MeOH 50:1→25:1) 86 mg (0.06 mmol; 51 %) pure title compound **22** as white solid. R_f: 0.35 ($CHCl_3$/MeOH 25:1); $[\alpha]_D^{20}$: -9.0 (*c* 1.0, $CHCl_3$); ^{1}H-NMR ($CDCl_3$) δ 9.51 (s, 1H, N*H*), 9.30 (s, 1H, N*H*), 8.09-7.95 (m, 4H, N*H*, H-aryl), 7.72-7.70 (m, 2H, H-aryl), 7.57-7.55 (m, 4H, N*H*, H-aryl), 7.39-7.32 (m, 5H, H-aryl), 7.25-7.21 (m, 2H, H-aryl), 6.36 (s, 1H, N*H*), 5.34-5.25 (m, 4H, H-1, H-1', H-3, H-3'), 5.06-4.97 (m, 4H, H-4, H-4', H-2, H-2'), 4.49 (s, 2H, benzene'-CH_2), 4.35 (d, 2H, *J* = 6.8 Hz, Fmoc-CH_2), 4.19-4.17 (m, 5H, H-6a, H-6a', Fmoc-C*H*, benzene-CH_2), 4.03-4.00 (m, 2H, H-6b, H6b'), 3.83-3.81 (m, 2H, H-5, H-5'), 3.39-3.35 (m, 4H, NHCOCH_2CO), 2.01, 2.00, 1.98, 1.97, 1.95, 1.93 (6s, 24H, CH_3), 1.55 (s, 9H, $CO_2C(CH_3)_3$); ^{13}C-NMR ($CDCl_3$) δ 170.8, 170.7, 170.6, 170.1, 170.0, 169.6, 168.7, 168.5, 167.6, 165.6, 165.3, 165.2, 157.3 (15C, C=O), 144.0, 141.3, 140.2, 139.5, 138.3, 138.2, 132.8, 127.8, 127.2, 125.3, 124.0, 122.0, 121.8, 121.4, 120.1, 119.5, 117.0 (17C, C-aryl), 81.7 (1C, $CO_2C(CH_3)_3$), 78.2, 78.1 (2C, C-1, C-1'), 73.8, 73.7 (2C, C-5, C-5'), 73.2, 73.1 (2C, C-3, C-3'), 70.6, 70.4 (2C, C-4, C-4'), 68.2, 68.1 (2C, C-2, C-2'), 67.2 (1C, Fmoc-CH_2), 61.8, 61.7 (2C, C-6, C-6'), 47.1 (1C, Fmoc-*C*H),

44.6 (1C, benzene-*C*H_2), 44.1 (1C, benzene'-*C*H_2), 43.6 (1C, NHCO*C*H_2CO), 28.1 (3C, $CO_2C(CH_3)_3$), 20.7, 20.6 (8C, *C*H_3); ESI-TOF-MS: Calcd for $C_{69}H_{78}N_6O_{27}Na$ $[M+Na]^+$: *m/z* 1445.48071*;* found: *m/z* 1445.48129*.*

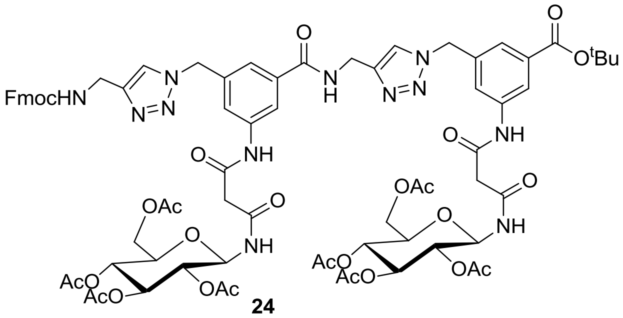

24

3-{3-[[4-[(9*H*-Fluoren-9-ylmethoxycarbonyl)aminomethyl]-1*H*-1,2,3-triazol-1-yl]methyl]-5-[*N*-(2,3,4,6-tetra-*O*-acetyl-β-D-glucopyranosyl)malonylamino]benzoyl]aminomethyl-1*H*-1,2,3-triazol-1-yl]methyl}-5-[*N*-(2,3,4,6-tetra-*O*-acetyl-β-D-glucopyranosyl)malonylamino]benzoic acid *tert*-butyl ester (24)

According to General Procedure 108 mg **2a** (0.12 mmol; 1 eq) afforded after column chromatography ($CHCl_3$/MeOH 50:1→25:1) 106 mg (0.07 mmol; 55 %) pure title compound **24** as white solid. R_f: 0.29 ($CHCl_3$/MeOH 25:1); $[\alpha]_D^{20}$: +6.0 (*c* 1.0, DMSO); ^{1}H-NMR (DMSO-d_6) δ 10.40-10.30 (m, 2H, N*H*), 9.07-9.06 (m, 1H, N*H*), 8.90-8.70 (m, 2H, N*H*), 8.16-8.14 (m, 1H, H-aryl), 8.05-8.02 (m, 2H, H-aryl), 7.91-7.81 (m, 4H, H-aryl, N*H*), 7.69-7.67 (m, 3H, H-aryl), 7.57 (s, 3H, H-aryl), 7.40 (t, 2H, *J* = 7.3 Hz, H-aryl), 7.29 (t, 2H, *J* = 7.3 Hz, H-aryl), 5.62 (s, 2H, triazole-C*H_2*), 5.58 (s, 2H, triazole'-C*H_2*), 5.44-4.75 (m, 8H, H-1, H-1', H-3, H-3', H-4, H-4', H-2, H-2'), 4.50-4.49 (m, 2H, benzene-C*H_2*), 4.31-3.88 (m, 11H, Fmoc-C*H_2*, benzene'-C*H_2*, Fmoc-C*H*, H-6a, H-6a', H-5, H-5', H-6b, H-6b'), 3.31-3.22 (m, 4H, NHCOC*H_2*CO), 2.08, 2.02, 2.01, 1.99, 1.97, 1.95, 1.94 (7s, 24H, C*H_3*), 1.52 (s, 9H, $CO_2C(CH_3)_3$); ^{13}C-NMR (DMSO-d_6) δ 170.4, 170.1, 170.0, 169.6, 169.3, 169.2, 167.2, 167.1, 167.0, 166.9, 165.8, 165.4,

165.3, 164.4, 156.2 (15C, C=O), 145.6, 145.2, 143.9, 140.8, 139.6, 139.4, 137.3, 136.8, 135.5, 132.3, 127.7, 127.1, 125.2, 123.4, 122.8, 122.0, 121.4, 120.1, 119.2, 118.2 (20C, C-aryl), 81.1 (1C, $CO_2\mathit{C}(CH_3)_3$), 77.1, 76.9 (2C, C-1, C-1'), 72.8, 72.6 (2C, C-3, C-3'), 72.5, 72.2 (2C, C-5, C-5'), 70.6, 69.8 (2C, C-2, C-2'), 67.9 (2C, C-4, C-4'), 65.6 (1C, Fmoc-$\mathit{C}H_2$), 61.8 (2C, C-6, C-6'), 52.7, 52.3 (2C, benzene-$\mathit{C}H_2$, benzene'-$\mathit{C}H_2$), 46.7 (1C, Fmoc-*C*H), 44.8, 44.7 (2C, triazole-$\mathit{C}H_2$, triazole'-$\mathit{C}H_2$), 27.8 (1C, $CO_2C(\mathit{C}H_3)_3$), 20.9, 20.8, 20.7, 20.6, 20.5, 20.4, 20.3 (8C, $\mathit{C}H_3$); ESI-TOF-MS: Calcd for $C_{75}H_{84}N_{12}O_{27}Na$ $[M+Na]^+$: *m/z* 1607.54611; found: *m/z* 1607.54480.

References

1. Harris, T. D.; Rajopadhyne, M.; Damphousse, P. J.; Glowacka, D.; Yu, K.; Bourque, J. P.; Barrett, J. A.; Damphousse, D. J.; Heminway, S. J.; Lazewatsky, J.; Mazaika, T.; Carroll, T. R. *Bioorg. Med. Lett.*, **1996**, *6*, 1741-1746.

2. Watzke, A.; Gutierrez-Rodriguez M.; Köhn, M.; Wacker, R.; Schroeder, H.; Niemeyer, C. M.; Goody, R. S.; Waldmann, H.; *Bioorg. Med. Chem.*, **2006**, *14*, 6288-6306.

3. Pokorski, J. K.; *Org. Lett.*, **2007**, *9*, 2381-2383

4. Helferich, B.; Mitrowsky, A.; *Chem. Ber.* **1952**, *85*, 1-94.

5. Badia, C.; Souard, F.; Vicent, C.; *J. Org. Chem.* **2012**, *77*, 10870-10881

6. Tanaka, M.; Yamashina, I.; *Carbohydr. Res.* **1973**, *27*, 175-183.

7. Neumann, J.; Thiem, J.; *Eur. J. Org. Chem*. **2010**, 900-908.

8. Dunstan, D.; Hough, L.; *Carbohydr. Res.* **1972**, *25*, 246-248.

9. Fujita, Y.; Abu-Baker, M. A.; Wimmer, N.; Batzloff, M. R.; Good, M. F.; Toth, I.; *Bioorg. Med. Chem*. **2008**, *16*, 8907-8913.
