# Supplementary material for: Synthesis of aromatic glycoconjugates. Building blocks for the construction of combinatorial glycopeptide libraries
Source: Beilstein J Org Chem. 2014 Oct 22;10:2453–60. doi: 10.3762/bjoc.10.256 (PMC4222372; doi:10.3762/bjoc.10.256)

Supporting Information

for

Synthesis of aromatic glycoconjugates. Building blocks for the construction of combinatorial glycopeptide libraries

Markus Nörrlinger, Thomas Ziegler*

Address: Institute of Organic Chemistry, University of Tuebingen, Auf der Morgenstelle 18, 72076 Tuebingen, Germany

Email: Thomas Ziegler - thomas.ziegler@uni-tuebingen.de

* Corresponding author

NMR Spectra

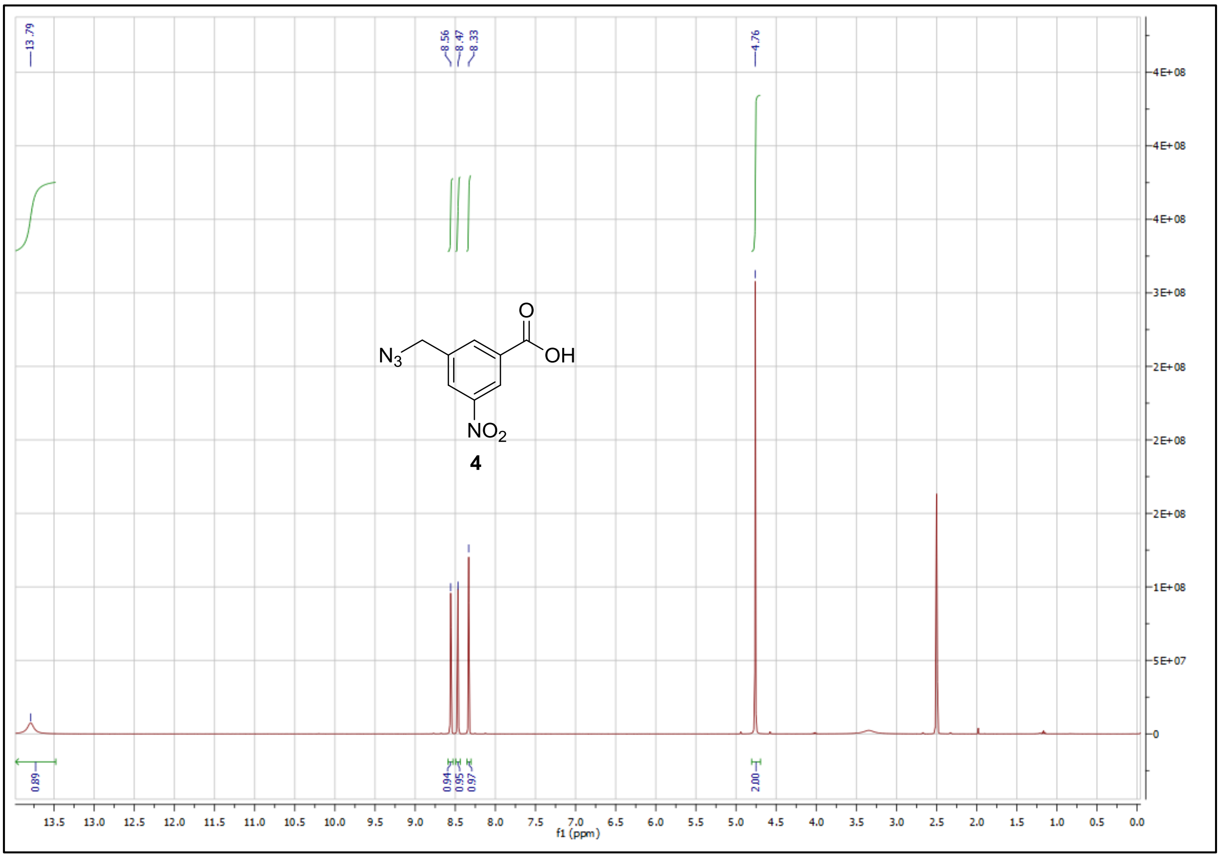
13.79
8.56
8.47
8.33
4.76
N3
O
OH
NO2
4
0.89
0.94
0.95
0.97
2.00
f1 (ppm)

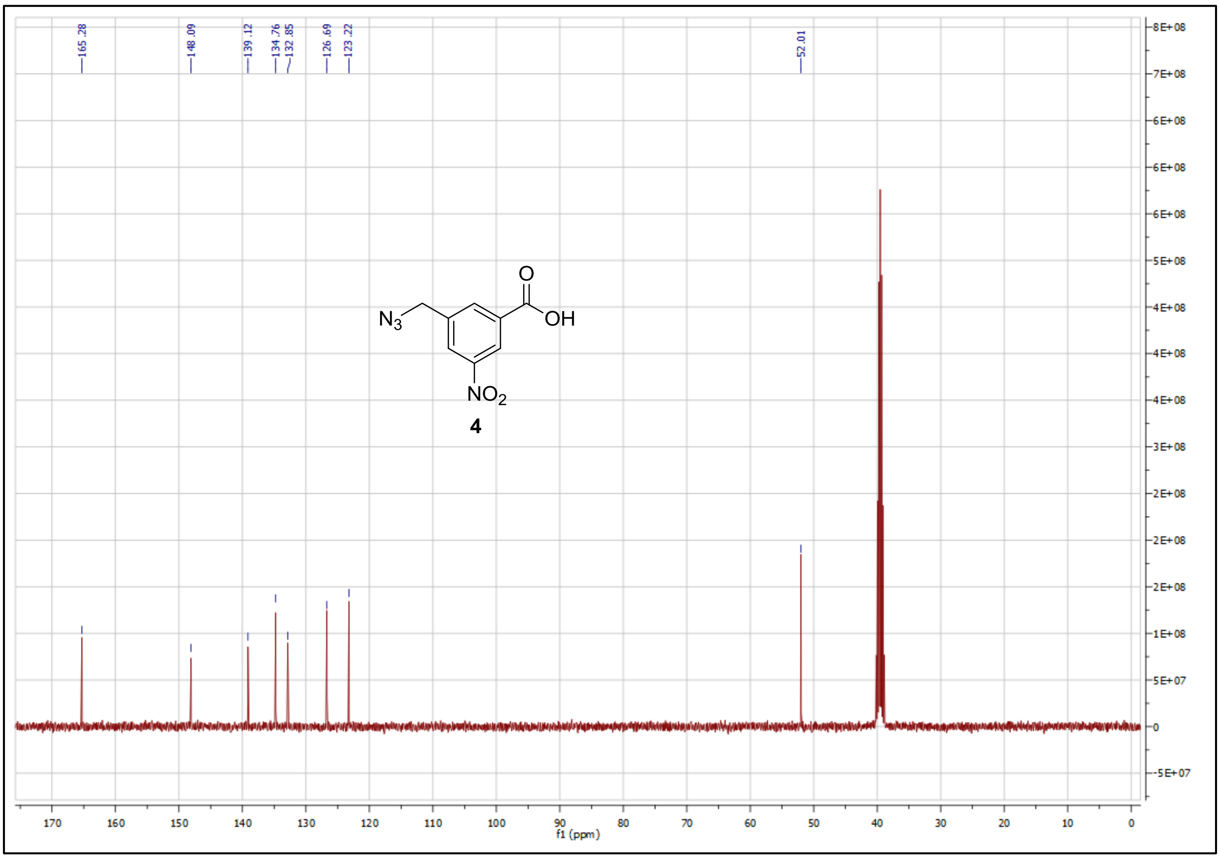
165.28
148.09
139.12
134.76
132.85
126.69
123.22
52.01
N3
O
OH
NO2
4
f1 (ppm)

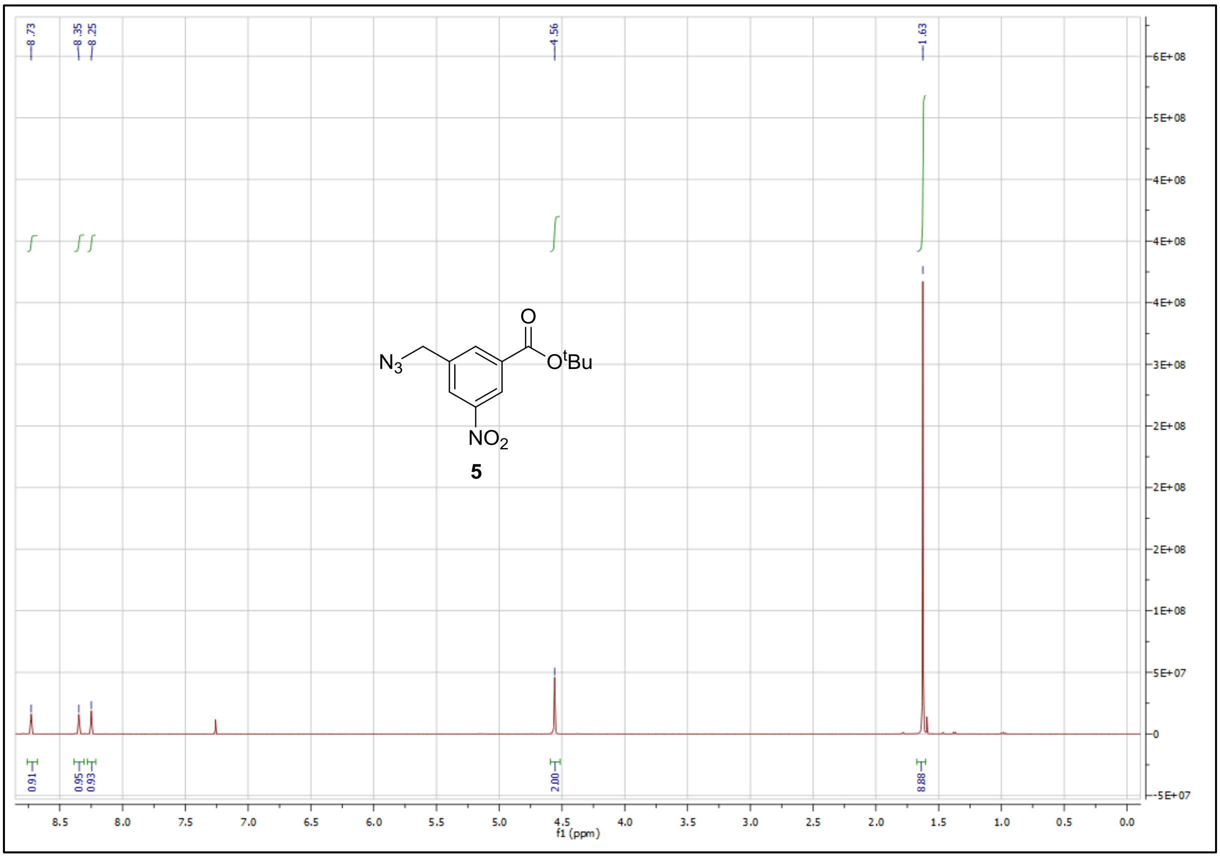
8.73
8.35
8.25
4.56
1.63
N3
O
OtBu
NO2
5
0.91
0.95
0.93
2.00
8.88
f1 (ppm)

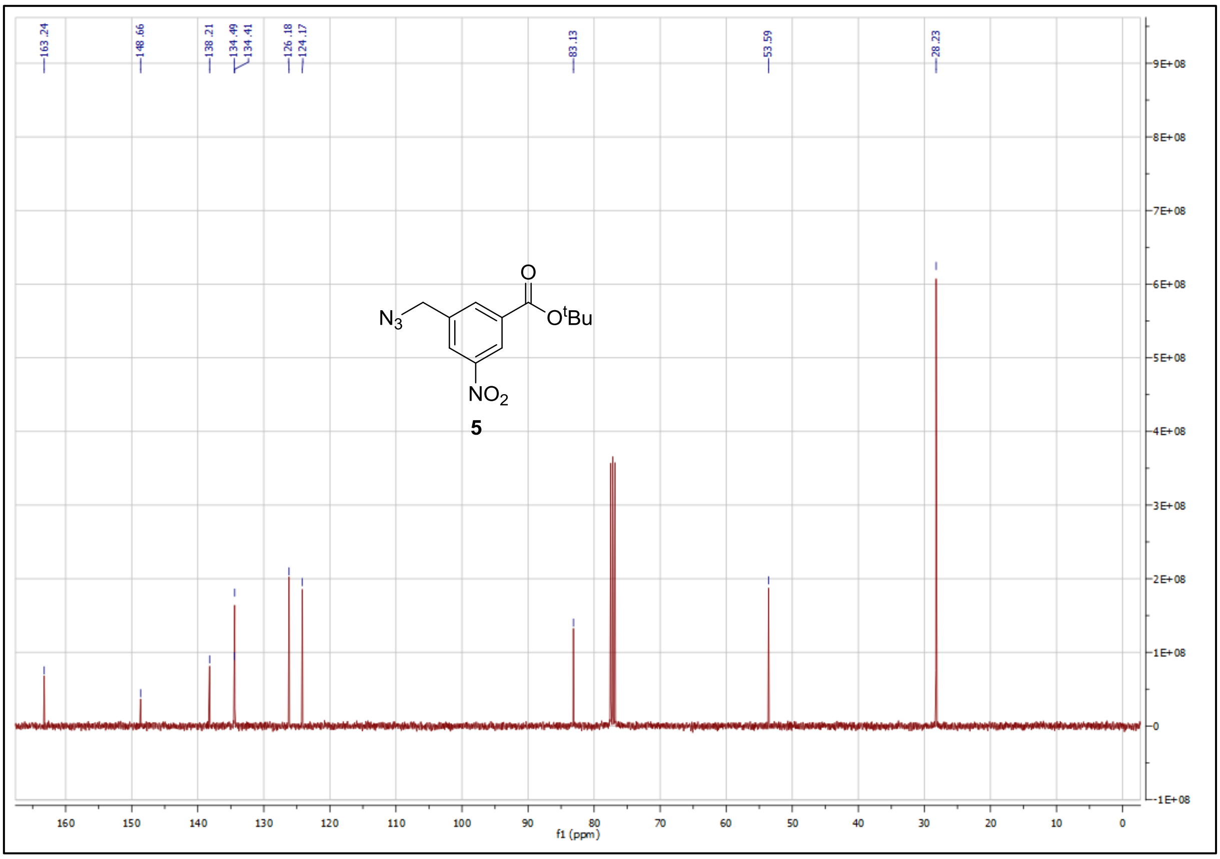
163.24
148.66
138.21
134.49
134.41
126.18
124.17
83.13
53.59
28.23
N3
O
OtBu
NO2
5
f1 (ppm)

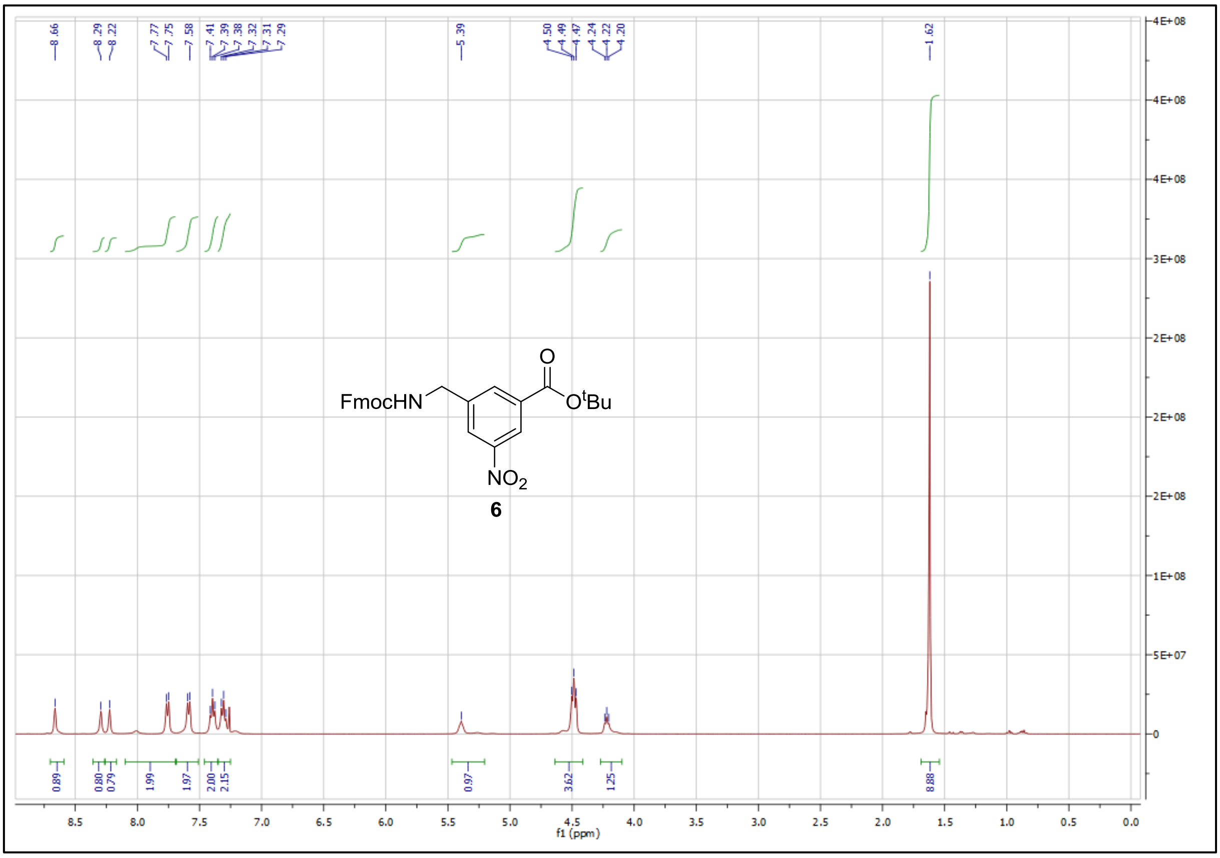
8.66
8.29
8.22
7.77
7.75
7.58
7.41
7.39
7.38
7.32
7.31
7.29
5.39
4.50
4.49
4.47
4.24
4.22
4.20
1.62
FmocHN
O
O^{t}Bu
NO_2
6
0.89
0.80
0.79
1.99
1.97
2.00
2.15
0.97
3.62
1.25
8.88
f1 (ppm)

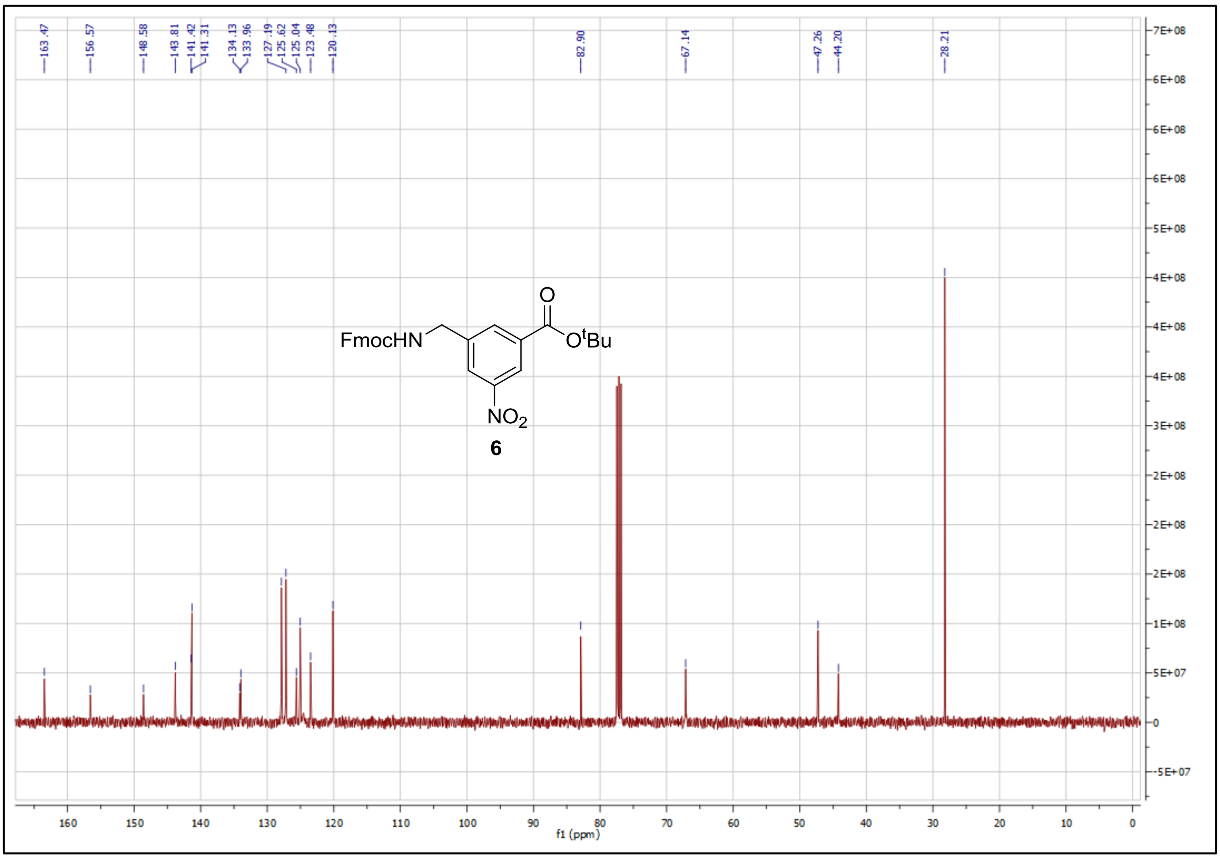
163.47
156.57
148.58
143.81
141.42
141.31
134.13
133.96
127.19
125.62
125.04
123.48
120.13
82.90
67.14
47.26
44.20
28.21
FmocHN
O
O^{t}Bu
NO_2
6
f1 (ppm)

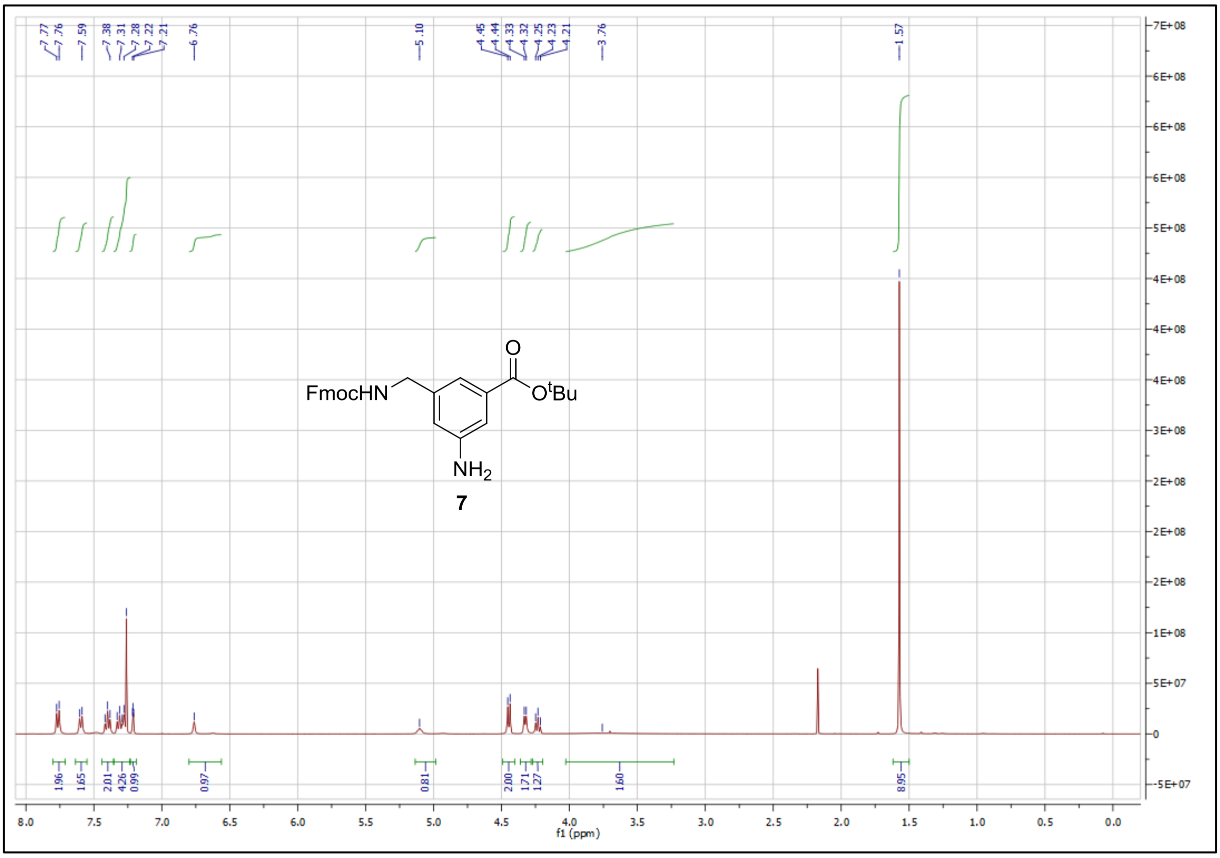

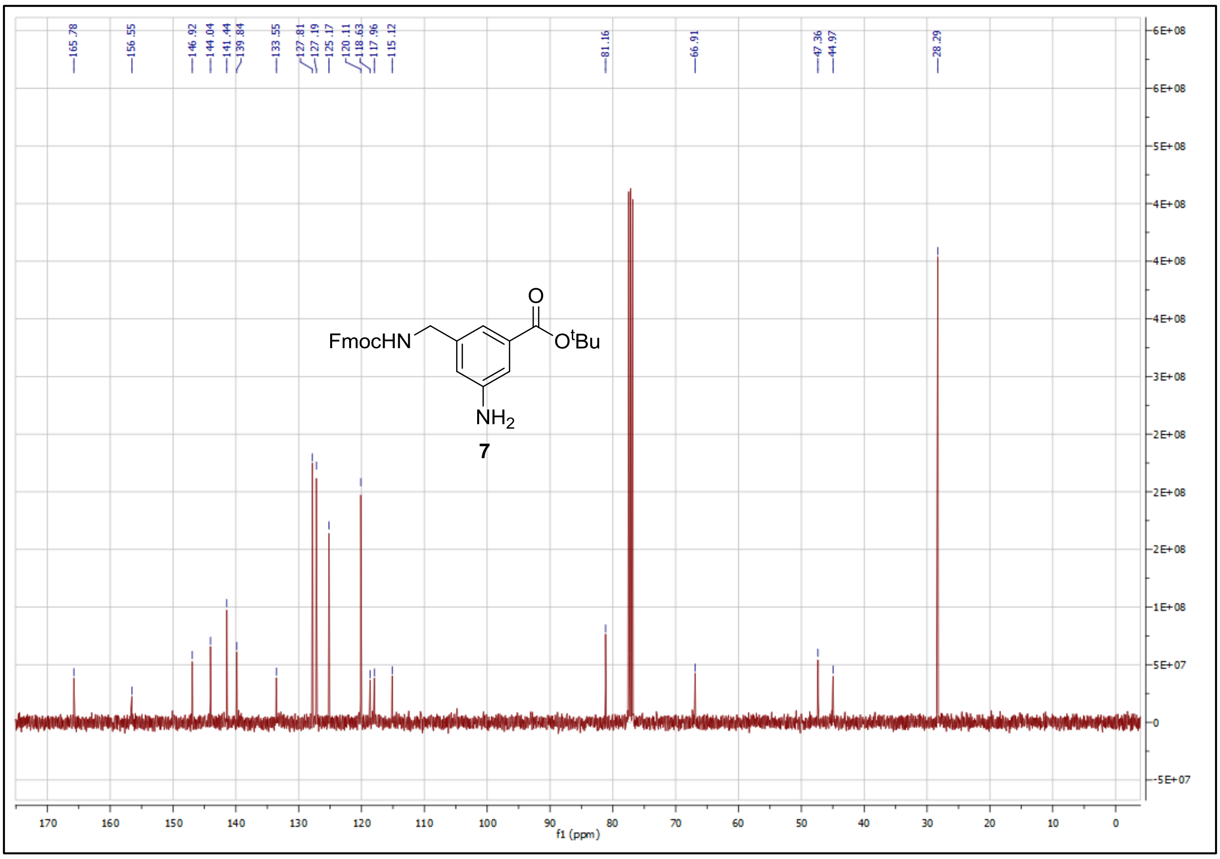

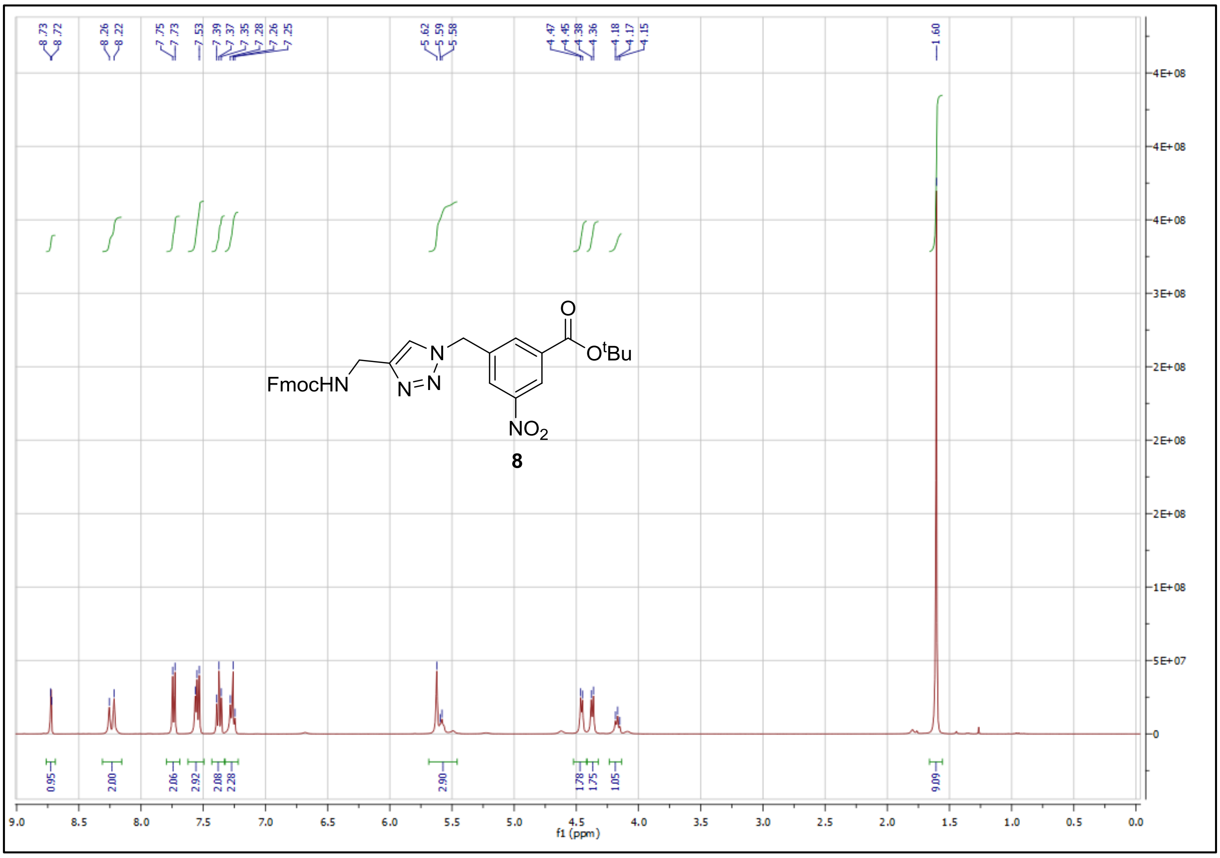

FmocHN
N
N=N
O
O^{t}Bu
NO$_2$
8
f1 (ppm)

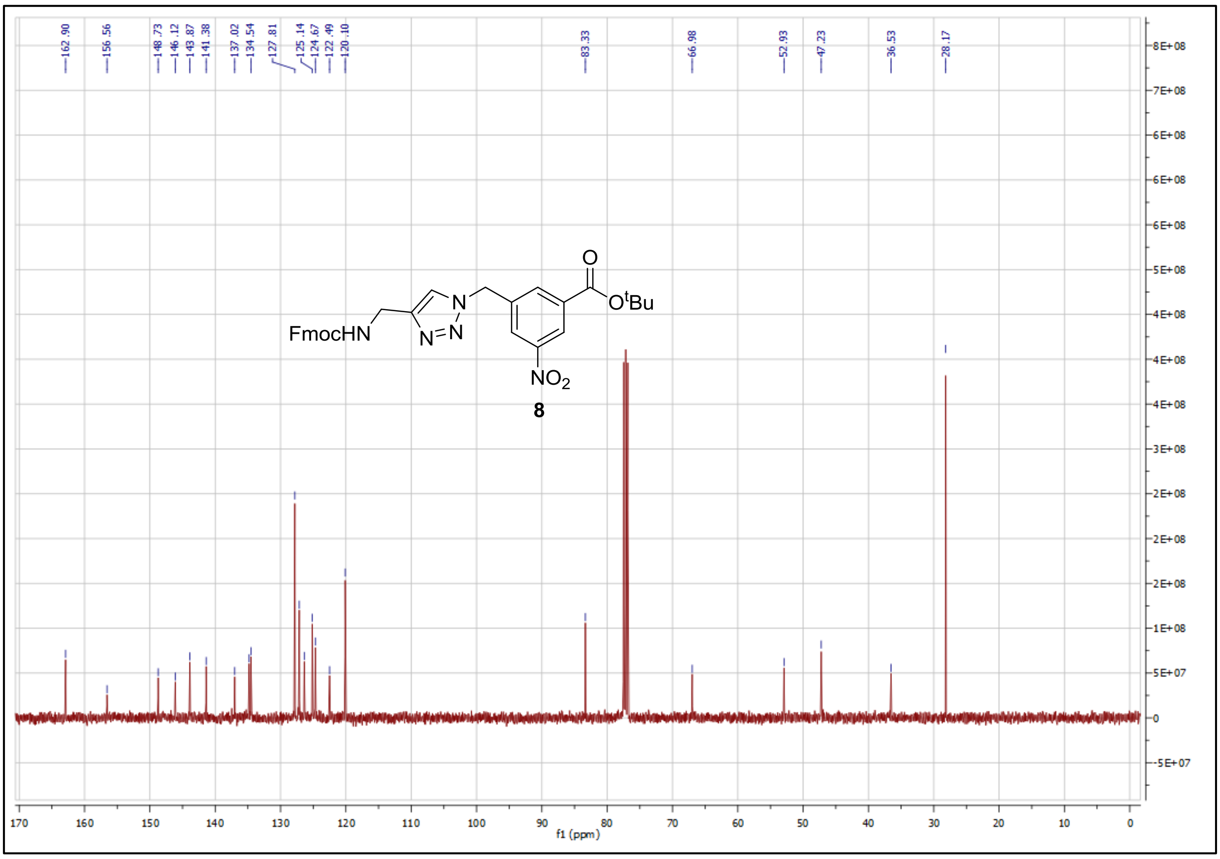

FmocHN
N
N=N
O
O^{t}Bu
NO$_2$
8
f1 (ppm)

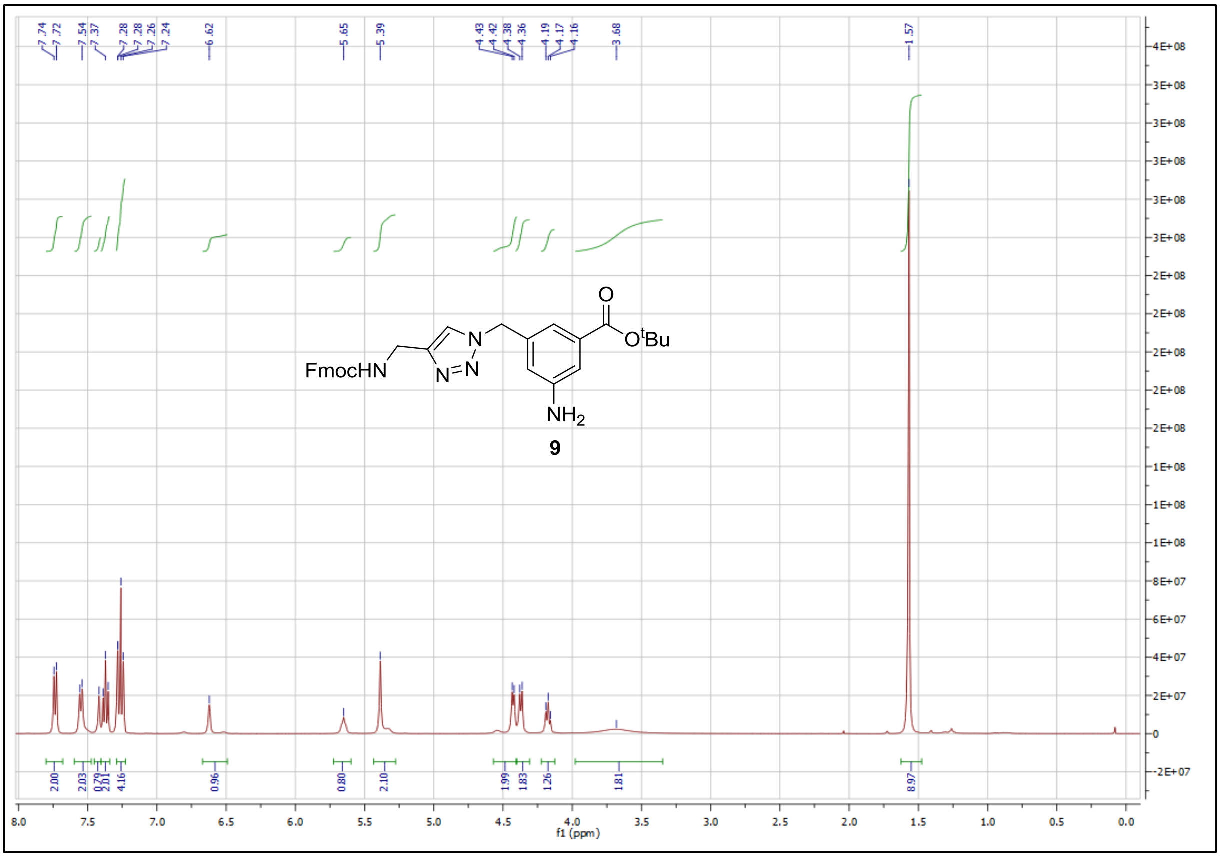

7.74
7.72
7.54
7.37
7.28
7.28
7.26
7.24
6.62
5.65
5.39
4.43
4.42
4.38
4.36
4.19
4.17
4.16
3.68
1.57
FmocHN
N
N=N
O
O^{t}Bu
NH$_2$
9
2.00
2.03
0.79
2.01
4.16
0.96
0.80
2.10
1.99
1.83
1.26
1.81
8.97
f1 (ppm)

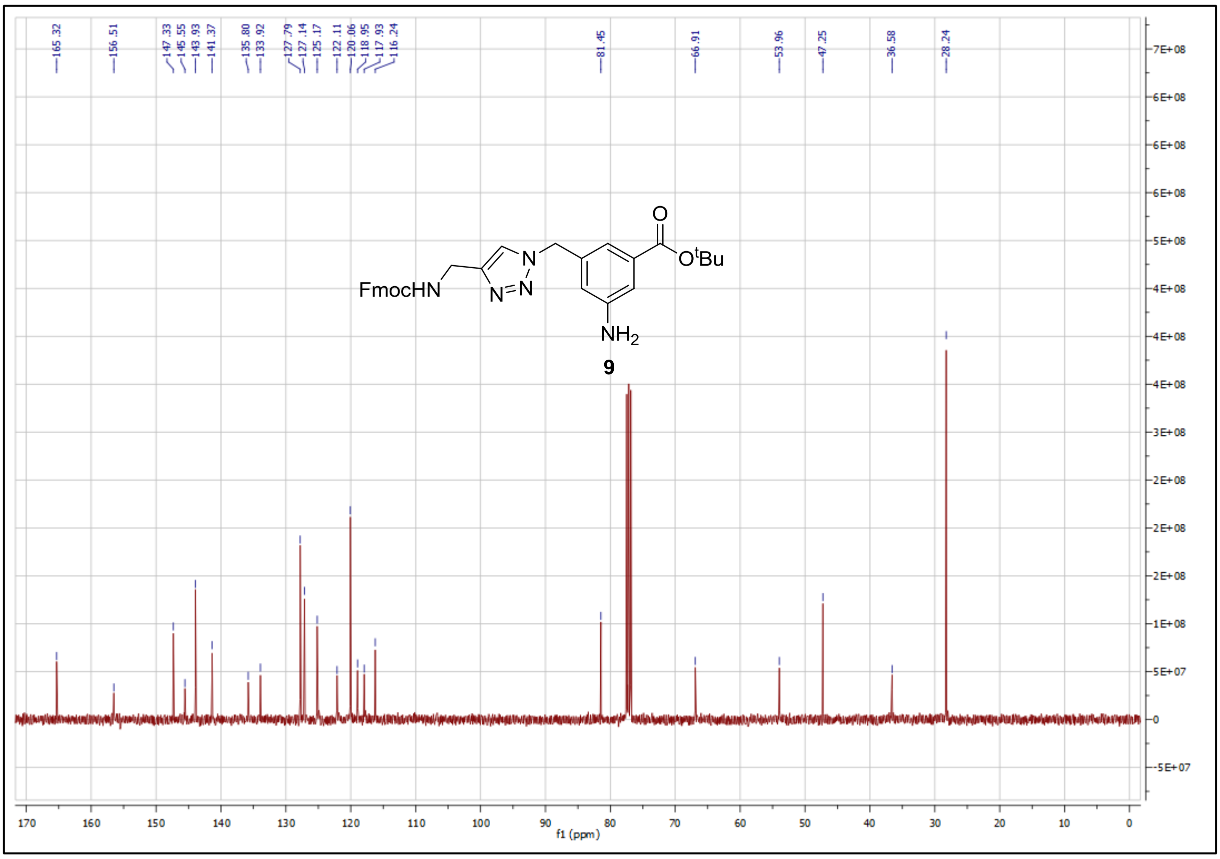

165.32
156.51
147.33
145.55
143.93
141.37
135.80
133.92
127.79
127.14
125.17
122.11
120.06
118.95
117.93
116.24
81.45
66.91
53.96
47.25
36.58
28.24
FmocHN
N
N=N
O
O^{t}Bu
NH$_2$
9
f1 (ppm)

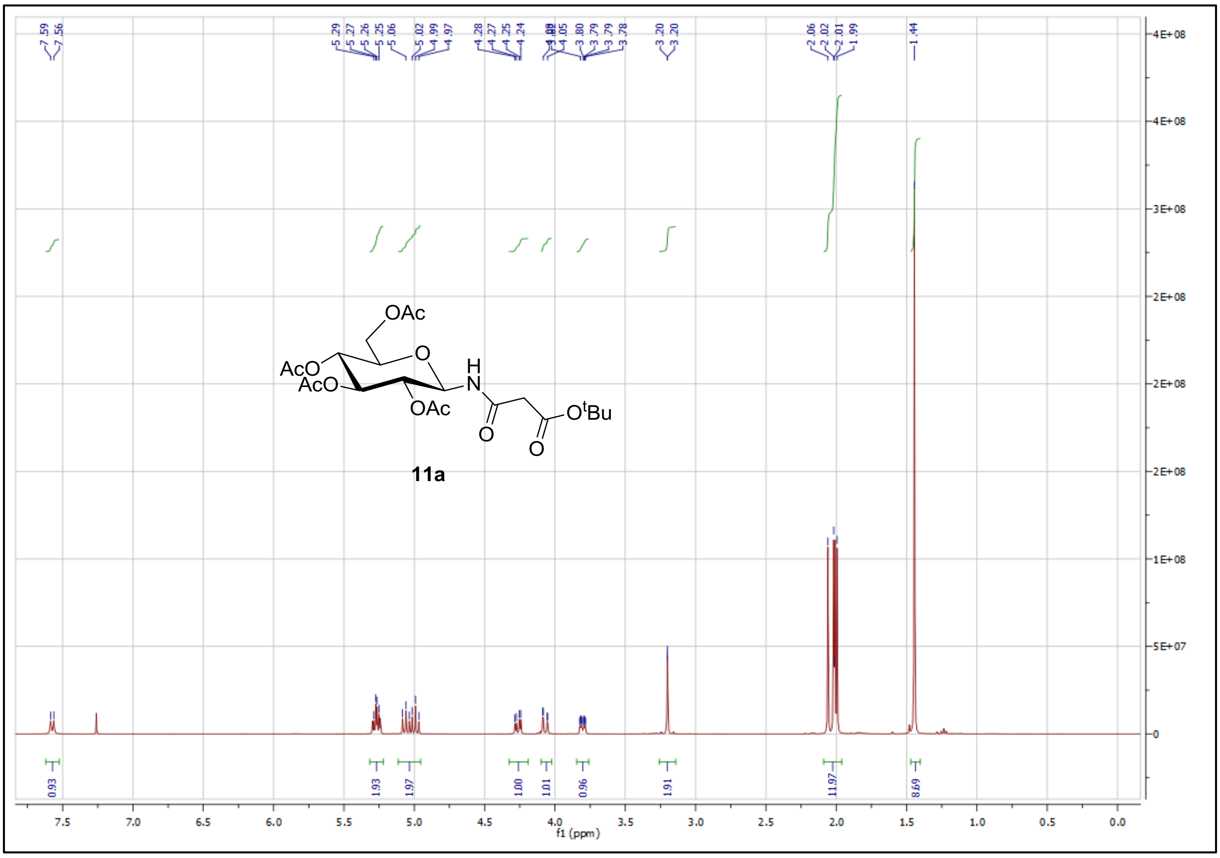
7.59
7.56
5.29
5.27
5.26
5.25
5.06
5.02
4.99
4.97
4.28
4.27
4.25
4.24
4.05
3.80
3.79
3.79
3.78
3.20
3.20
2.06
2.02
2.01
1.99
1.44
OAc
AcO
AcO
O
H
N
OAc
O
O
O^{t}Bu
11a
0.93
1.93
1.97
1.00
1.01
0.96
1.91
11.97
8.69
f1 (ppm)

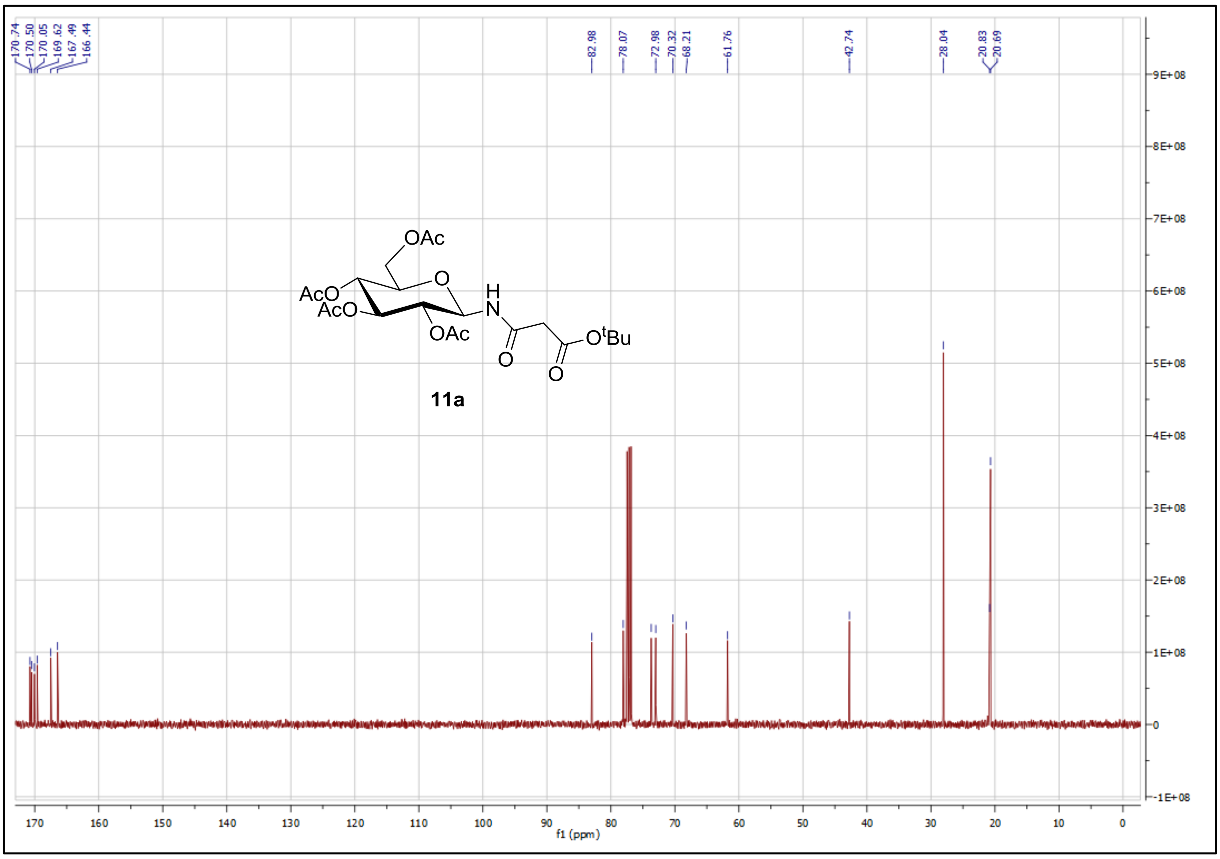
170.74
170.50
170.05
169.62
167.49
166.44
82.98
78.07
72.98
70.32
68.21
61.76
42.74
28.04
20.83
20.69
OAc
AcO
AcO
O
H
N
OAc
O
O
O^{t}Bu
11a
f1 (ppm)

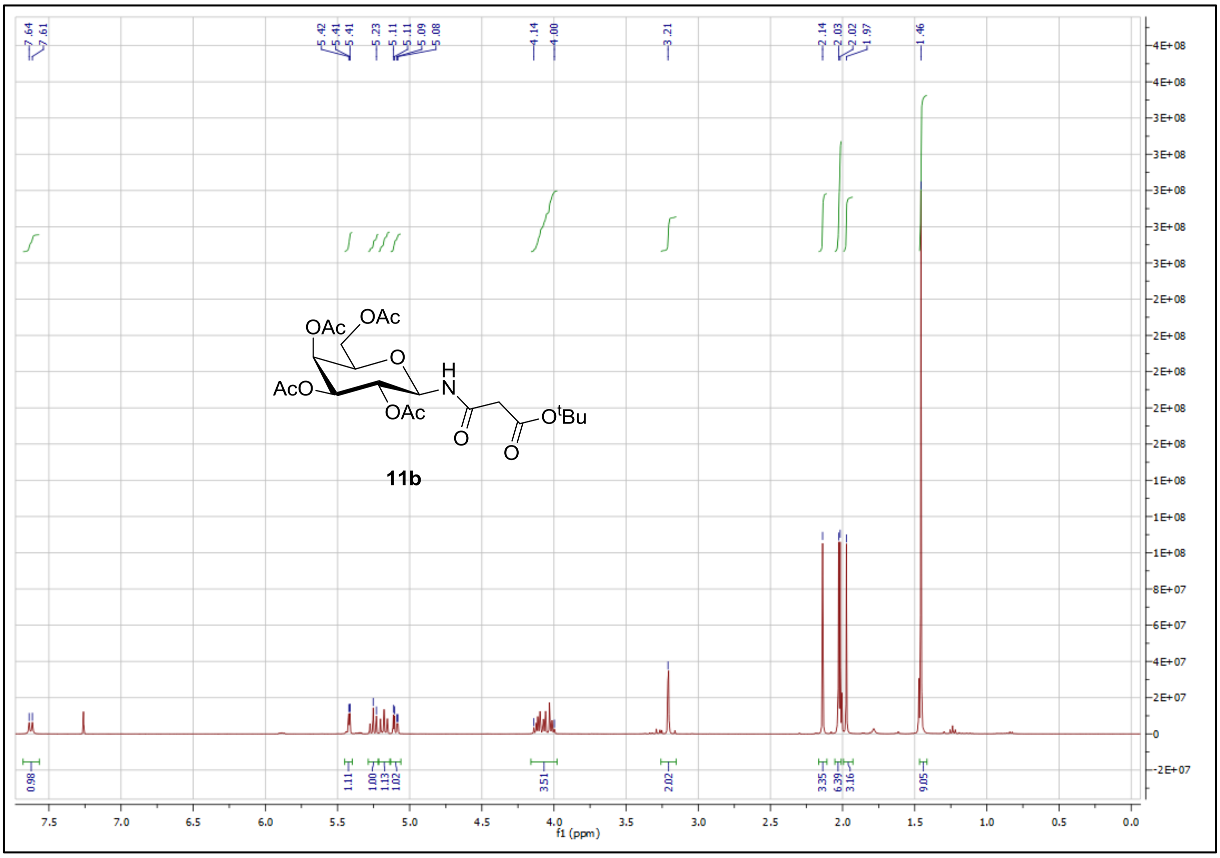
OAc
OAc
O
H
N
AcO
OAc
O^{t}Bu
O
O
11b
f1 (ppm)

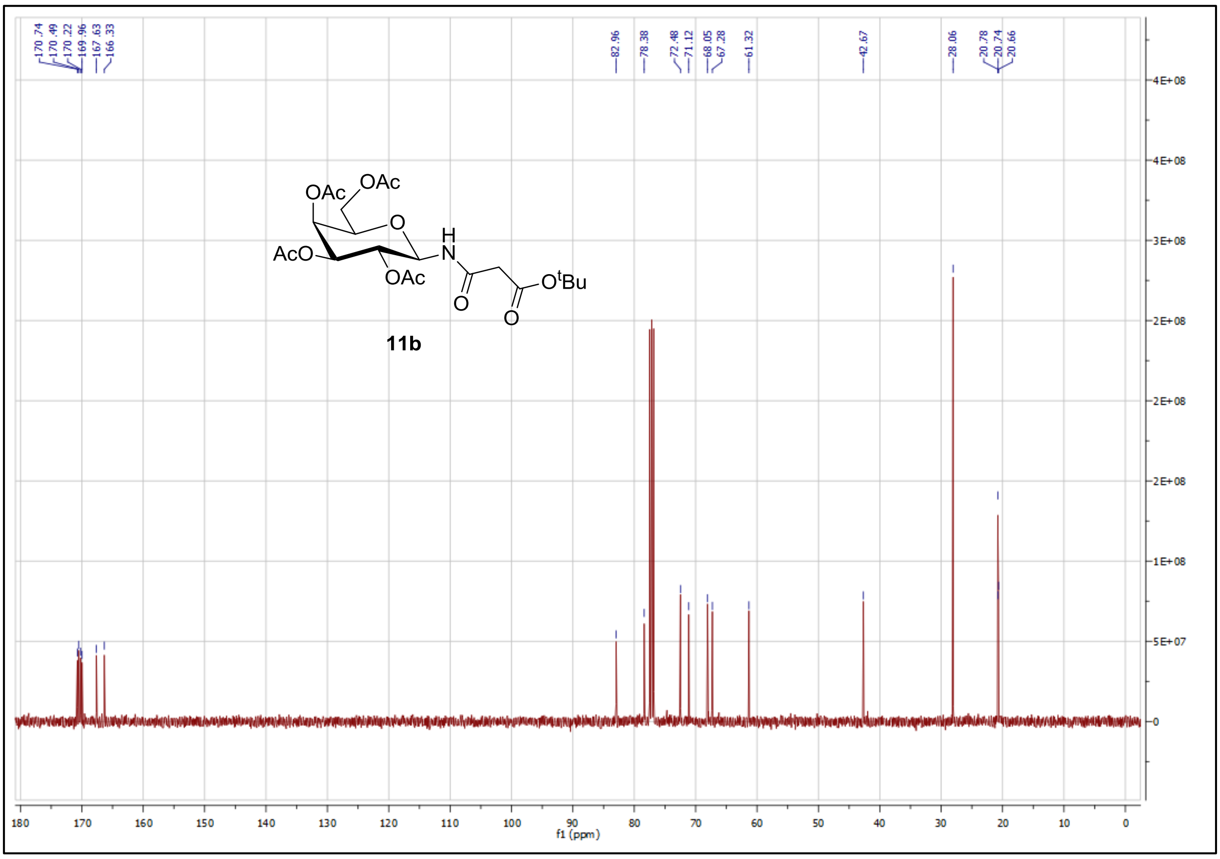
OAc
OAc
O
H
N
AcO
OAc
O^{t}Bu
O
O
11b
f1 (ppm)

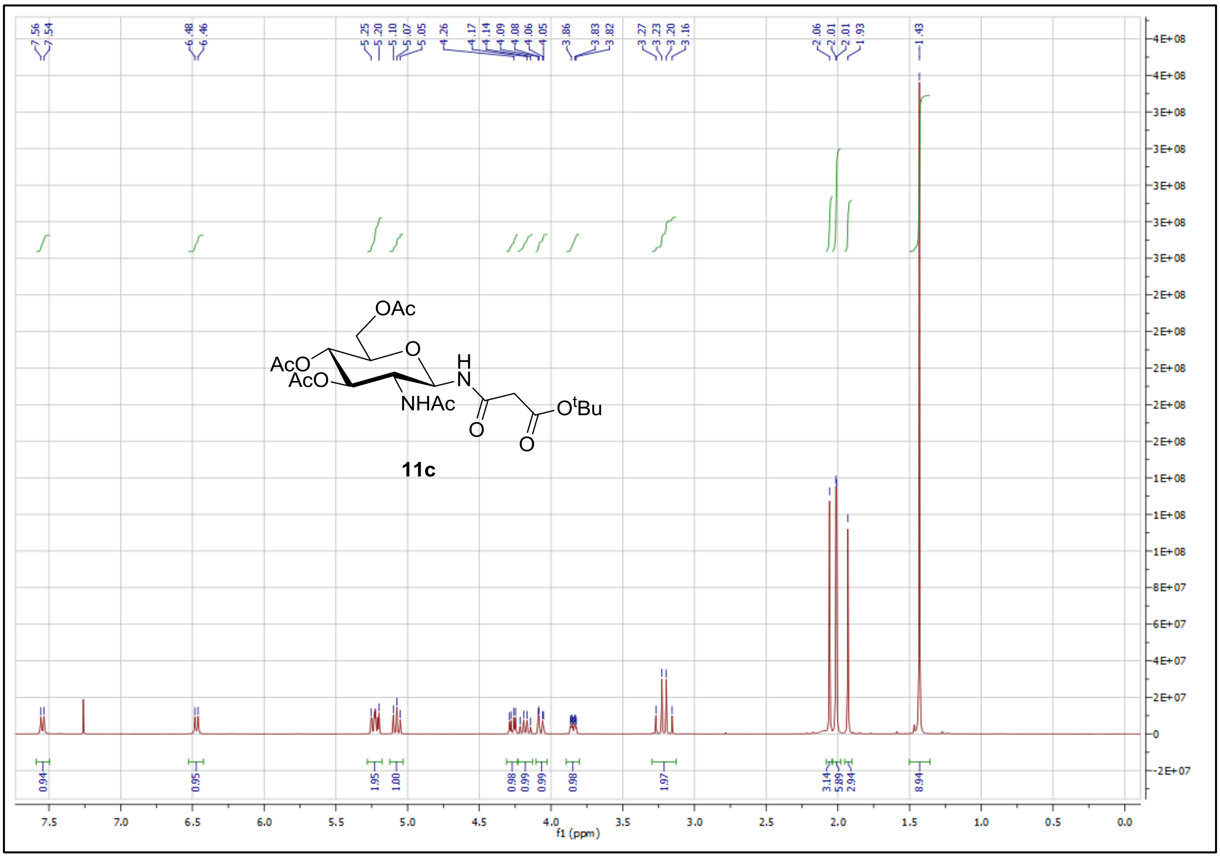

7.56 7.54 6.48 6.46 5.25 5.20 5.10 5.07 5.05 4.26 4.17 4.14 4.09 4.08 4.06 4.05 3.86 3.83 3.82 3.27 3.23 3.20 3.16 2.06 2.01 2.01 1.93 1.43
OAc
AcO
AcO
O
H
N
NHAc
O
O
O^{t}Bu
11c
0.94 0.95 1.95 1.00 0.98 0.99 0.99 0.98 1.97 3.14 5.89 2.94 8.94
f1 (ppm)

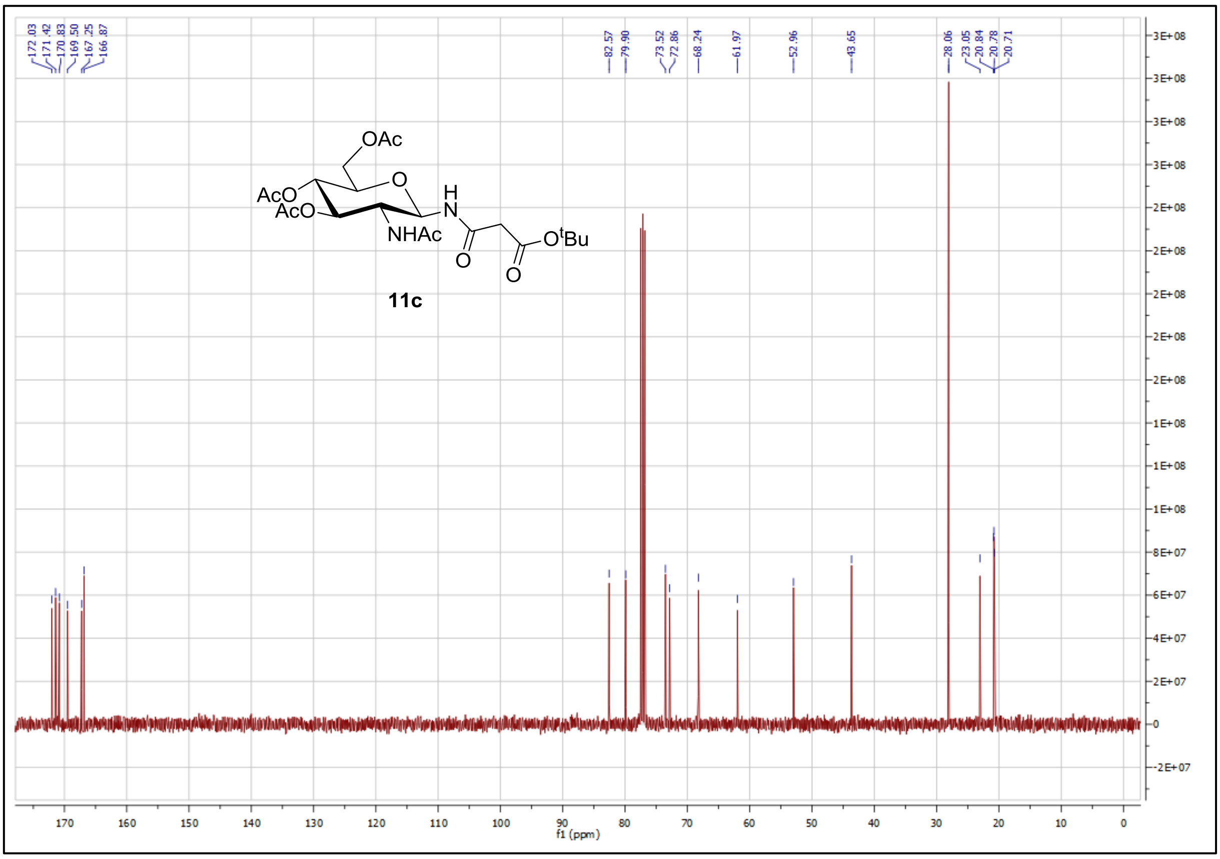

172.03 171.42 170.83 169.50 167.25 166.87 82.57 79.90 73.52 72.86 68.24 61.97 52.96 43.65 28.06 23.05 20.84 20.78 20.71
OAc
AcO
AcO
O
H
N
NHAc
O
O
O^{t}Bu
11c
f1 (ppm)

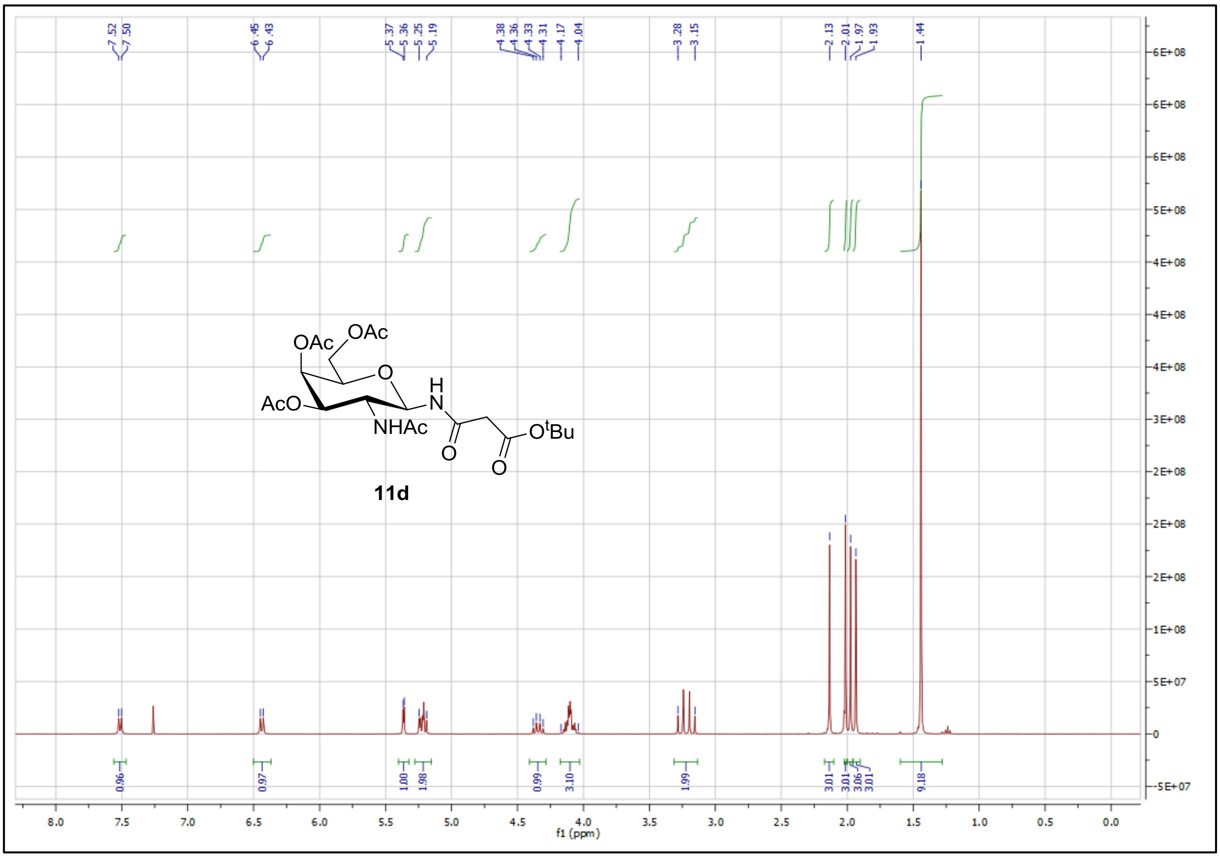

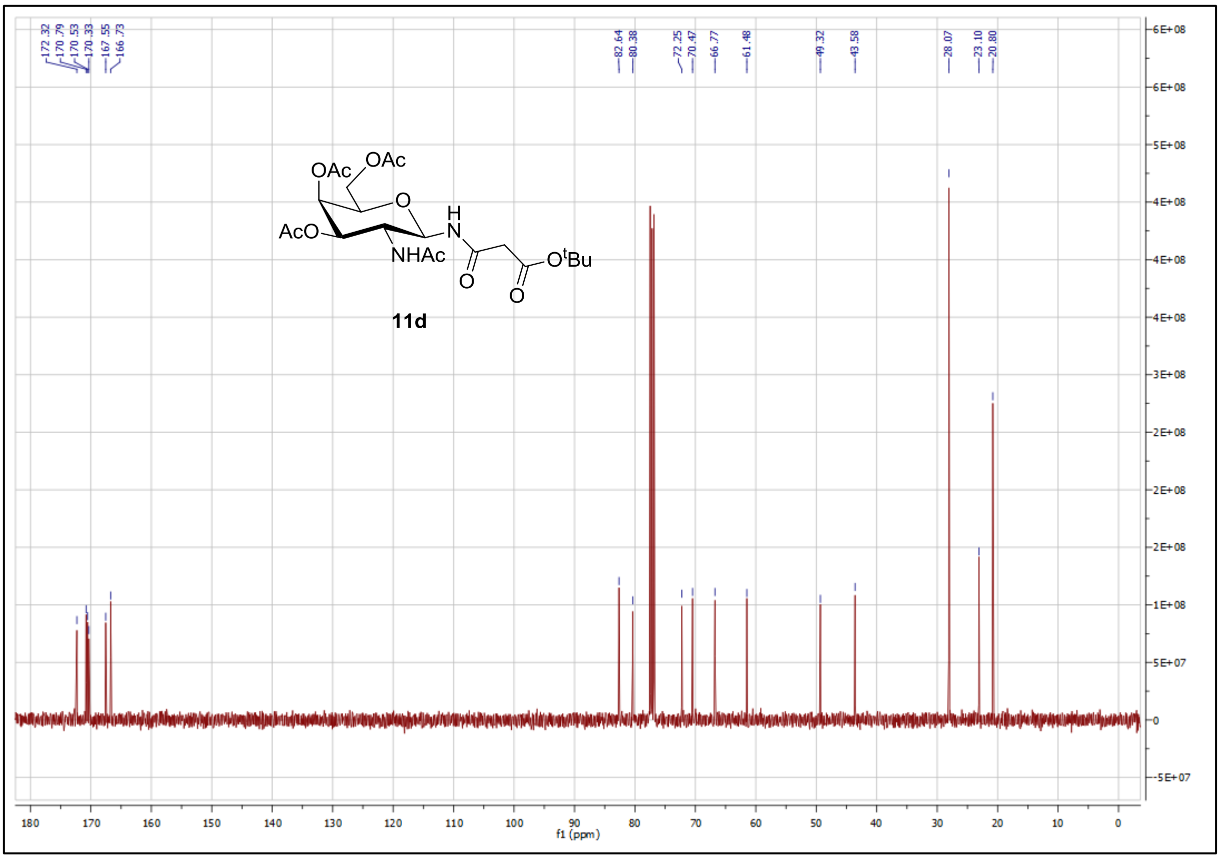

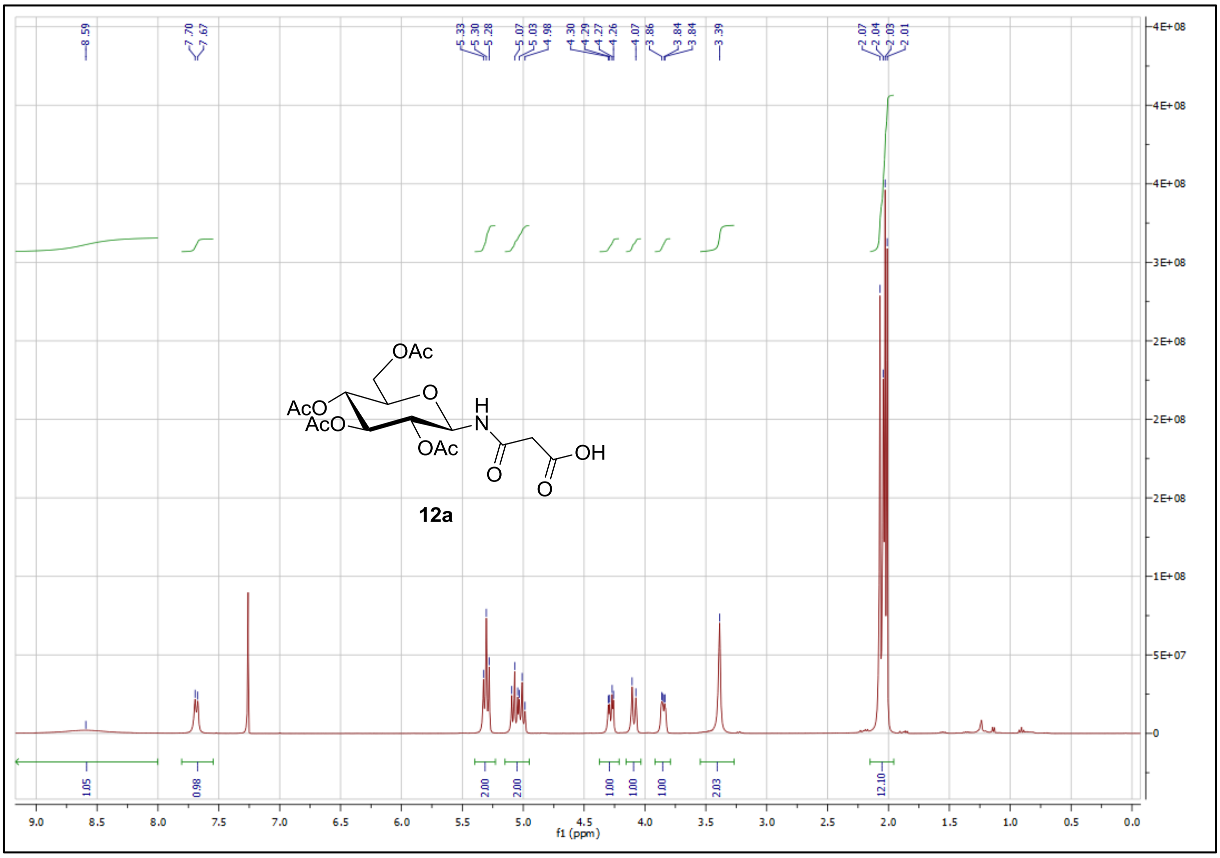
8.59
7.70
7.67
5.33
5.30
5.28
5.07
5.03
4.98
4.30
4.29
4.27
4.26
4.07
3.86
3.84
3.84
3.39
2.07
2.04
2.03
2.01
OAc
AcO
AcO
O
H
N
OAc
O
OH
O
12a
1.05
0.98
2.00
2.00
1.00
1.00
1.00
2.03
12.10
f1 (ppm)

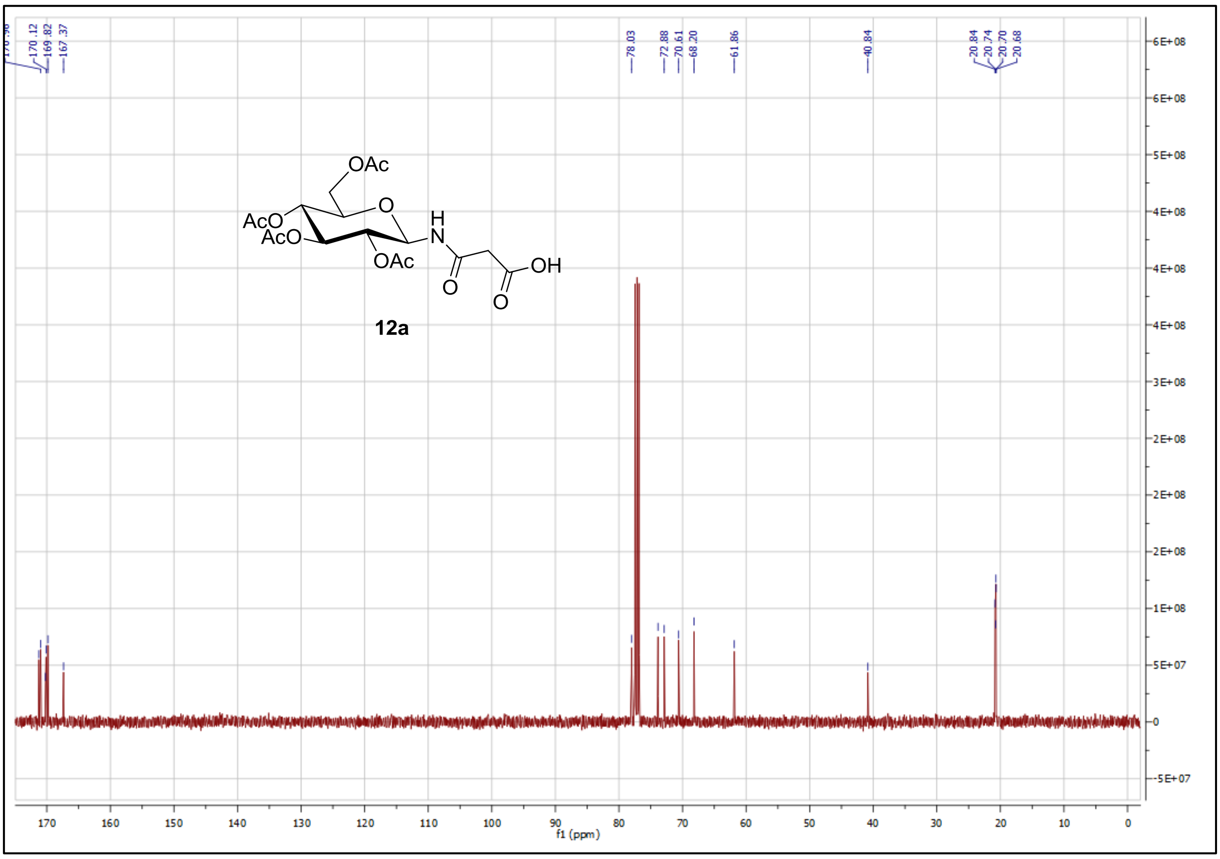
170.12
169.82
167.37
78.03
72.88
70.61
68.20
61.86
40.84
20.84
20.74
20.70
20.68
OAc
AcO
AcO
O
H
N
OAc
O
OH
O
12a
f1 (ppm)

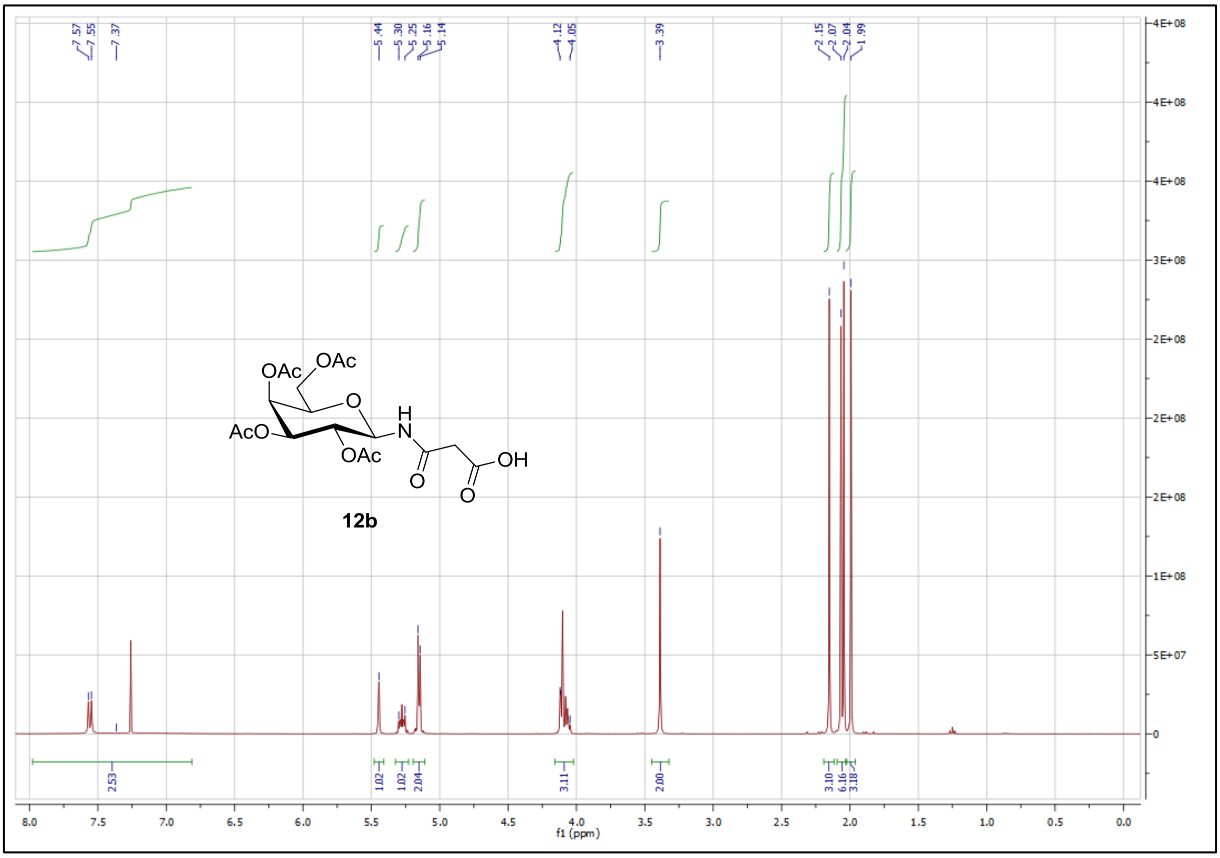
7.57
7.55
7.37
5.44
5.30
5.25
5.16
5.14
4.12
4.05
3.39
2.15
2.07
2.04
1.99
OAc
OAc
AcO
O
H
N
OAc
OH
O
O
12b
2.53
1.02
1.02
2.04
3.11
2.00
3.10
6.16
3.18
f1 (ppm)

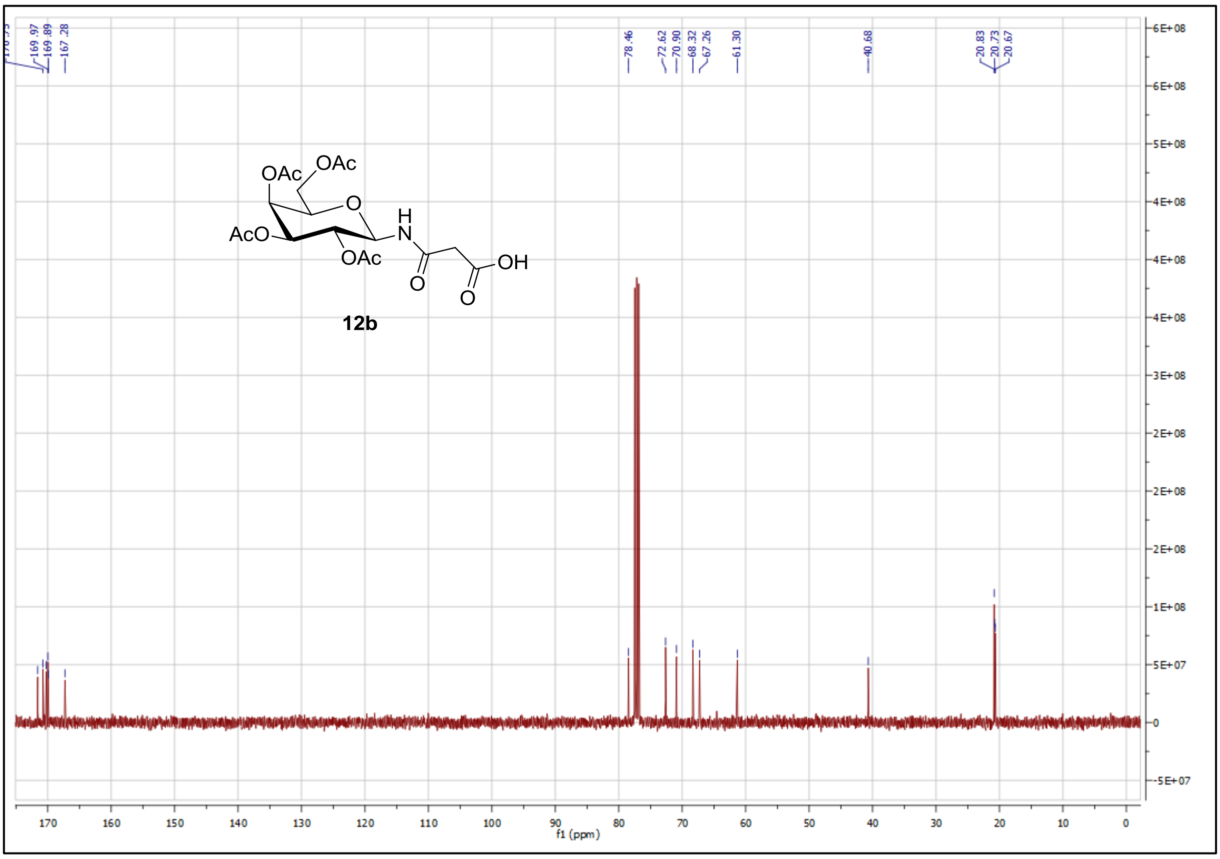
169.97
169.89
167.28
78.46
72.62
70.90
68.32
67.26
61.30
40.68
20.83
20.73
20.67
OAc
OAc
AcO
O
H
N
OAc
OH
O
O
12b
f1 (ppm)

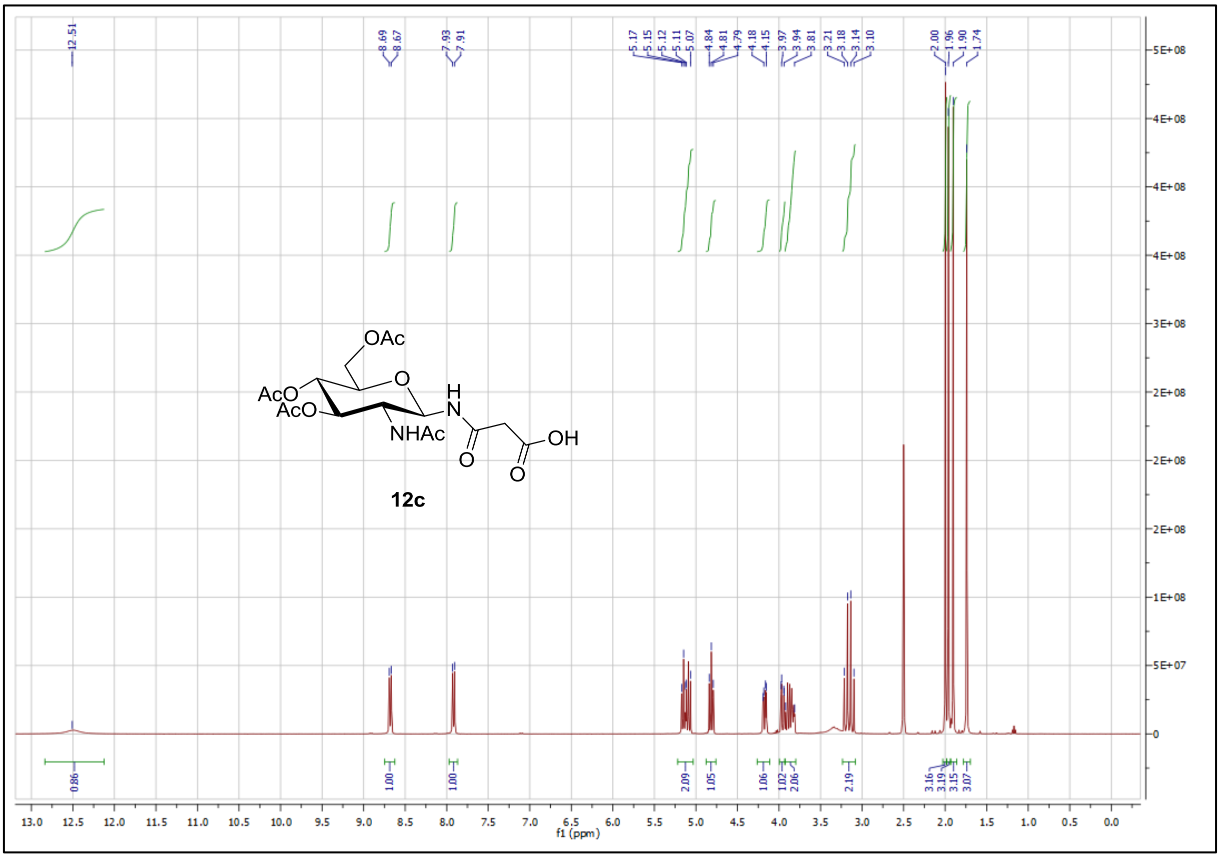
12.51
8.69
8.67
7.93
7.91
5.17
5.15
5.12
5.11
5.07
4.84
4.81
4.79
4.18
4.15
3.97
3.94
3.81
3.21
3.18
3.14
3.10
2.00
1.96
1.90
1.74
OAc
AcO
AcO
O
H
N
NHAc
O
OH
O
12c
0.86
1.00
1.00
2.09
1.05
1.06
1.02
2.06
2.19
3.16
3.19
3.15
3.07
f1 (ppm)

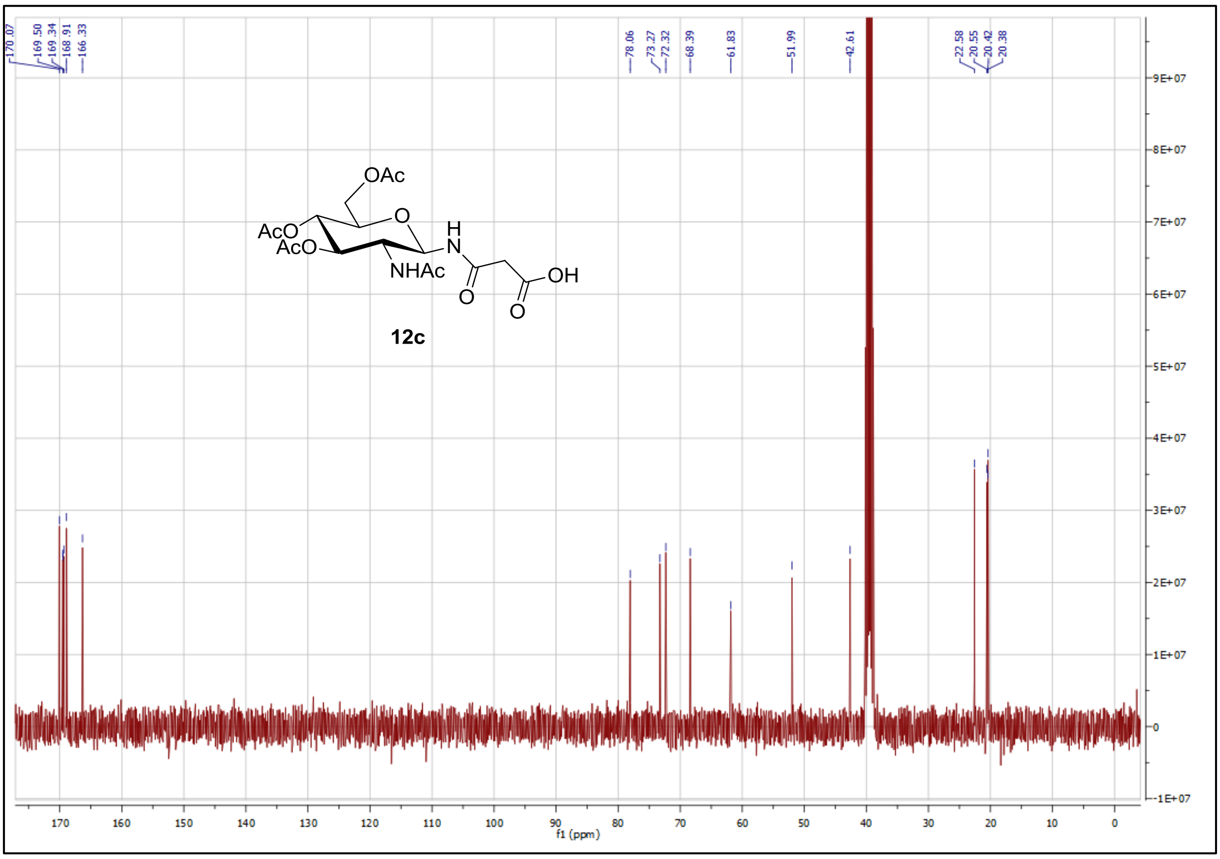
170.07
169.50
169.34
168.91
166.33
78.06
73.27
72.32
68.39
61.83
51.99
42.61
22.58
20.55
20.42
20.38
OAc
AcO
AcO
O
H
N
NHAc
O
OH
O
12c
f1 (ppm)

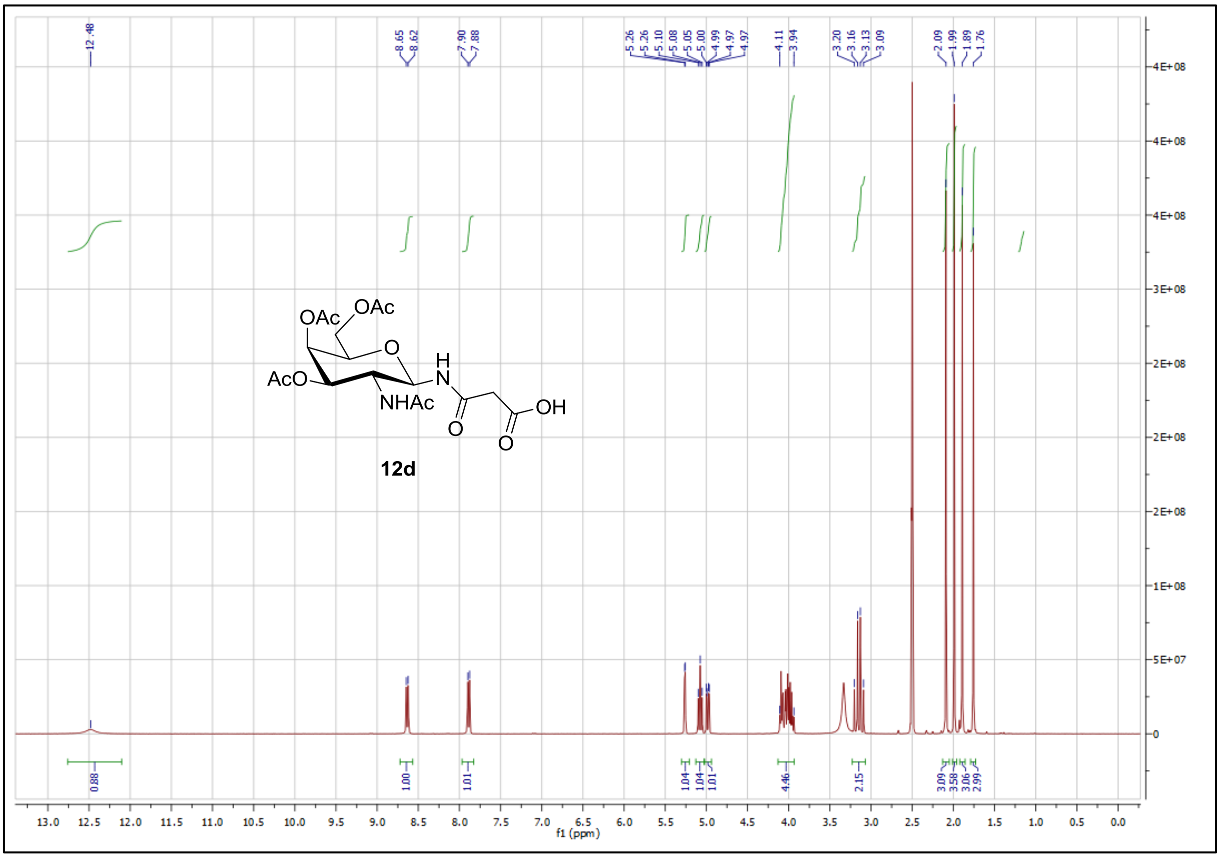
12.48
8.65
8.62
7.90
7.88
5.26
5.26
5.10
5.08
5.05
5.00
4.99
4.97
4.97
4.11
3.94
3.20
3.16
3.13
3.09
2.09
1.99
1.89
1.76
0.88
1.00
1.01
1.04
1.04
1.01
4.46
2.15
3.09
3.58
3.06
2.99
OAc
OAc
AcO
O
H
N
NHAc
O
OH
O
12d
f1 (ppm)

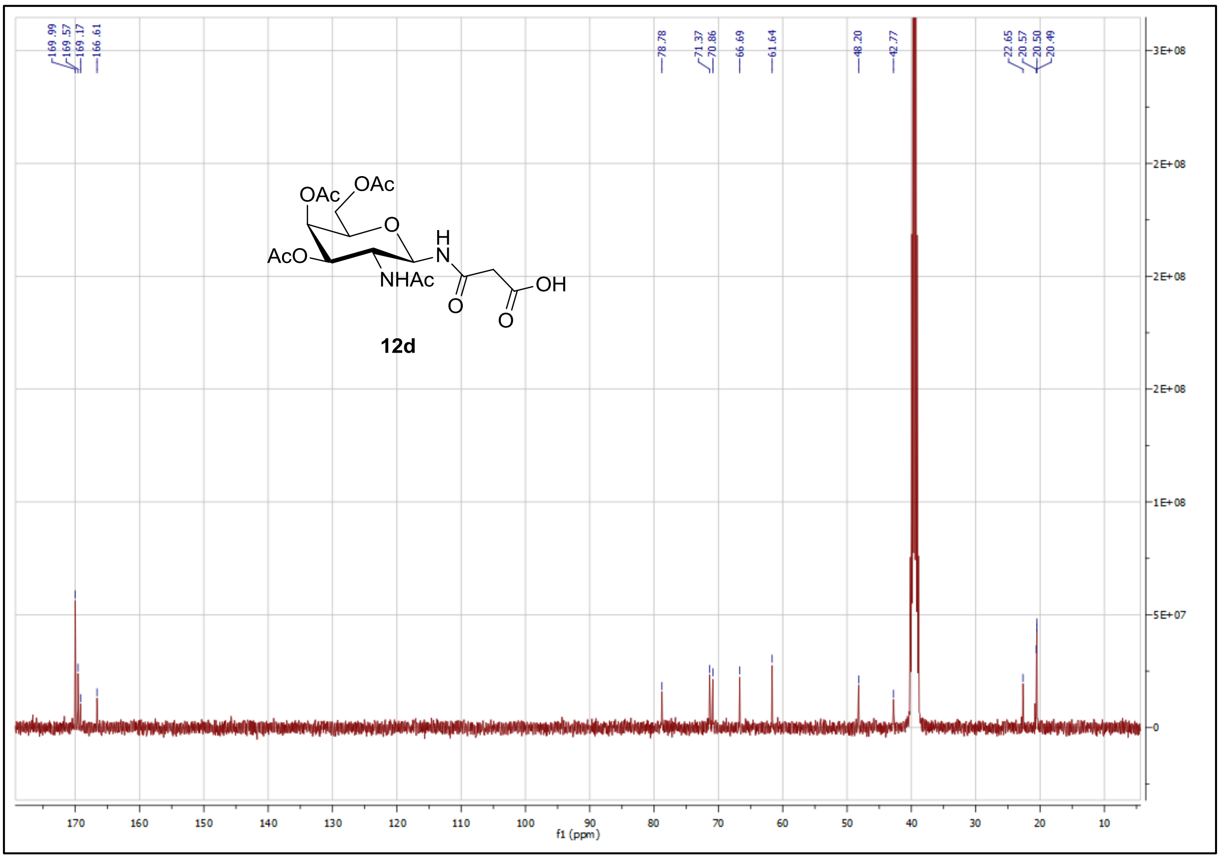
169.99
169.57
169.17
166.61
78.78
71.37
70.86
66.69
61.64
48.20
42.77
22.65
20.57
20.50
20.49
OAc
OAc
AcO
O
H
N
NHAc
O
OH
O
12d
f1 (ppm)

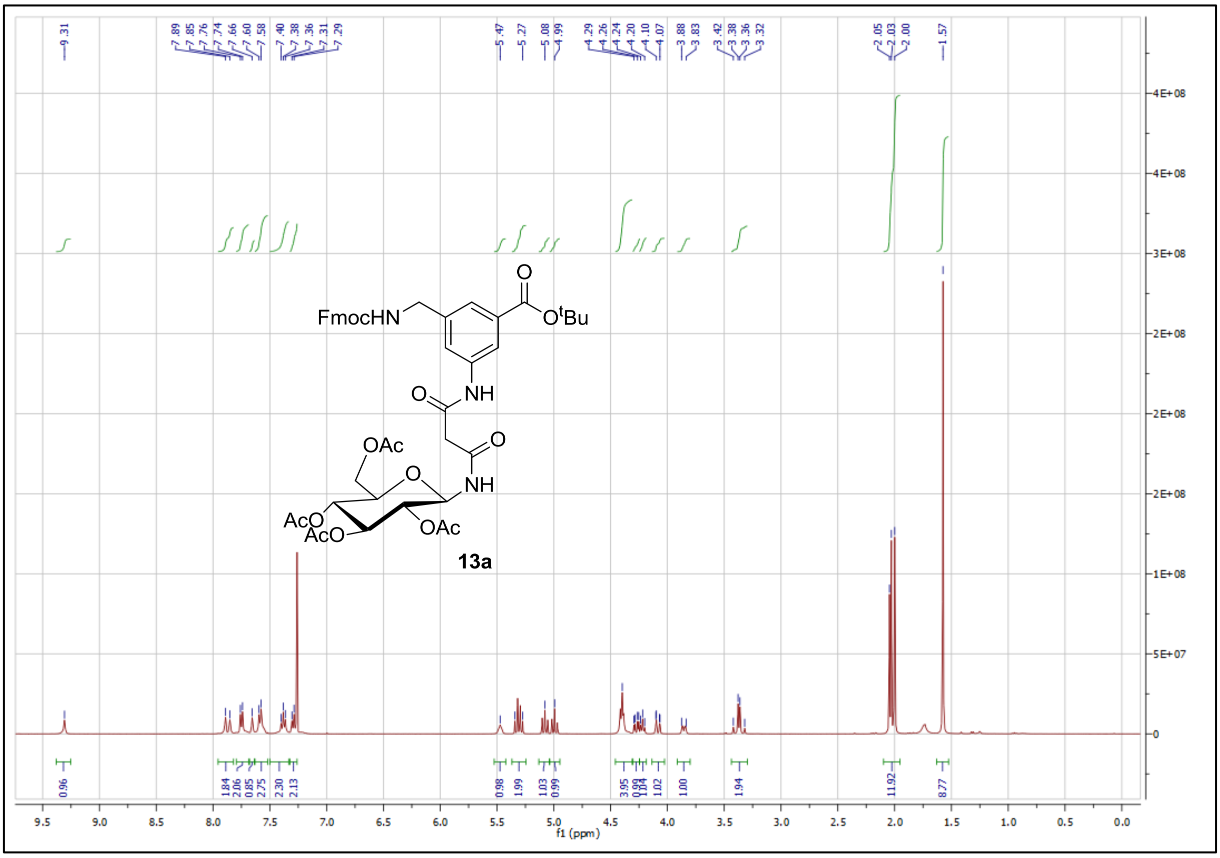

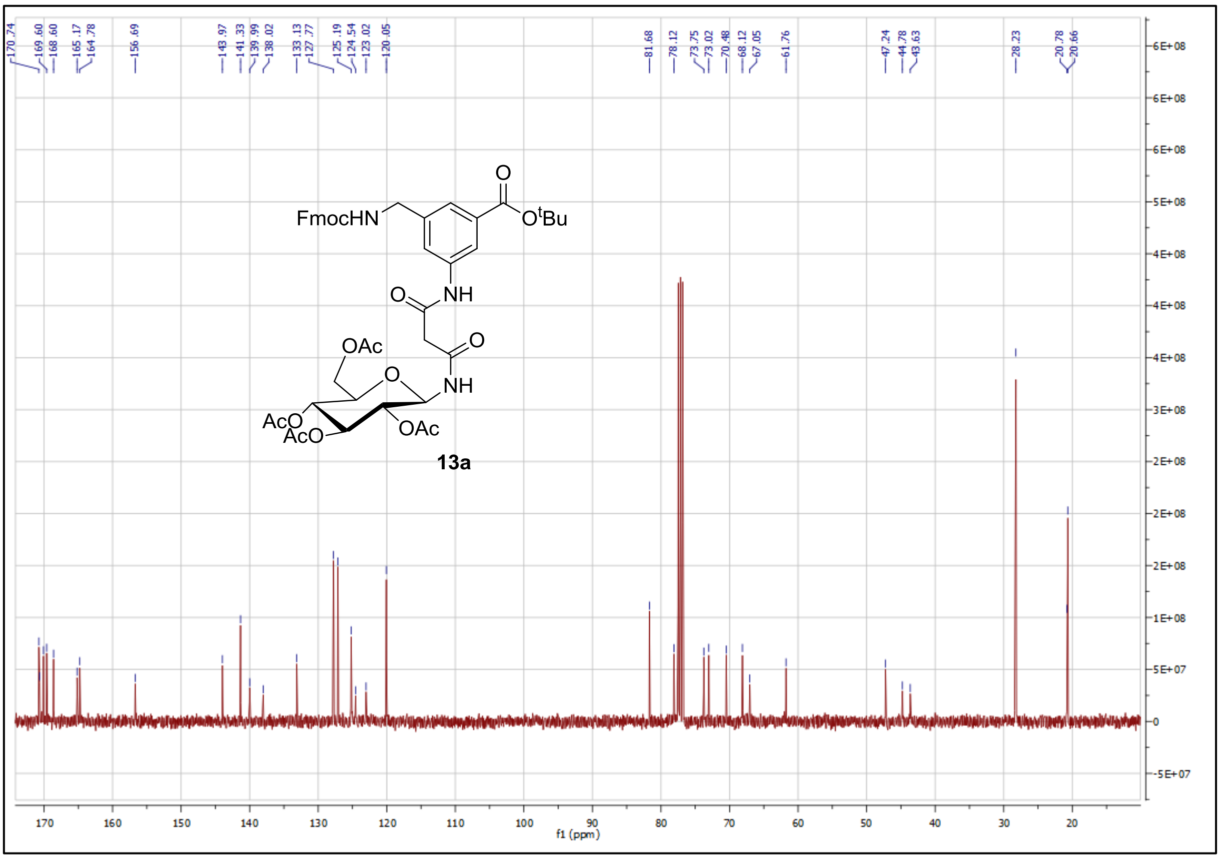

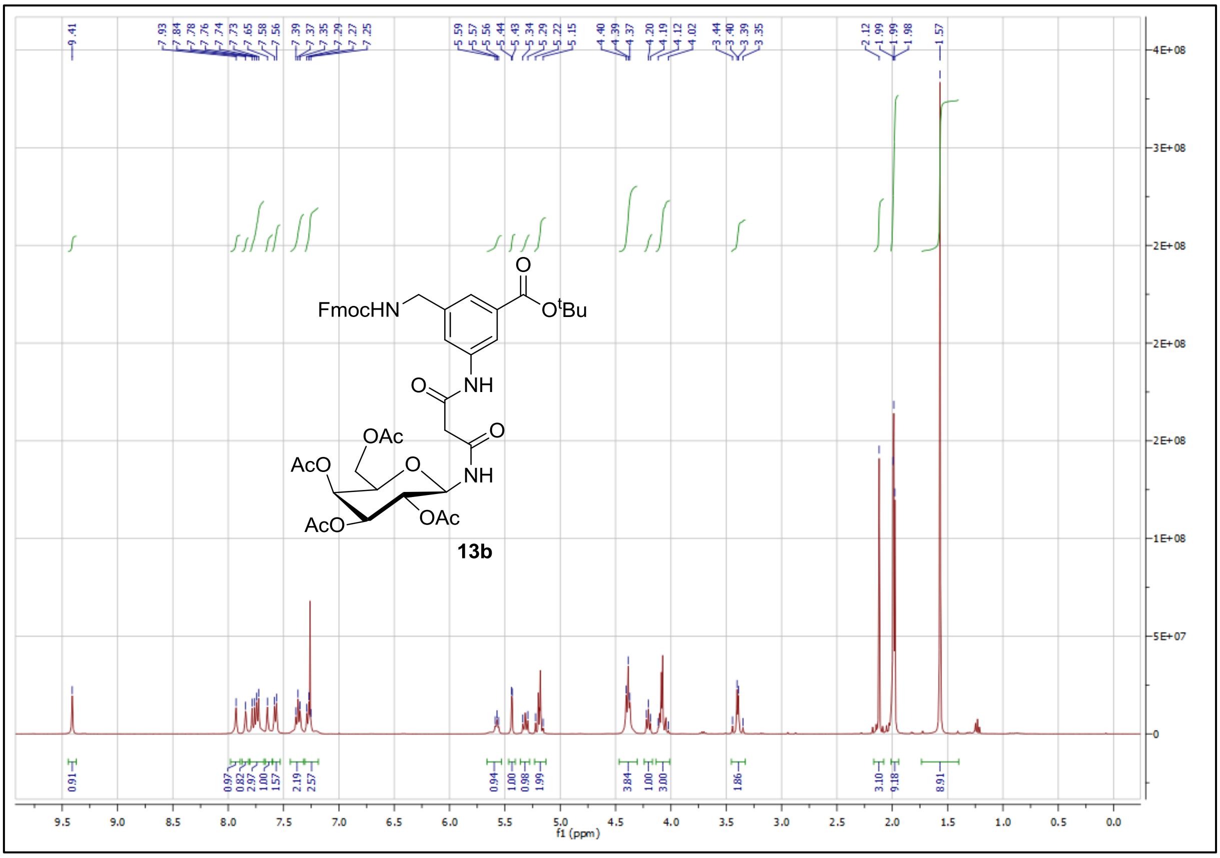

FmocHN
O
O^{t}Bu
NH
O
O
OAc
AcO
O
NH
AcO
OAc
13b
f1 (ppm)

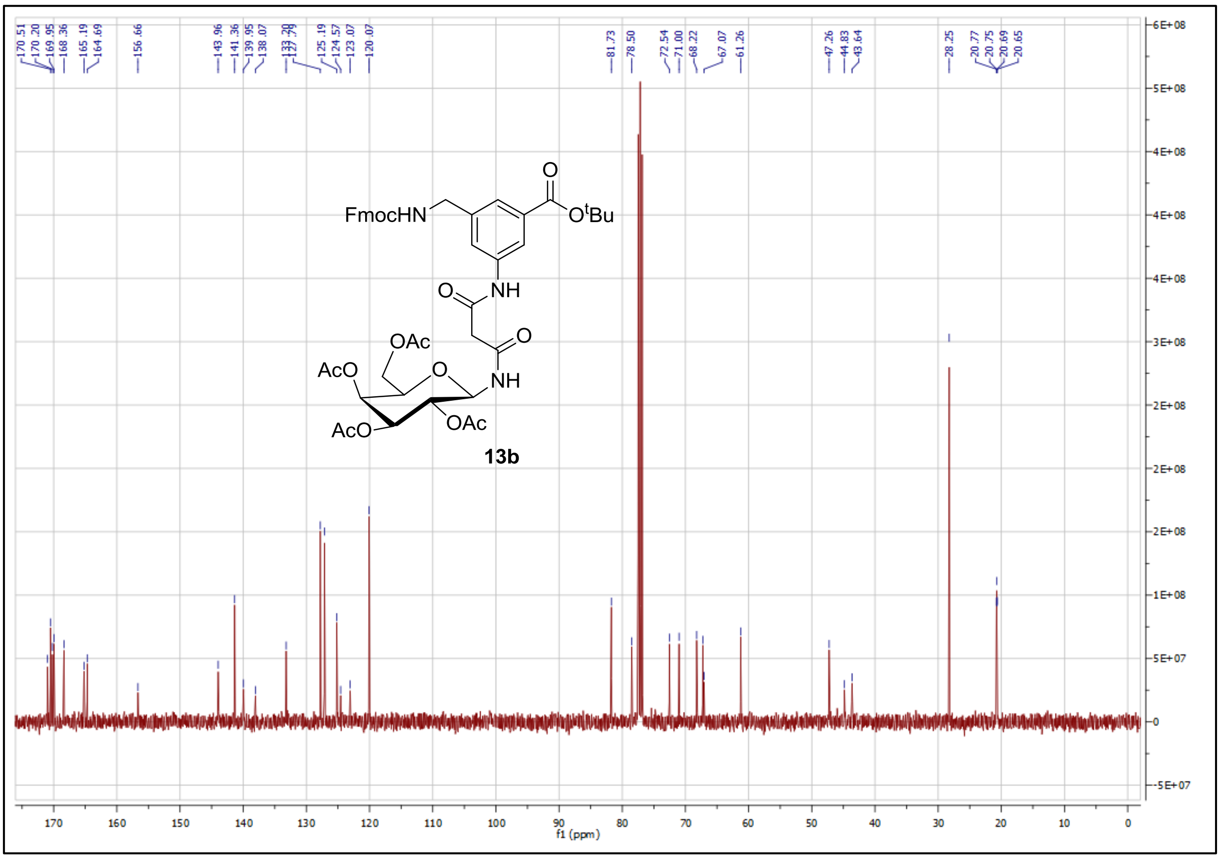

FmocHN
O
O^{t}Bu
NH
O
O
OAc
AcO
O
NH
AcO
OAc
13b
f1 (ppm)

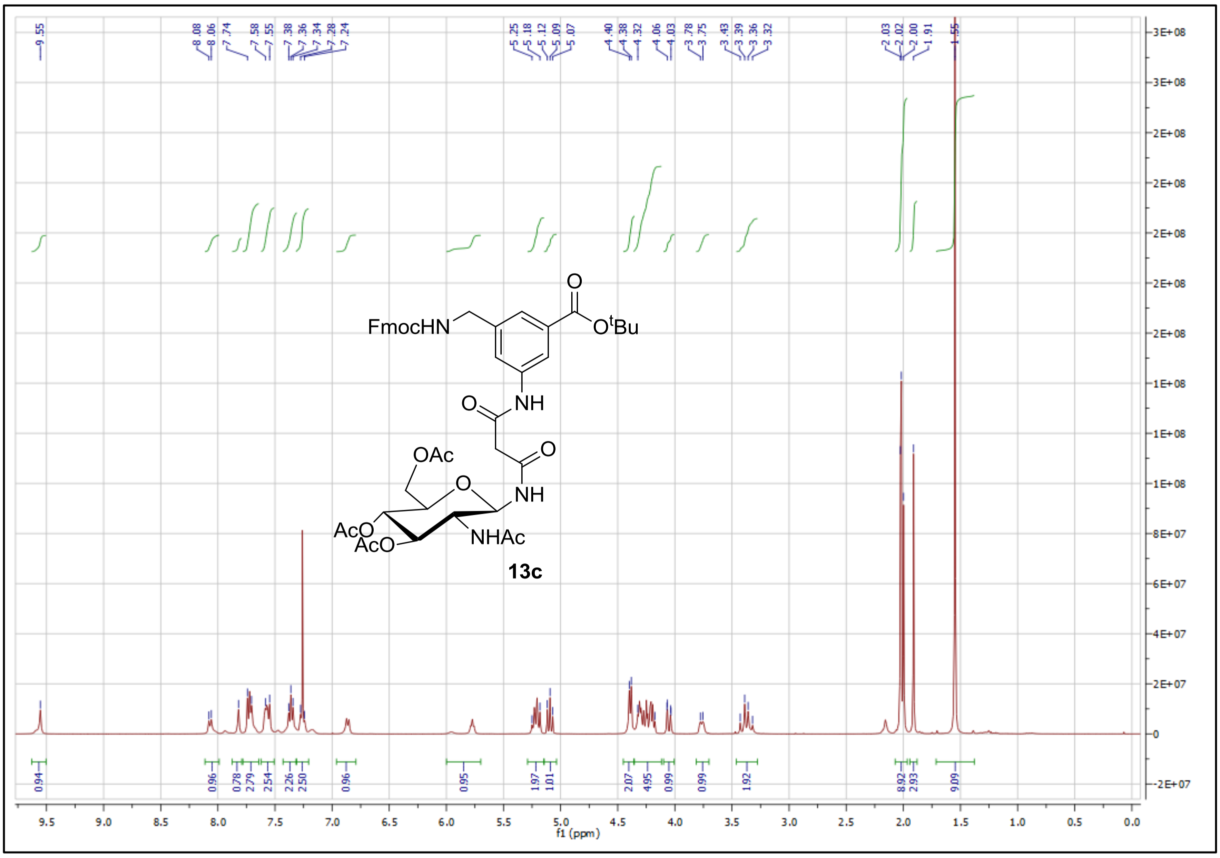

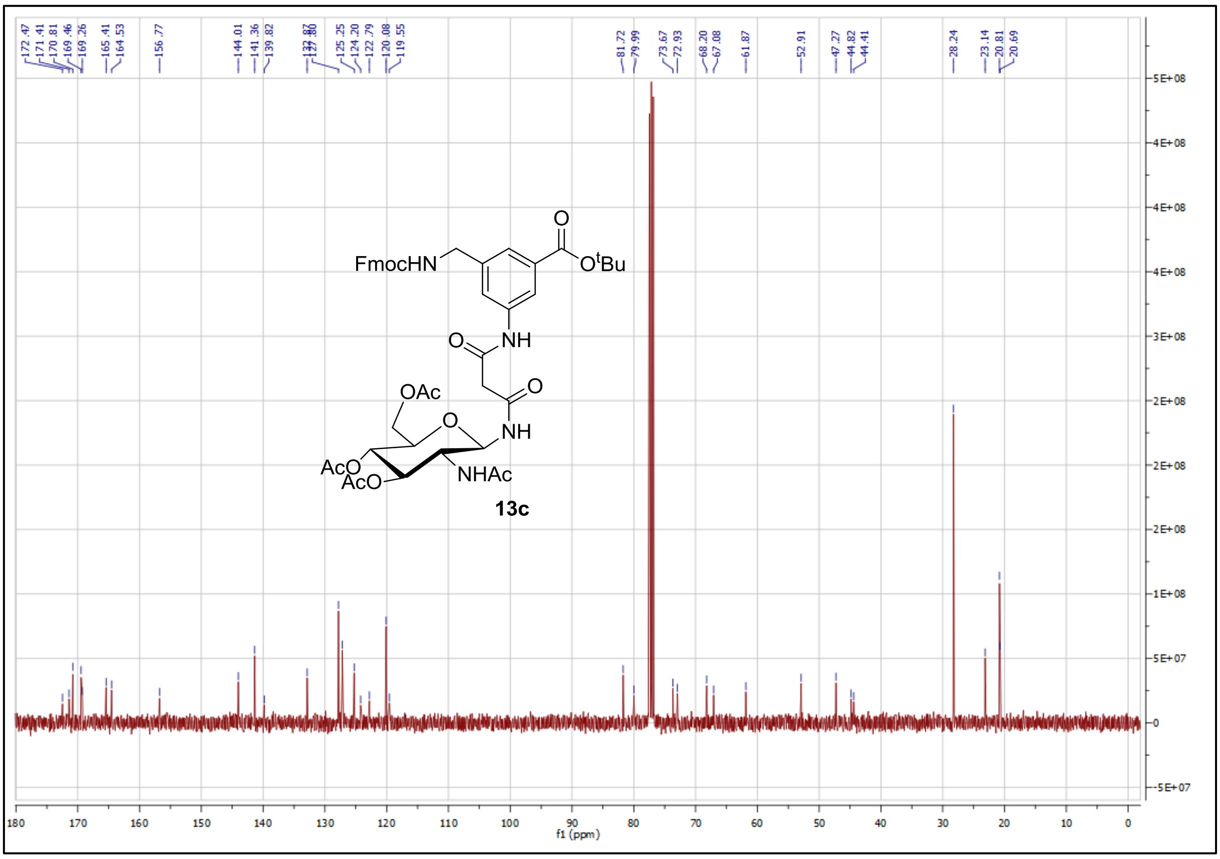

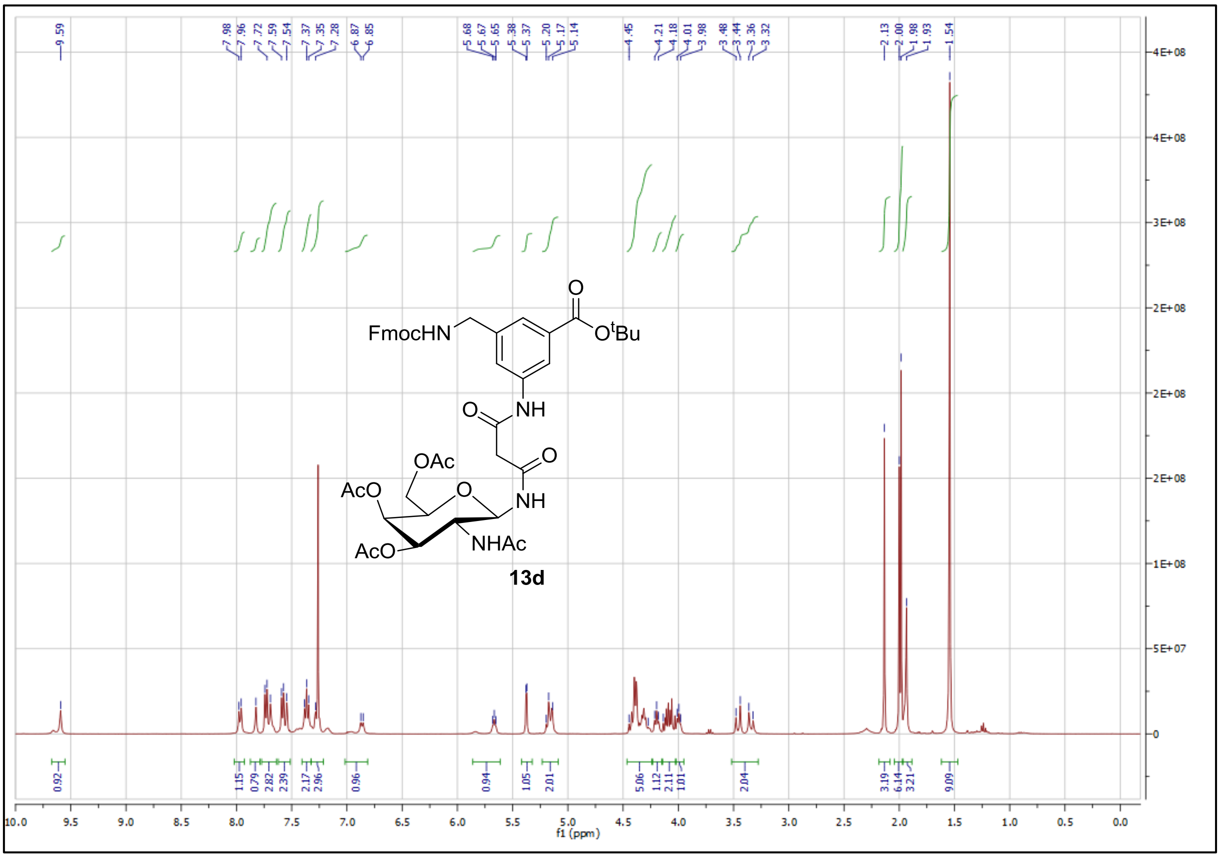
FmocHN
O
O^tBu
O
NH
OAc
O
AcO
O
NH
AcO
NHAc
13d
f1 (ppm)

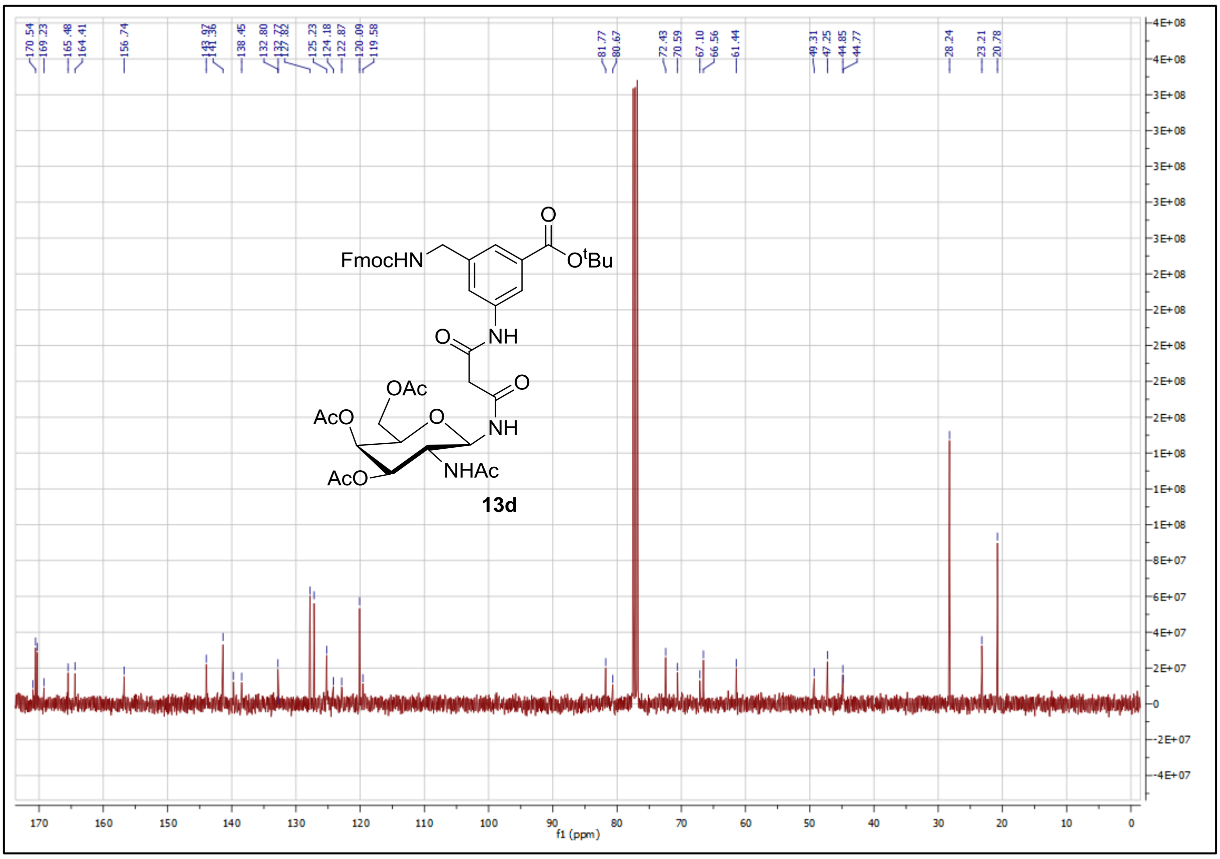
FmocHN
O
O^tBu
O
NH
OAc
O
AcO
O
NH
AcO
NHAc
13d
f1 (ppm)

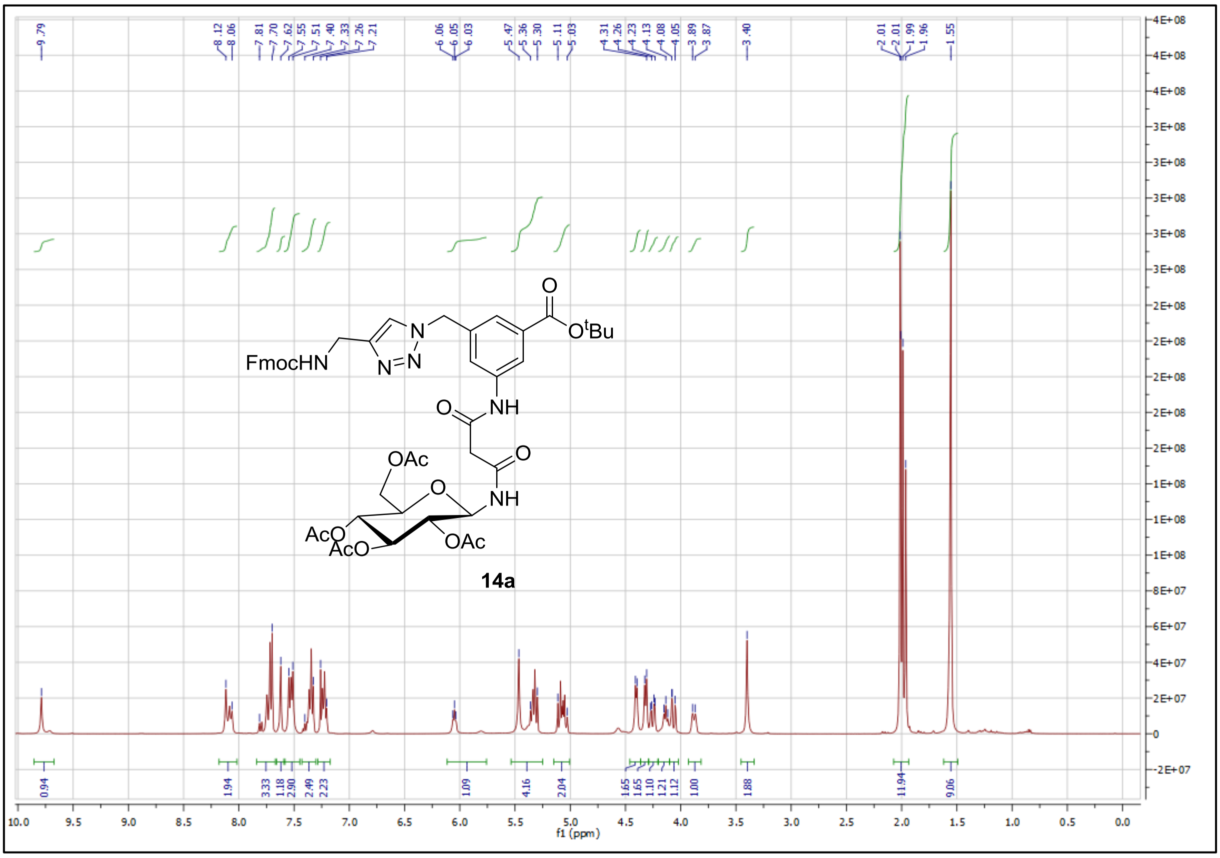

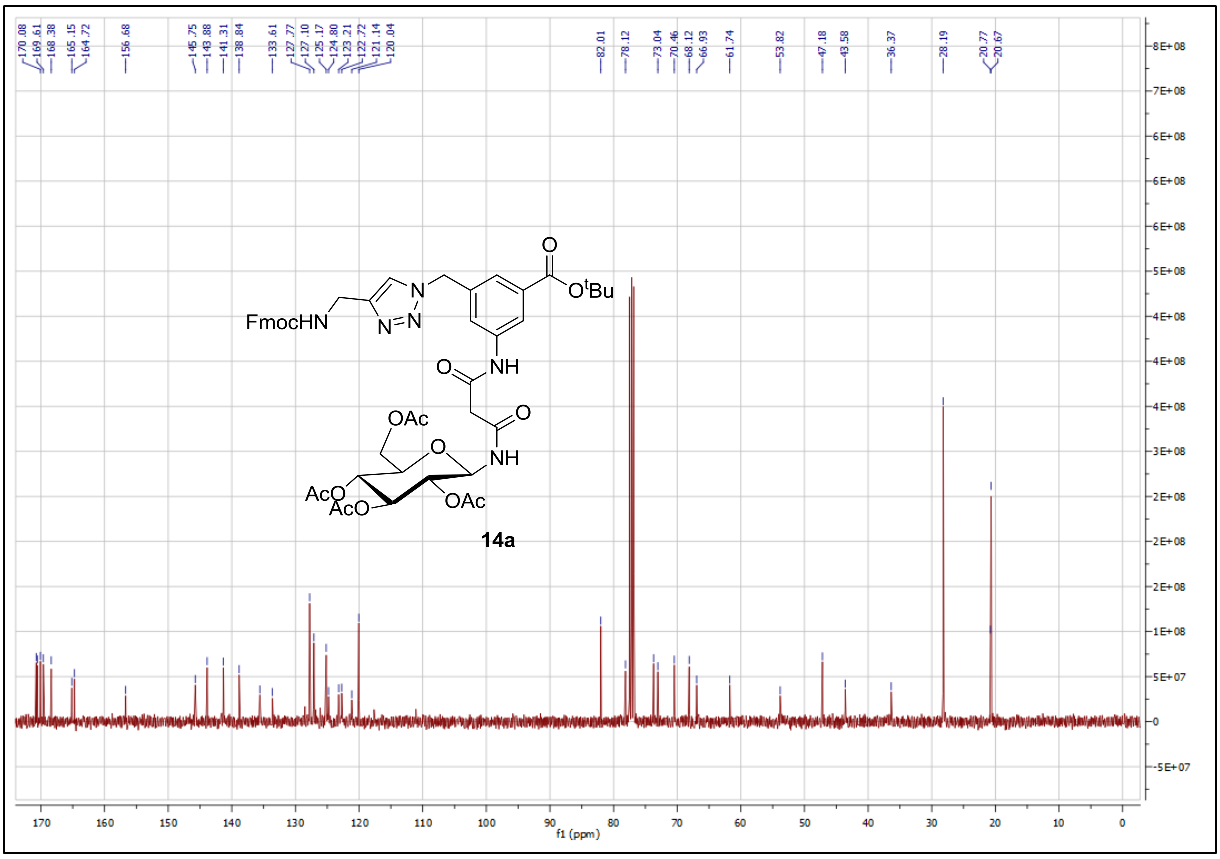

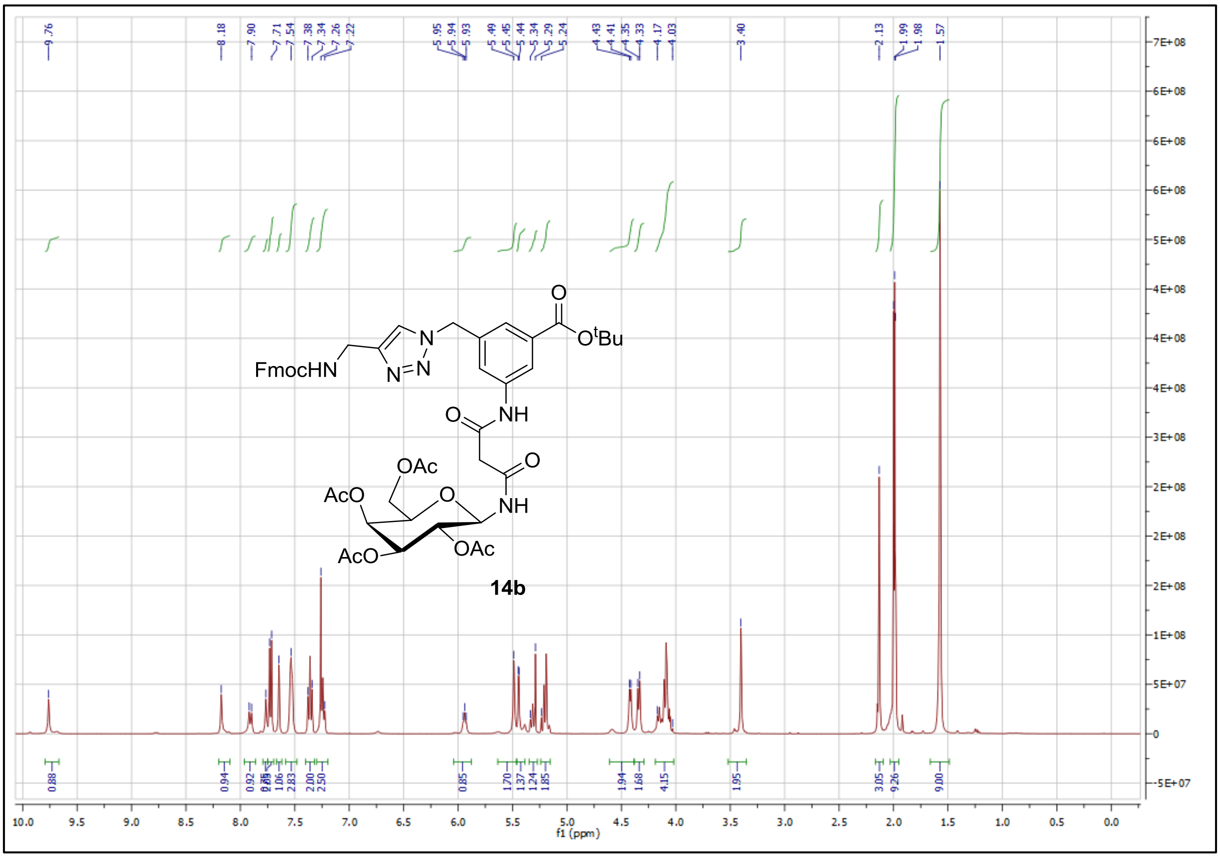
FmocHN
O
OᵗBu
NH
O
O
NH
OAc
AcO
AcO
OAc
14b
f1 (ppm)

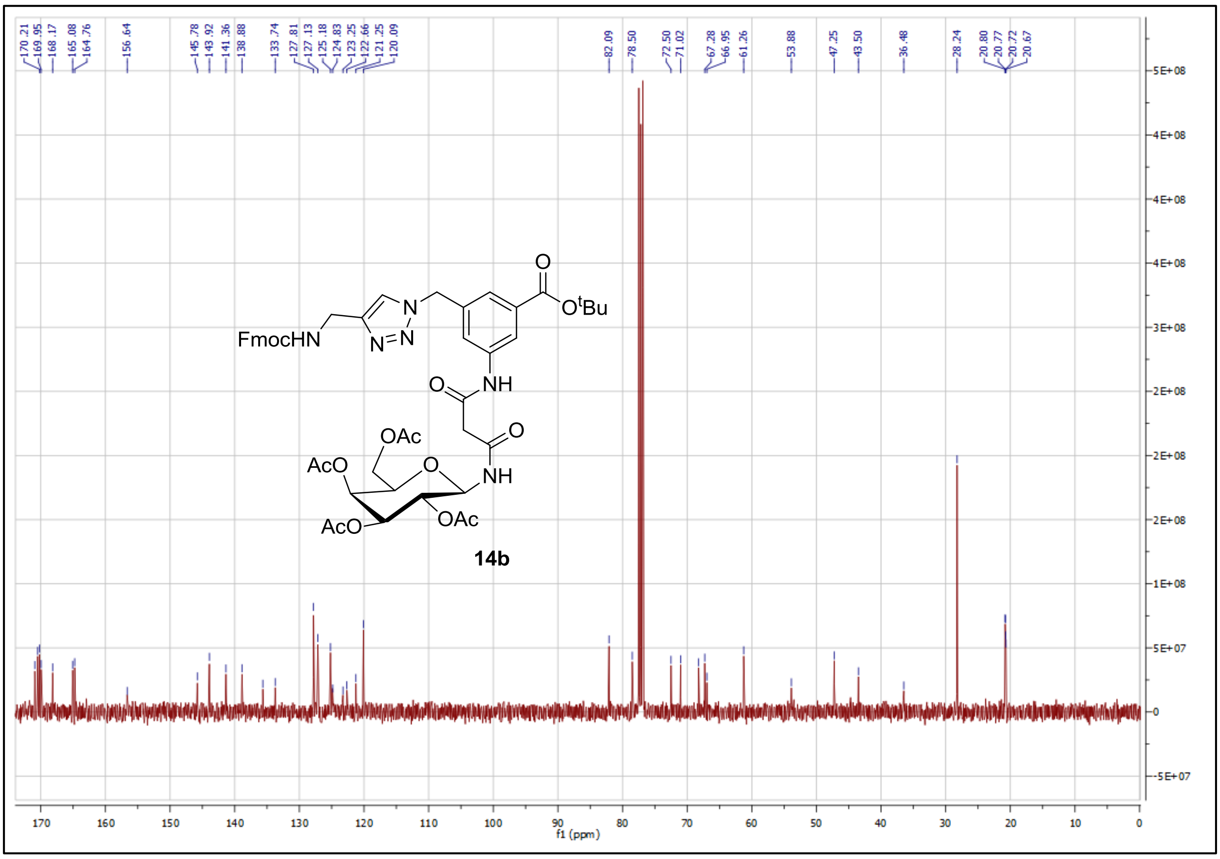
FmocHN
O
OᵗBu
NH
O
O
NH
OAc
AcO
AcO
OAc
14b
f1 (ppm)

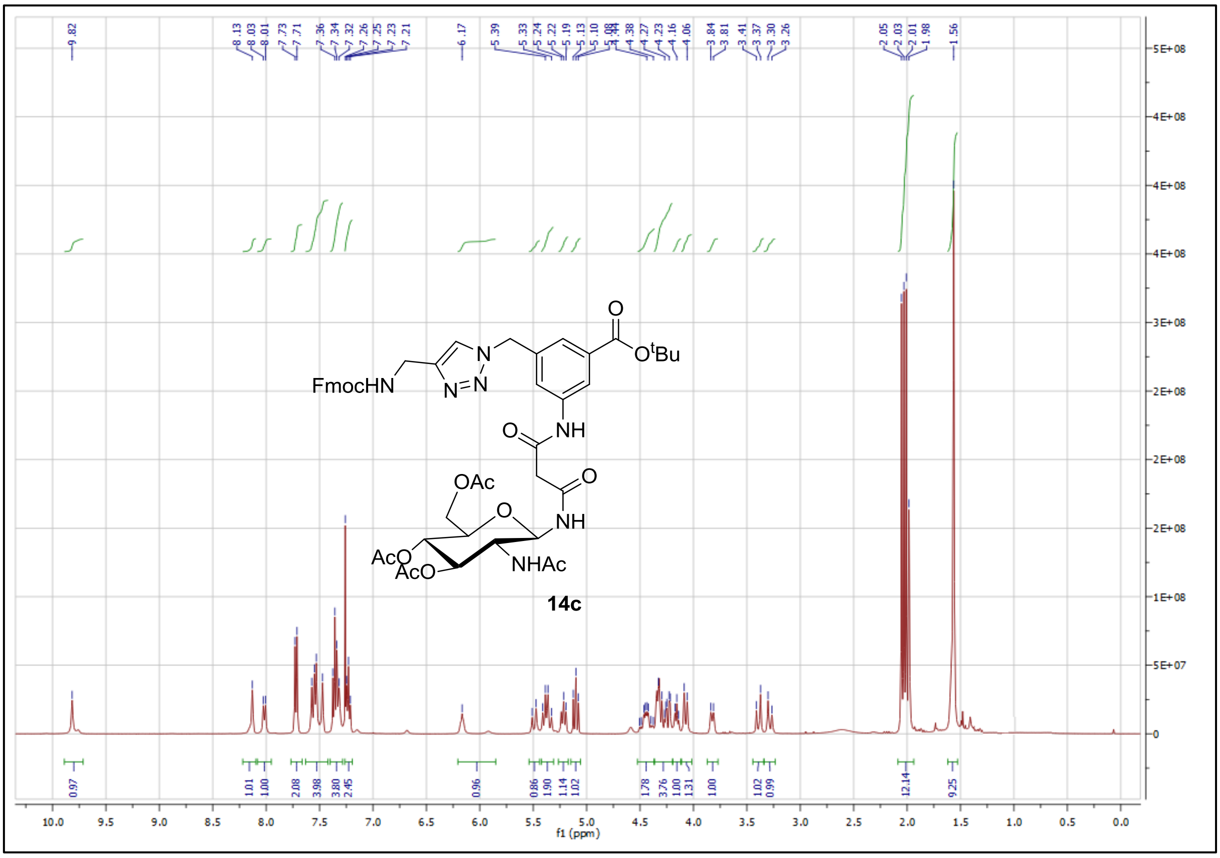

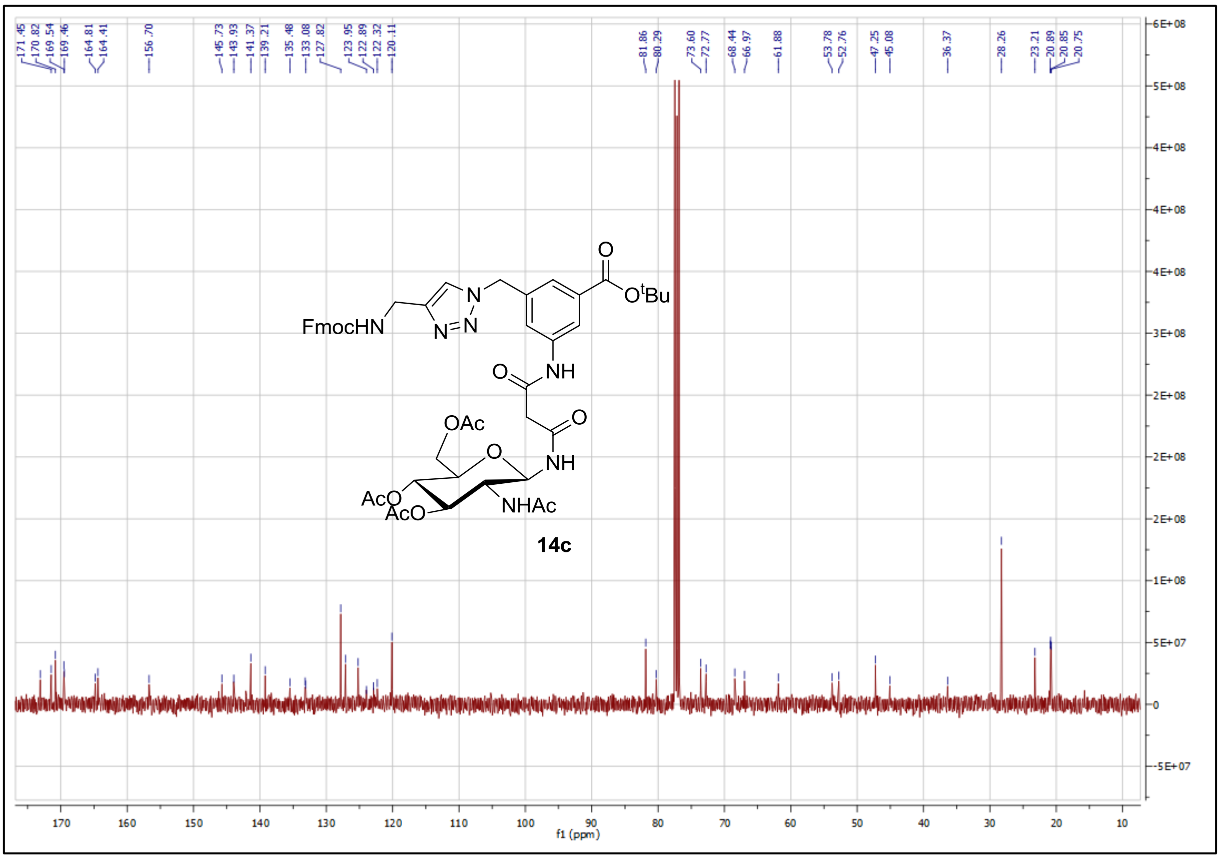

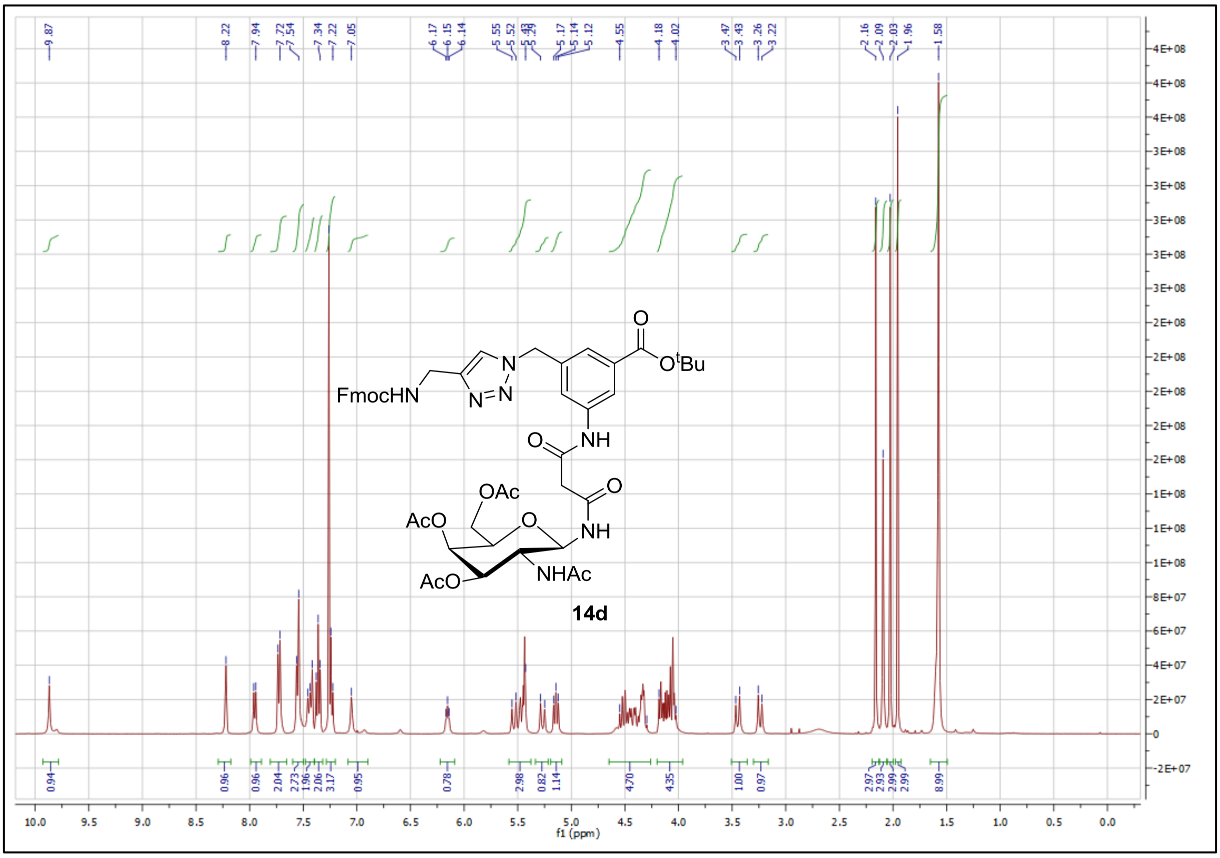
FmocHN
N
N=N
O
OtBu
NH
O
O
OAc
AcO
O
NH
AcO
NHAc
14d
f1 (ppm)

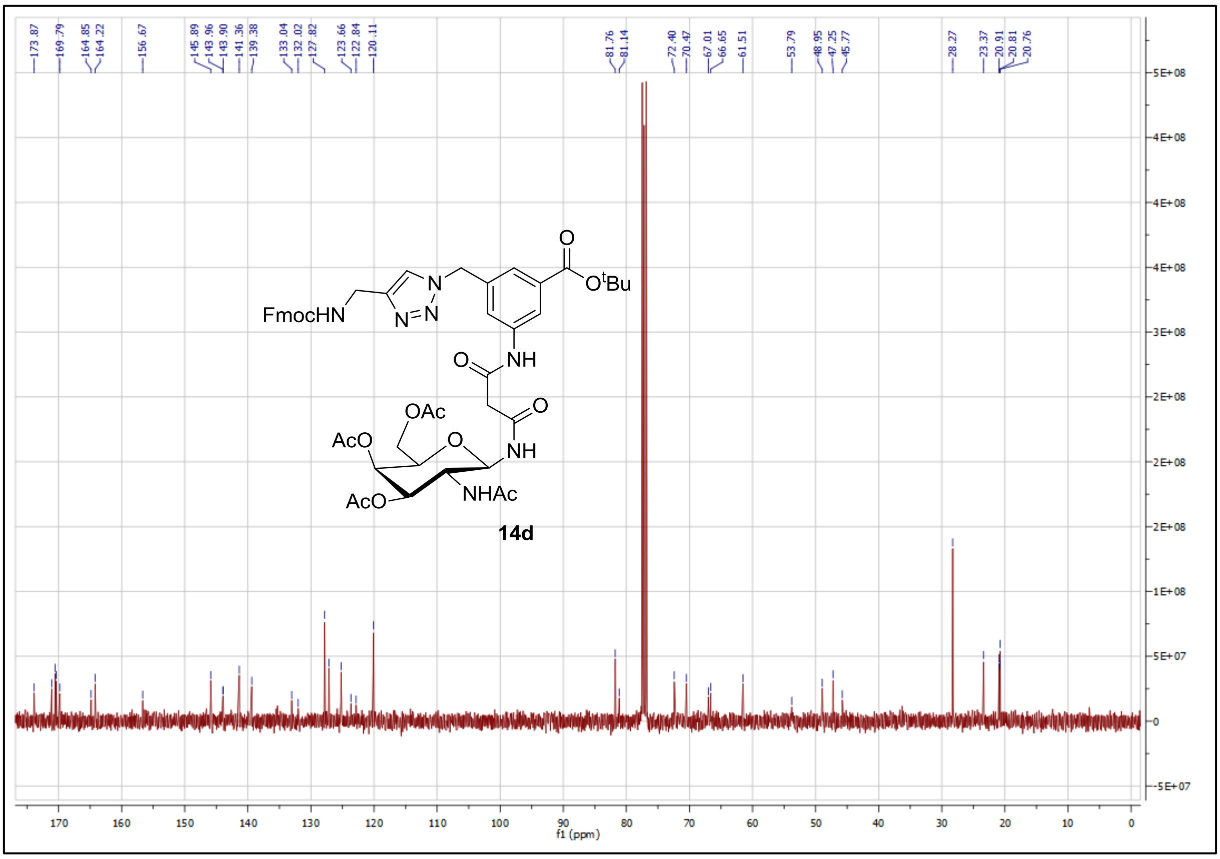
FmocHN
N
N=N
O
OtBu
NH
O
O
OAc
AcO
O
NH
AcO
NHAc
14d
f1 (ppm)

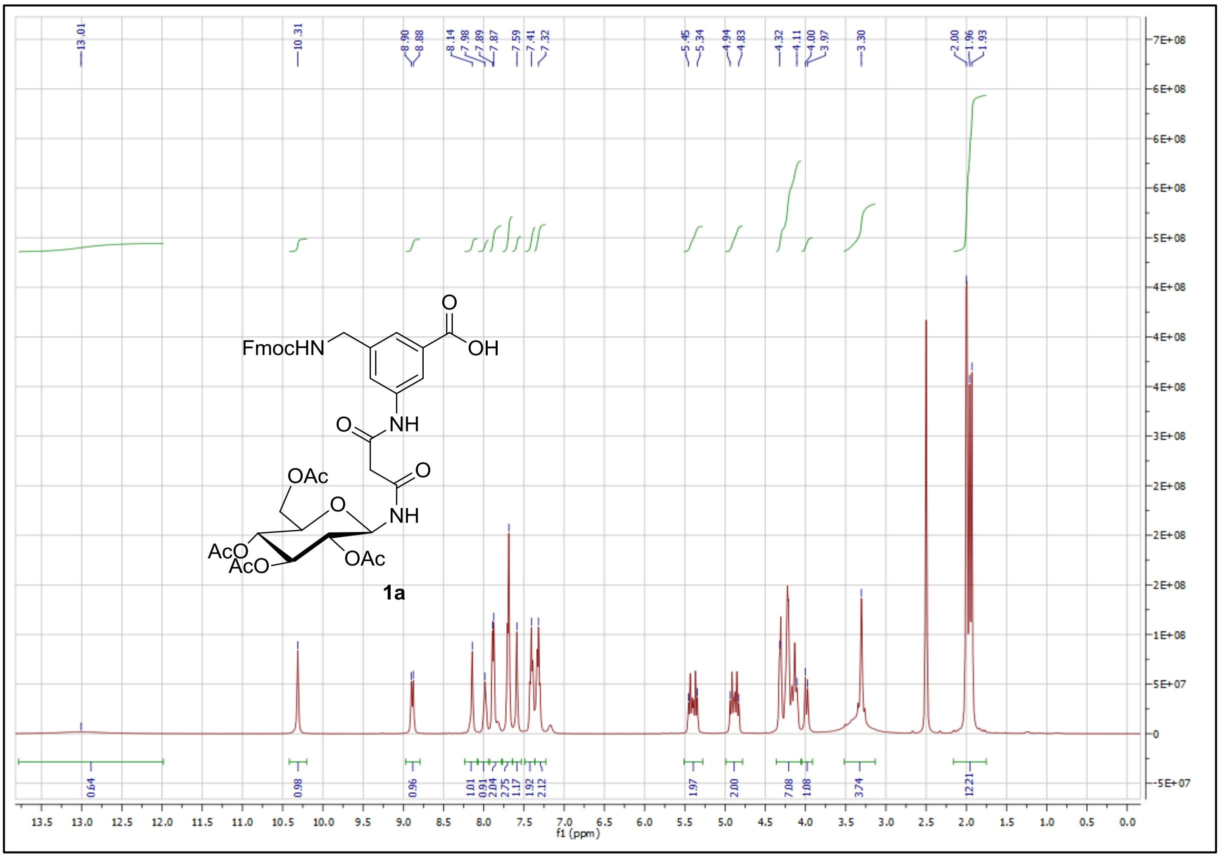

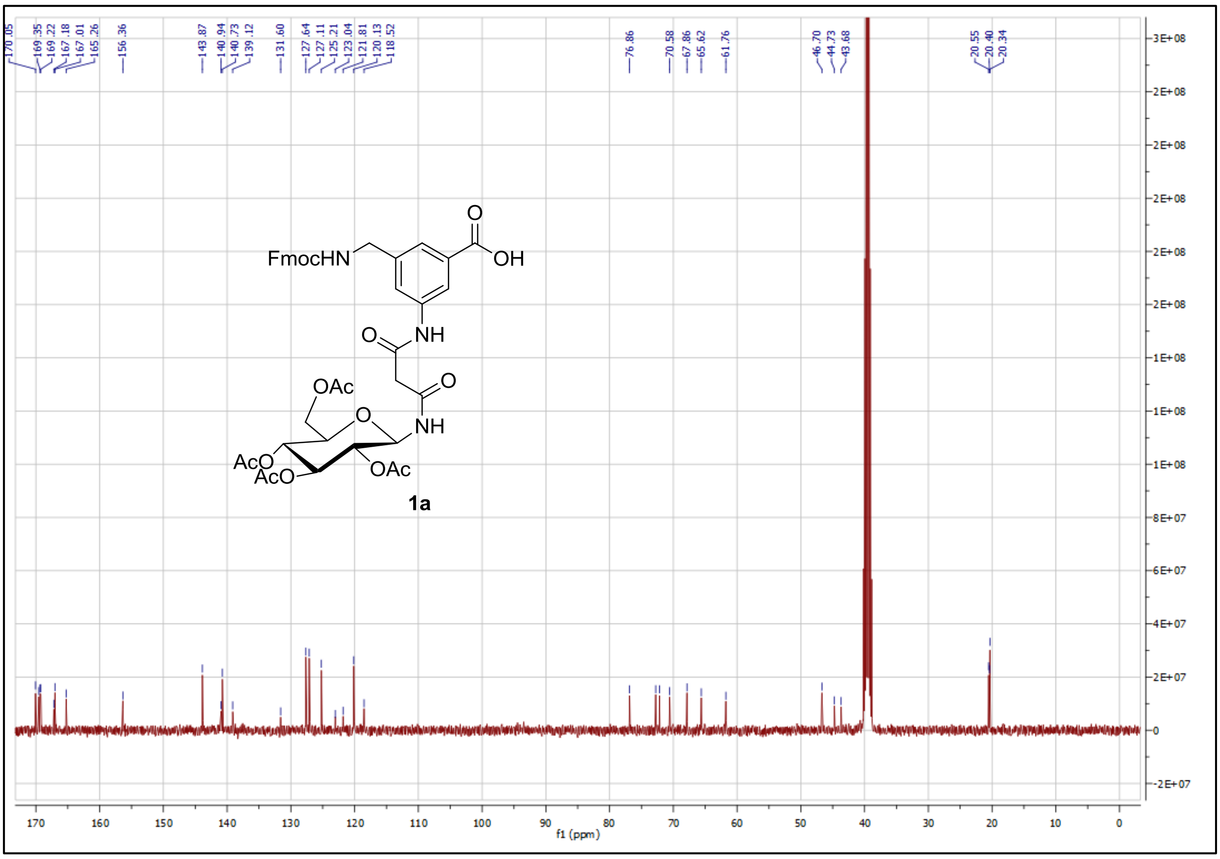

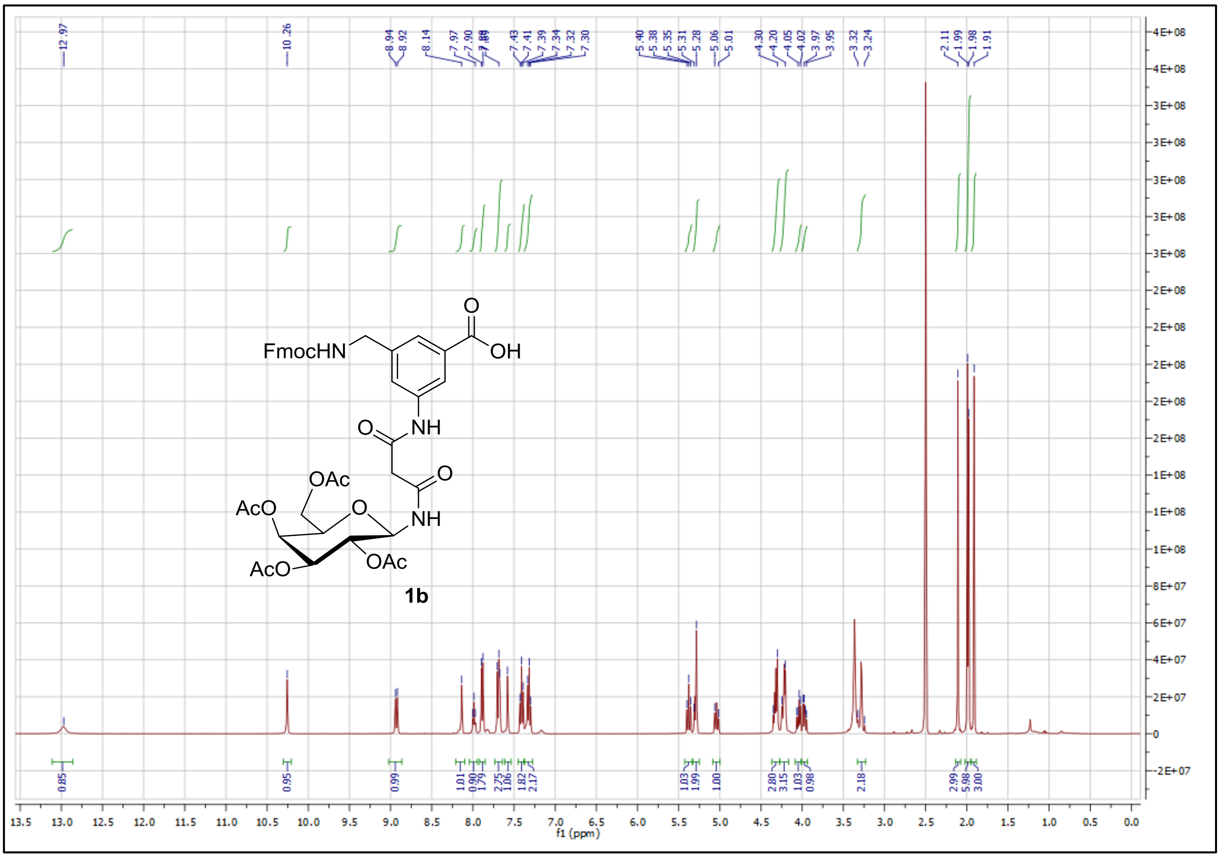
12.97
10.26
8.94
8.92
8.14
7.97
7.90
7.43
7.41
7.39
7.34
7.32
7.30
5.40
5.38
5.35
5.31
5.28
5.06
5.01
4.30
4.20
4.05
4.02
3.97
3.95
3.32
3.24
2.11
1.99
1.98
1.91
FmocHN
OH
O
NH
OAc
AcO
AcO
OAc
1b
f1 (ppm)

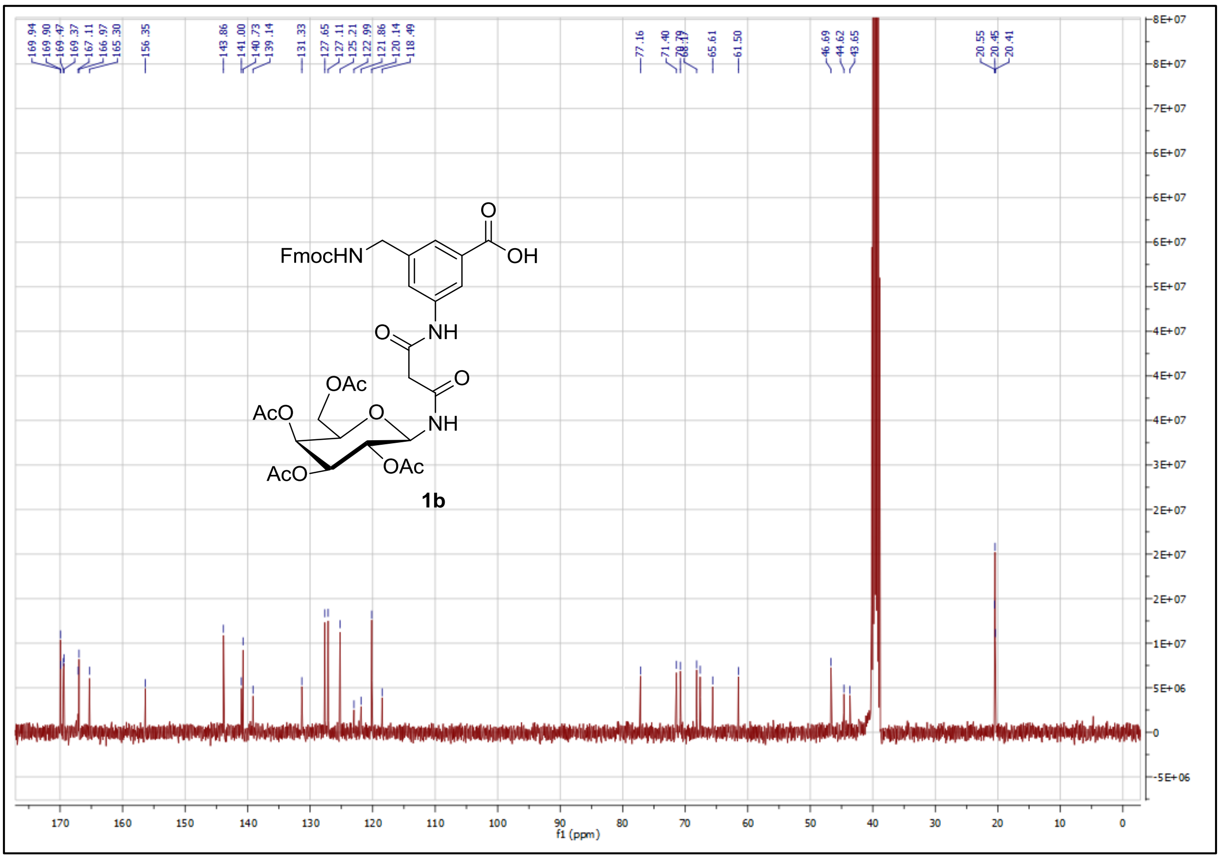
169.94
169.90
169.47
169.37
167.11
166.97
165.30
156.35
143.86
141.00
140.73
139.14
131.33
127.65
127.11
125.21
122.99
121.86
120.14
118.49
77.16
71.40
65.61
61.50
46.69
44.62
43.65
20.55
20.45
20.41
FmocHN
OH
O
NH
OAc
AcO
AcO
OAc
1b
f1 (ppm)

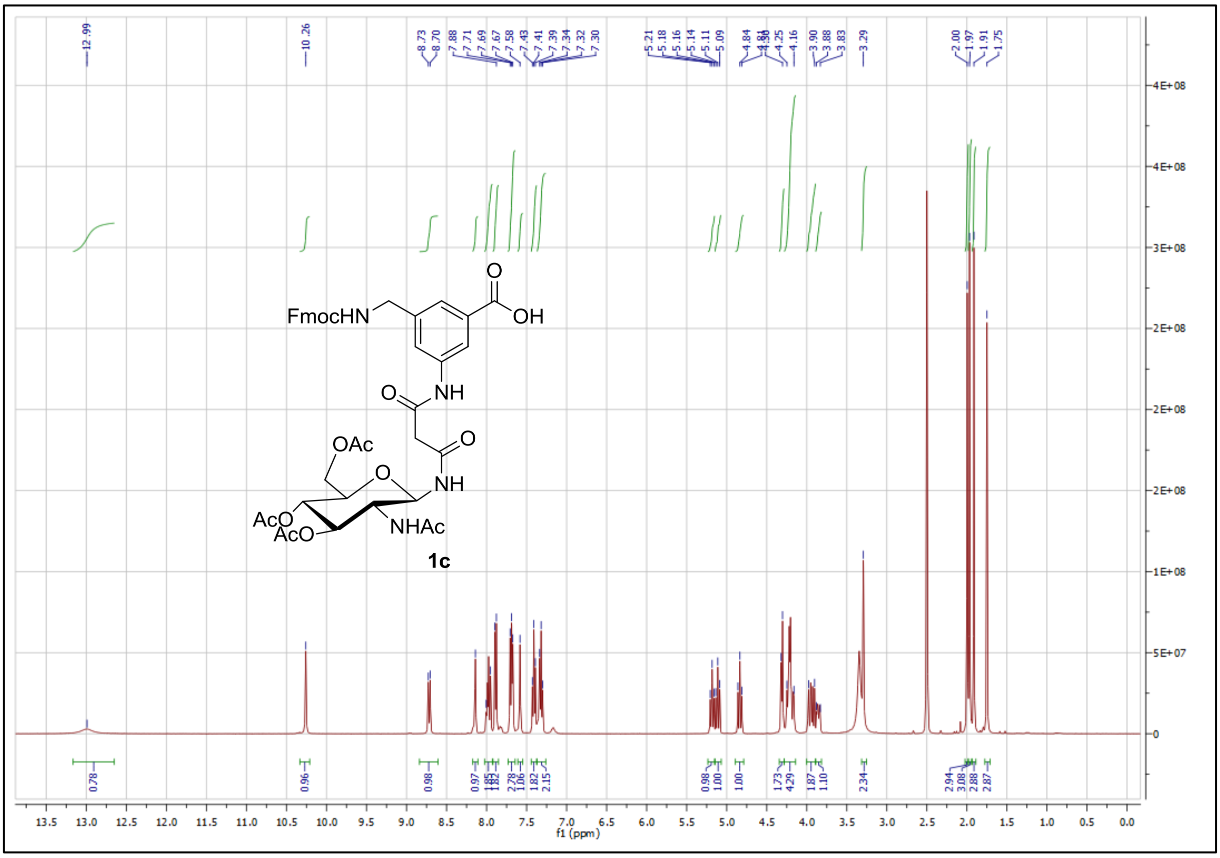
FmocHN
OH
O
NH
O
O
NH
OAc
O
AcO
AcO
NHAc
1c
f1 (ppm)

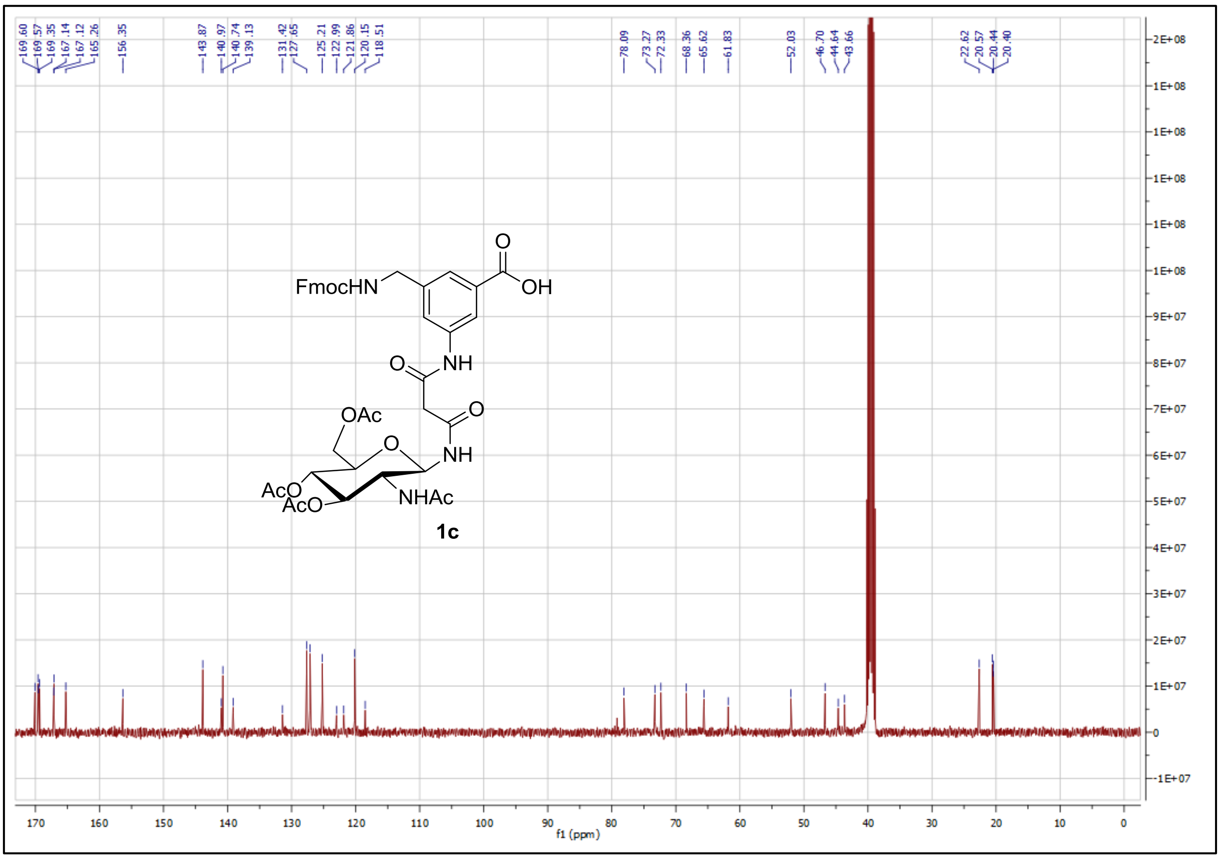
FmocHN
OH
O
NH
O
O
NH
OAc
O
AcO
AcO
NHAc
1c
f1 (ppm)

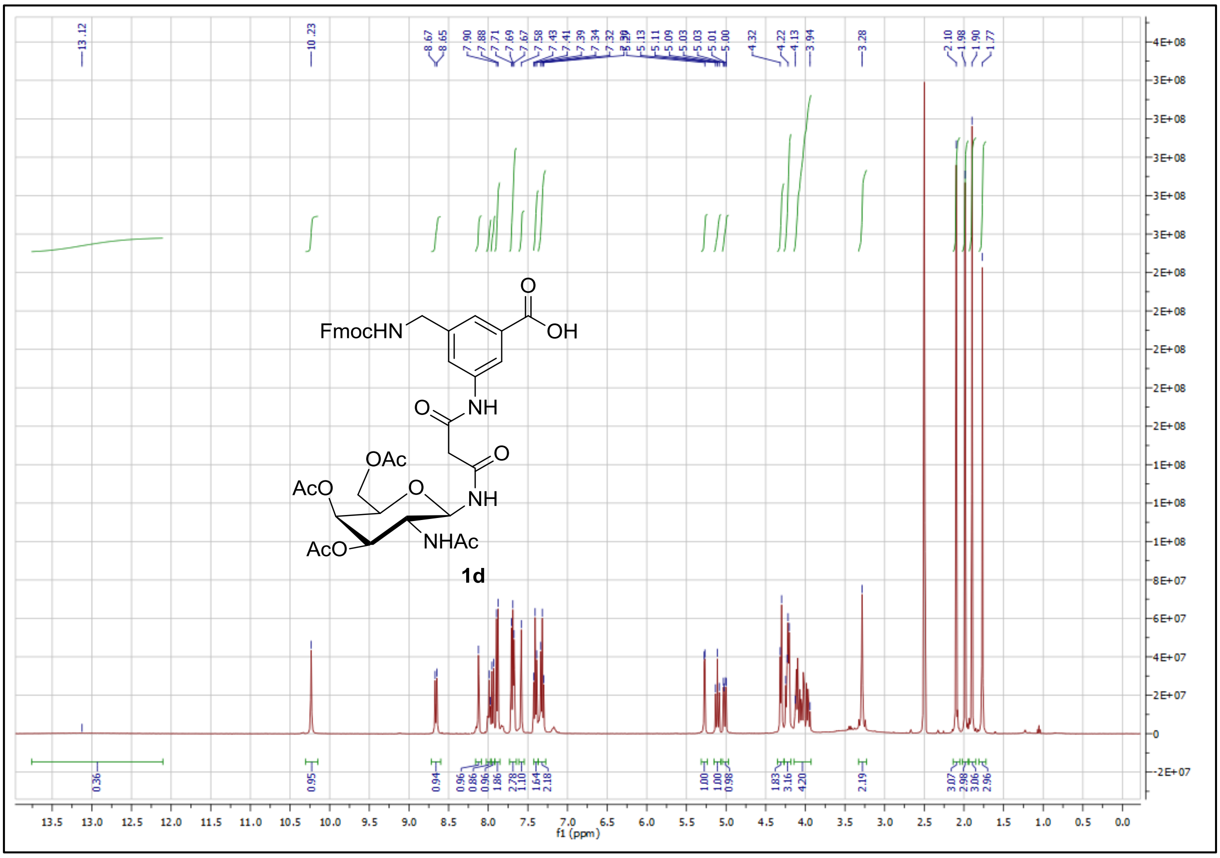

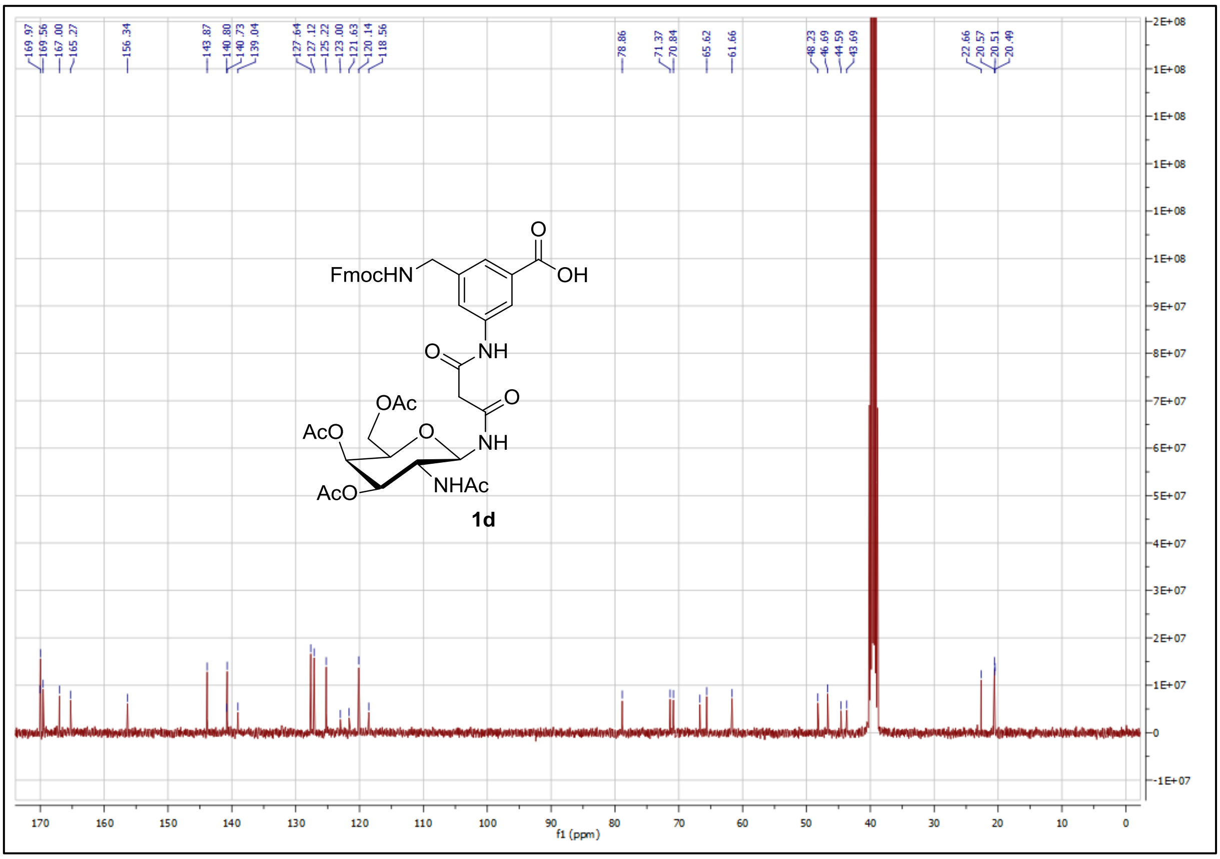

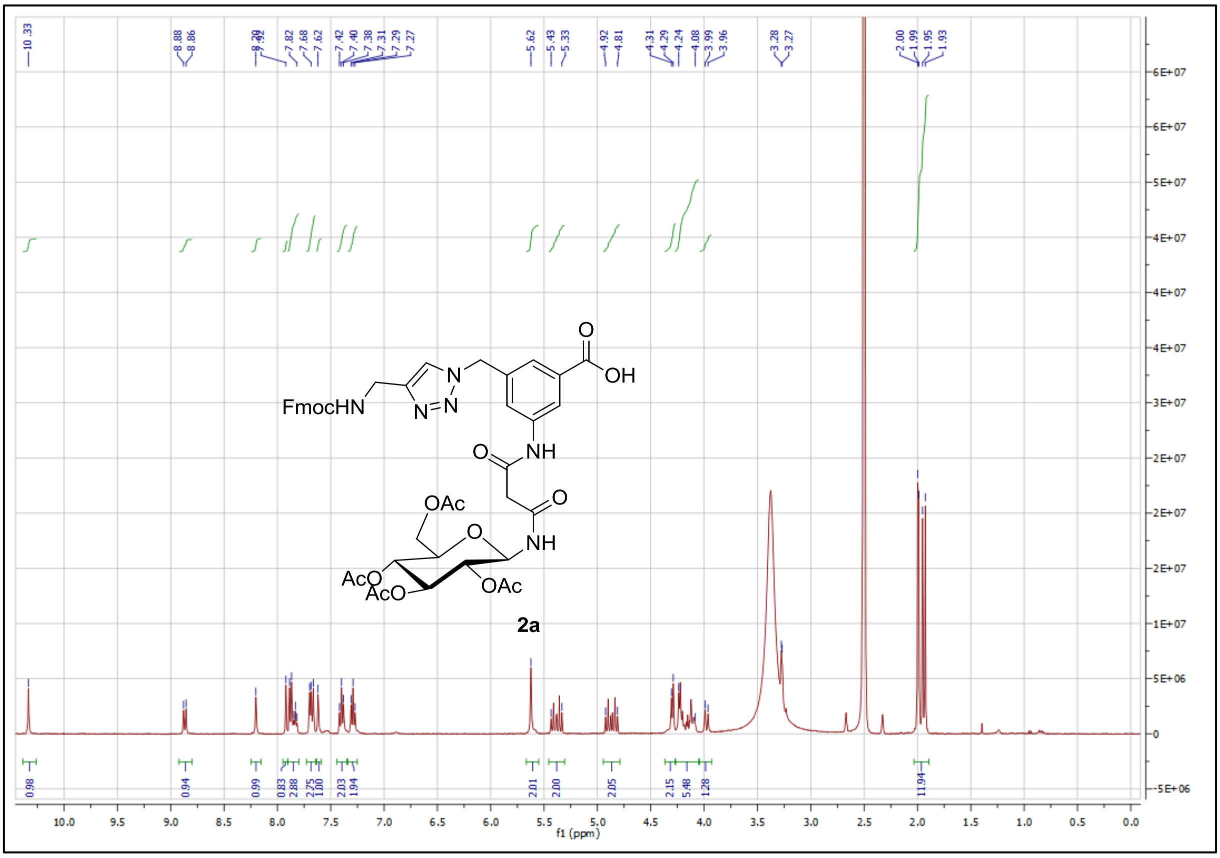

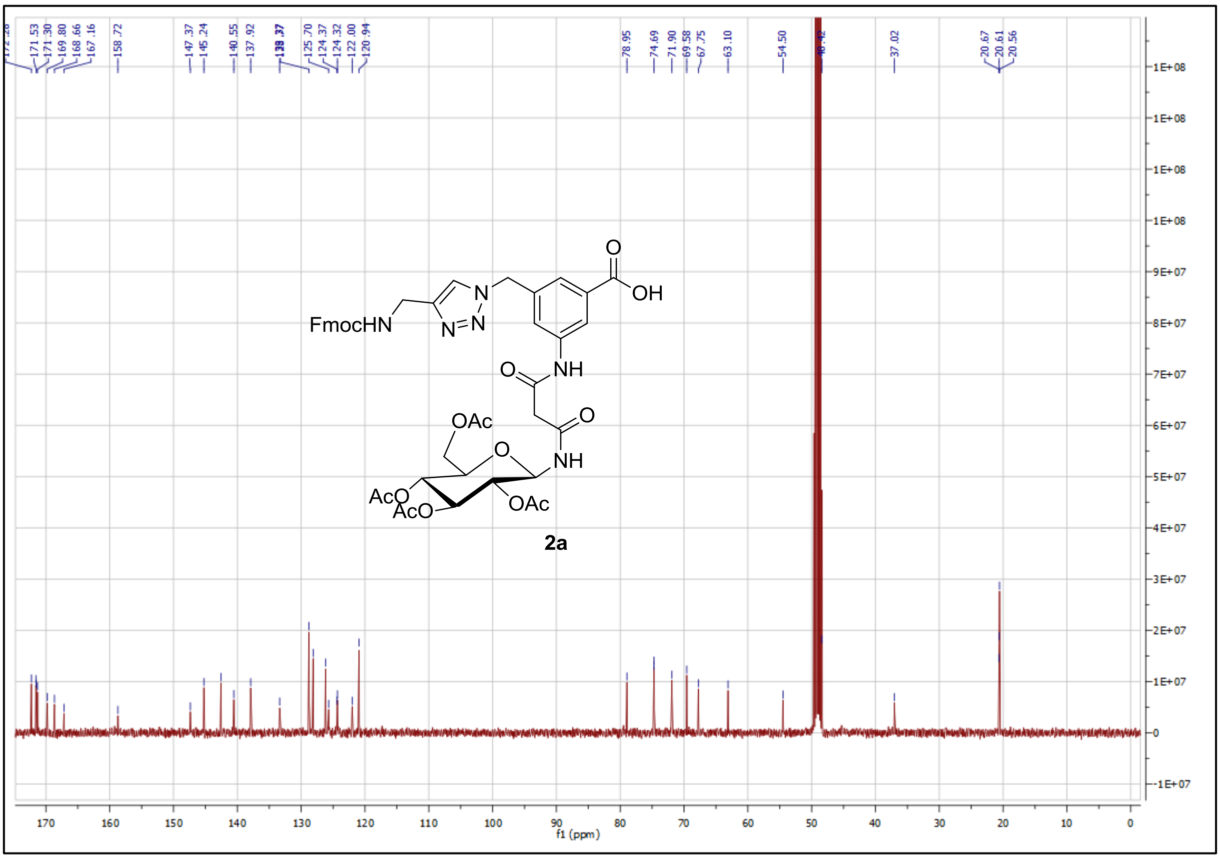

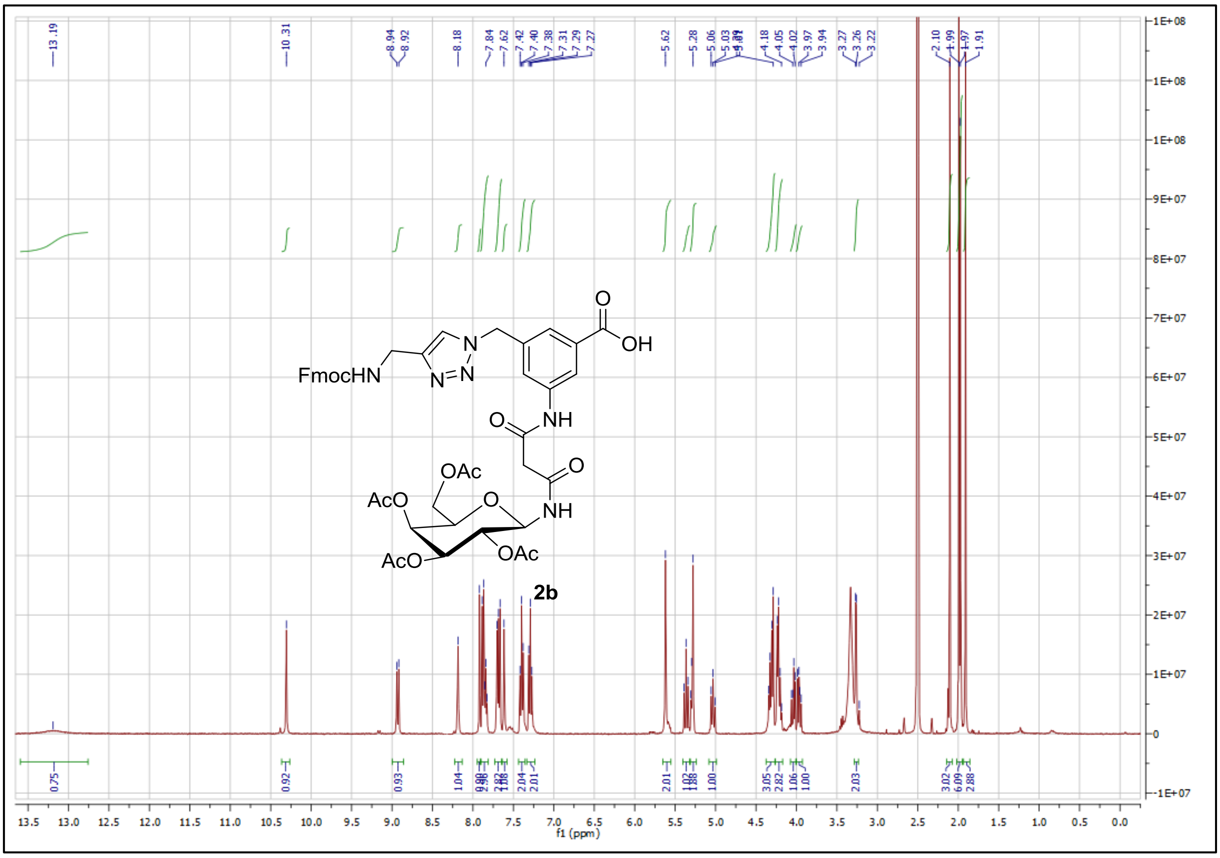

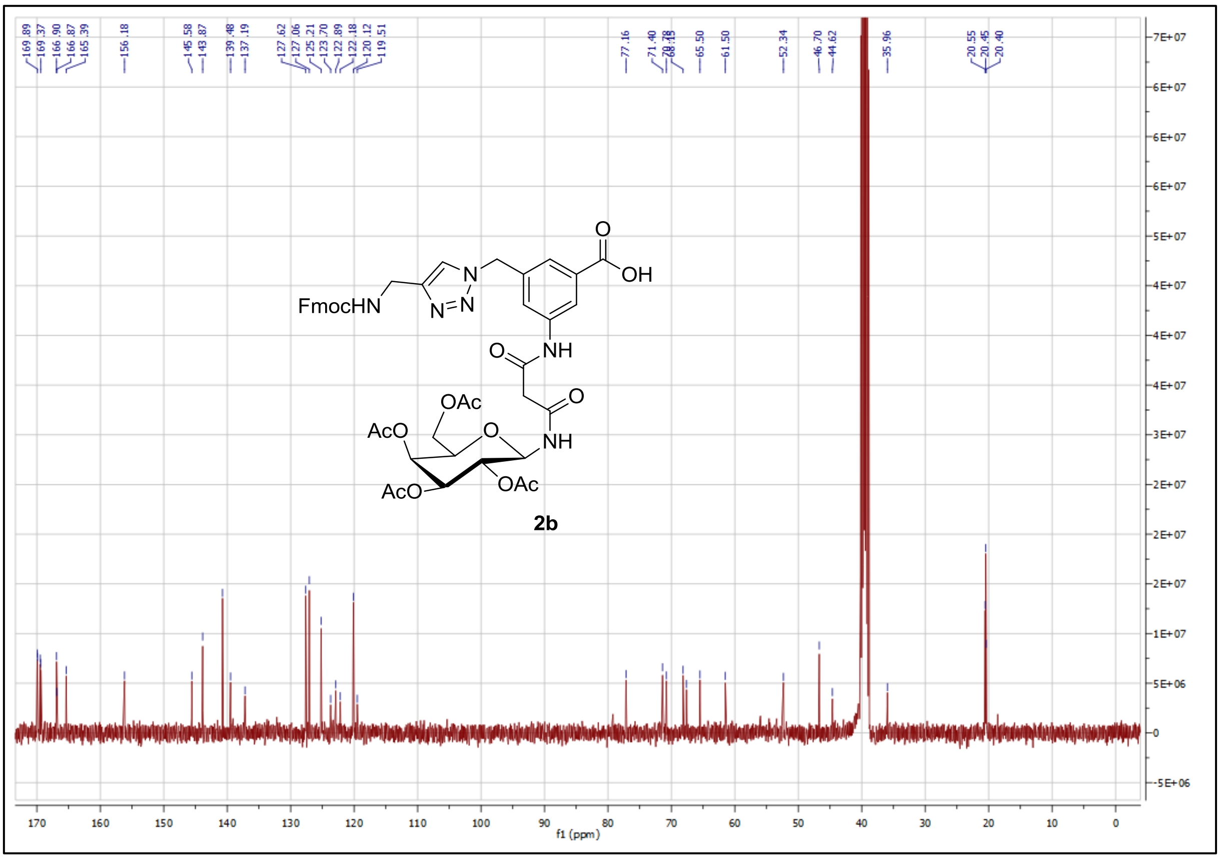

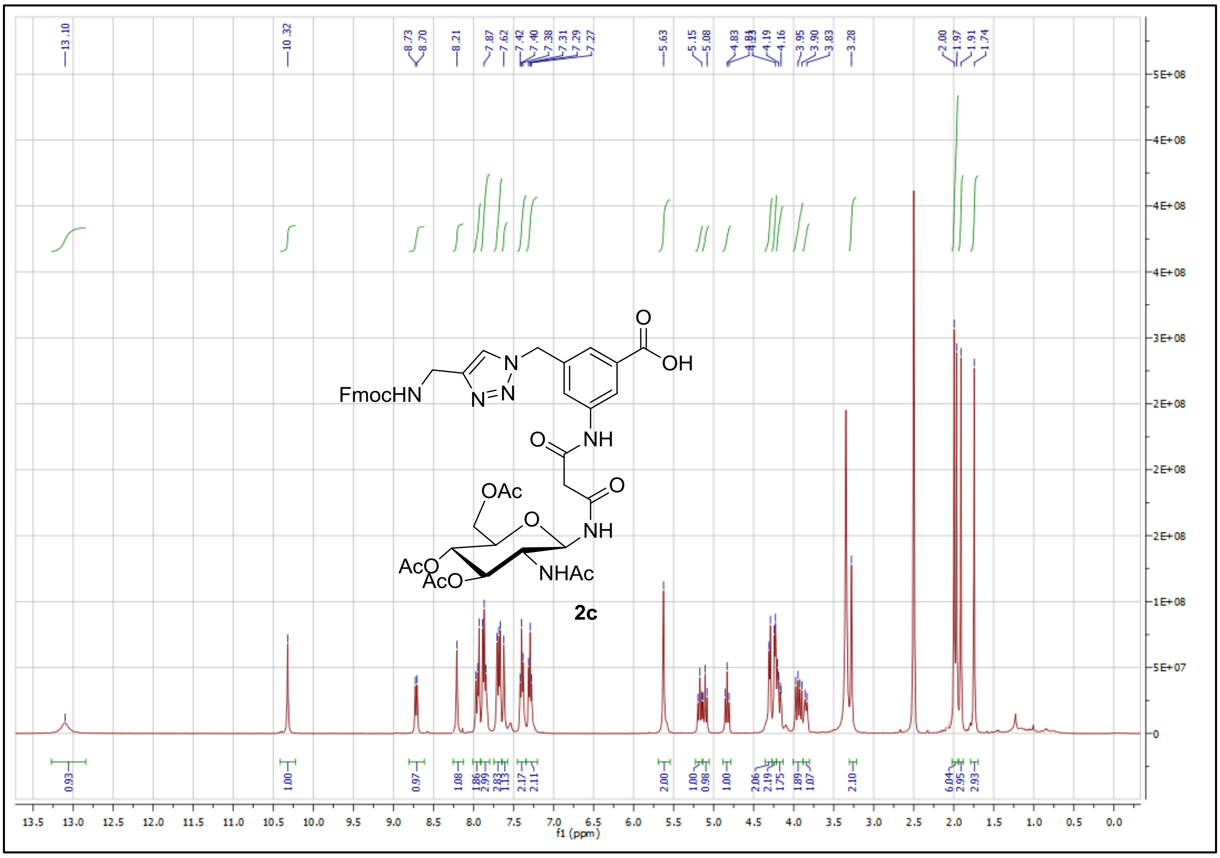

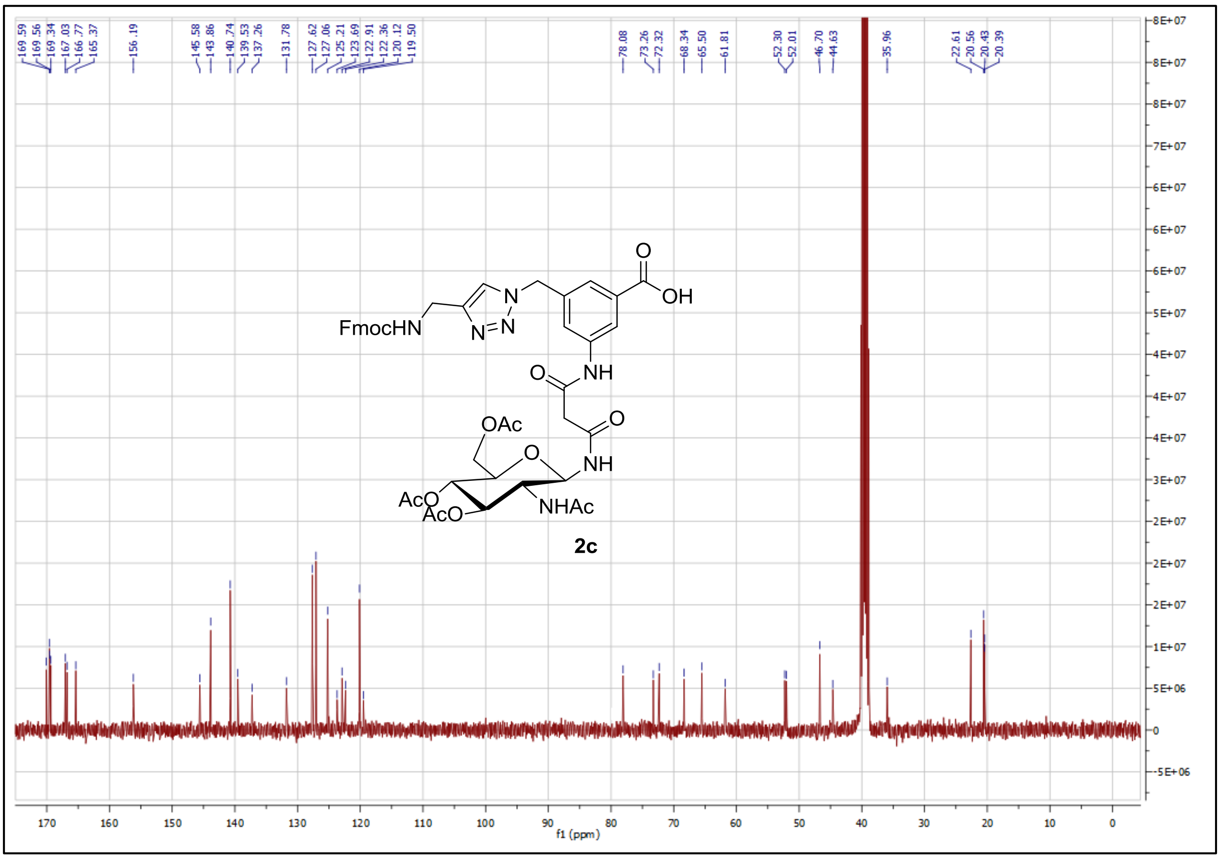

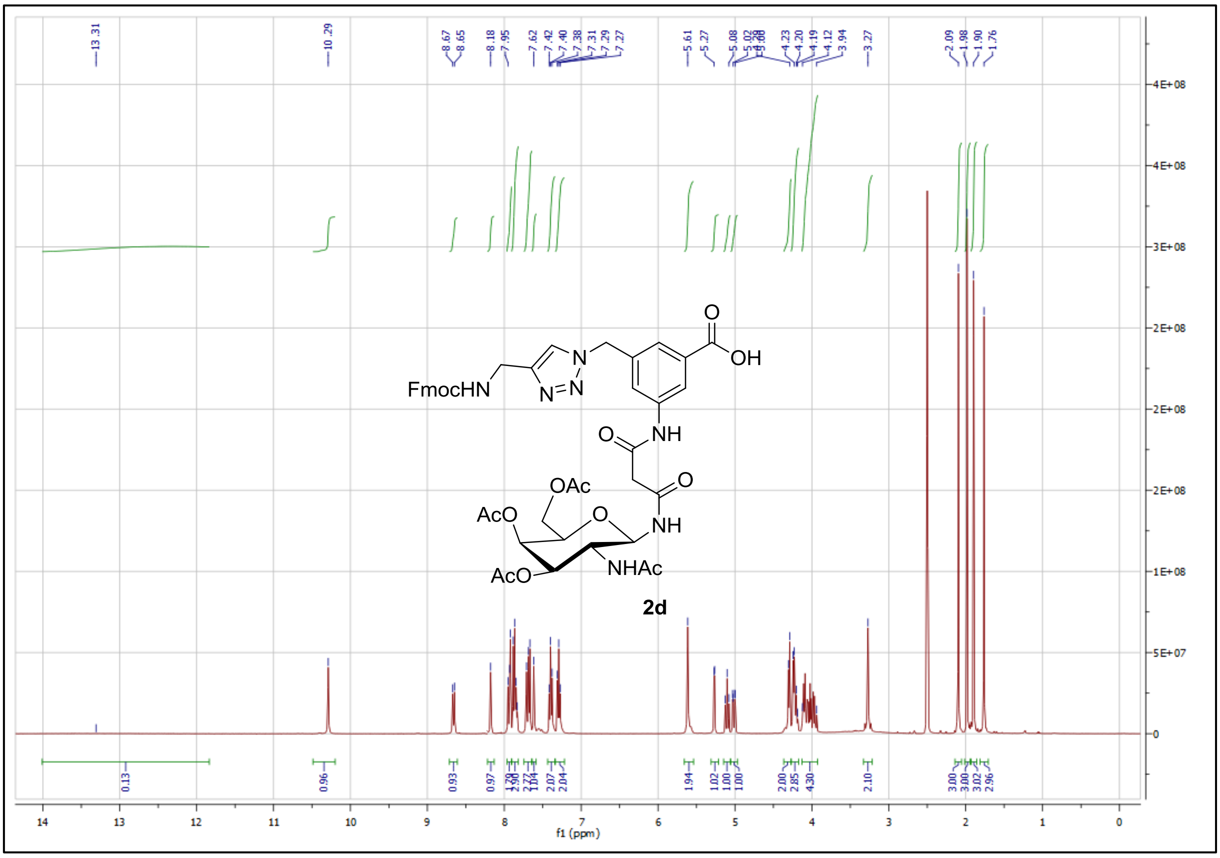
13.31
10.29
8.67
8.65
8.18
7.95
7.62
7.42
7.40
7.38
7.31
7.29
7.27
5.61
5.27
5.08
5.02
4.23
4.20
4.19
4.12
3.94
3.27
2.09
1.98
1.90
1.76
FmocHN
N
N=N
O
OH
NH
O
O
OAc
AcO
O
NH
AcO
NHAc
2d
0.13
0.96
0.93
0.97
2.07
2.04
1.94
1.02
1.00
1.00
2.00
2.85
4.30
2.10
3.00
3.00
3.02
2.96
f1 (ppm)

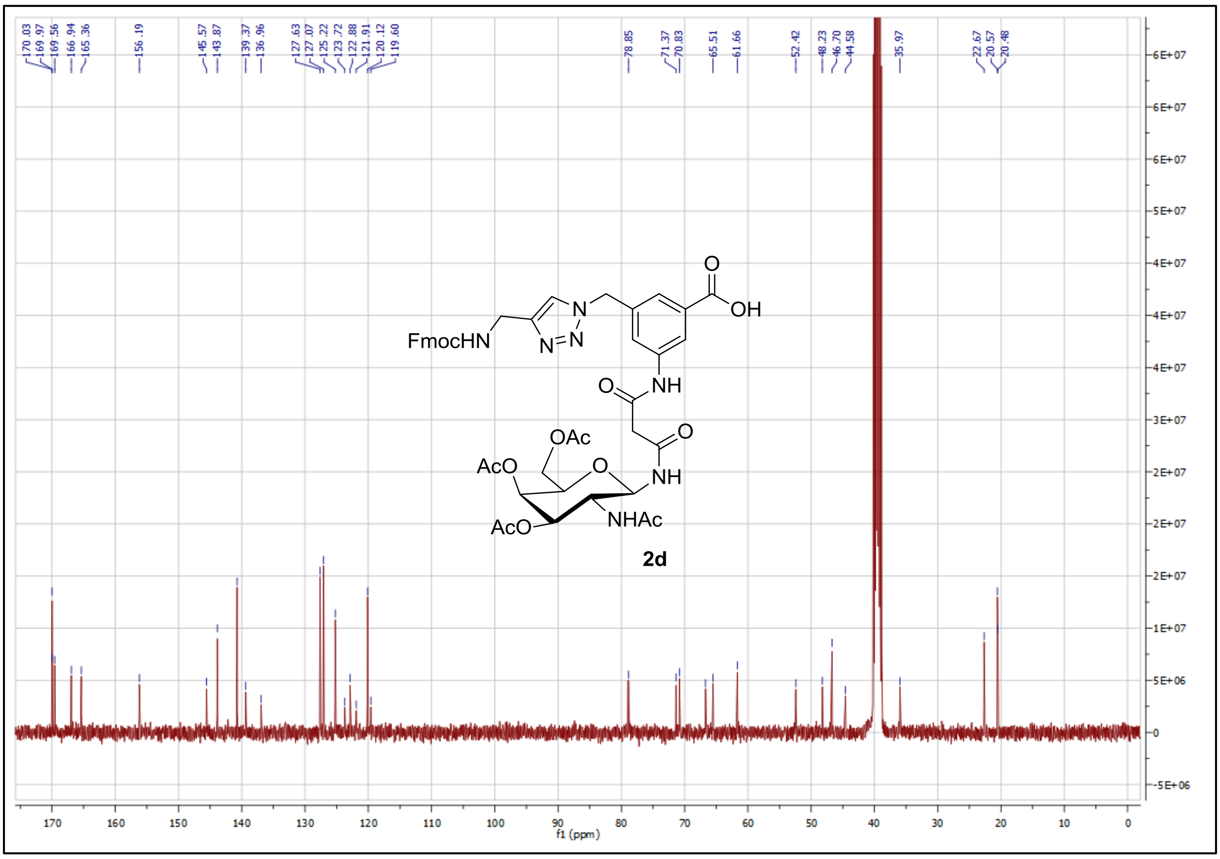
170.03
169.97
169.56
166.94
165.36
156.19
145.57
143.87
139.37
136.96
127.63
127.07
125.22
123.72
122.88
121.91
120.12
119.60
78.85
71.37
70.83
65.51
61.66
52.42
48.23
46.70
44.58
35.97
22.67
20.57
20.48
FmocHN
N
N=N
O
OH
NH
O
O
OAc
AcO
O
NH
AcO
NHAc
2d
f1 (ppm)

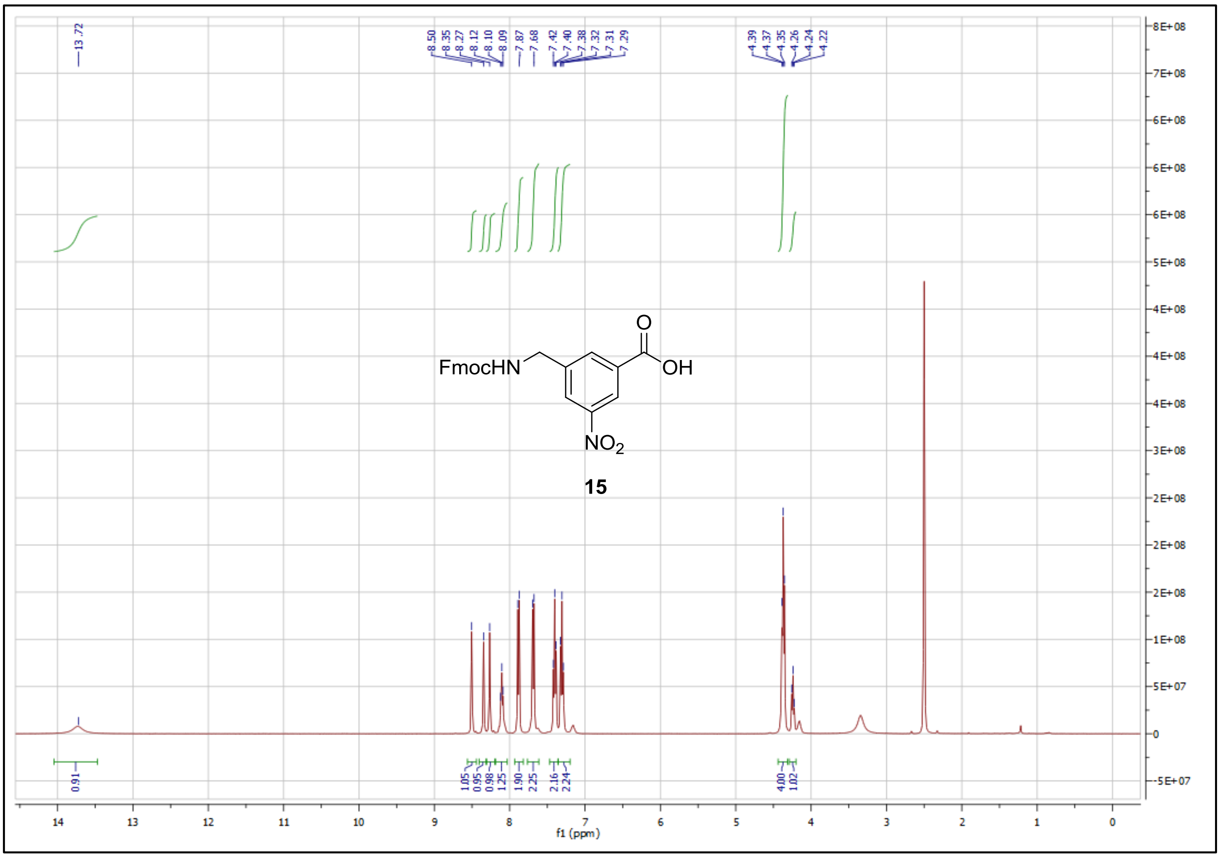

13.72
8.50
8.35
8.27
8.12
8.10
8.09
7.87
7.68
7.42
7.40
7.38
7.32
7.31
7.29
4.39
4.37
4.35
4.26
4.24
4.22
FmocHN
O
OH
NO2
15
0.91
1.05
0.95
0.98
1.25
1.90
2.25
2.16
2.24
4.00
1.02
f1 (ppm)

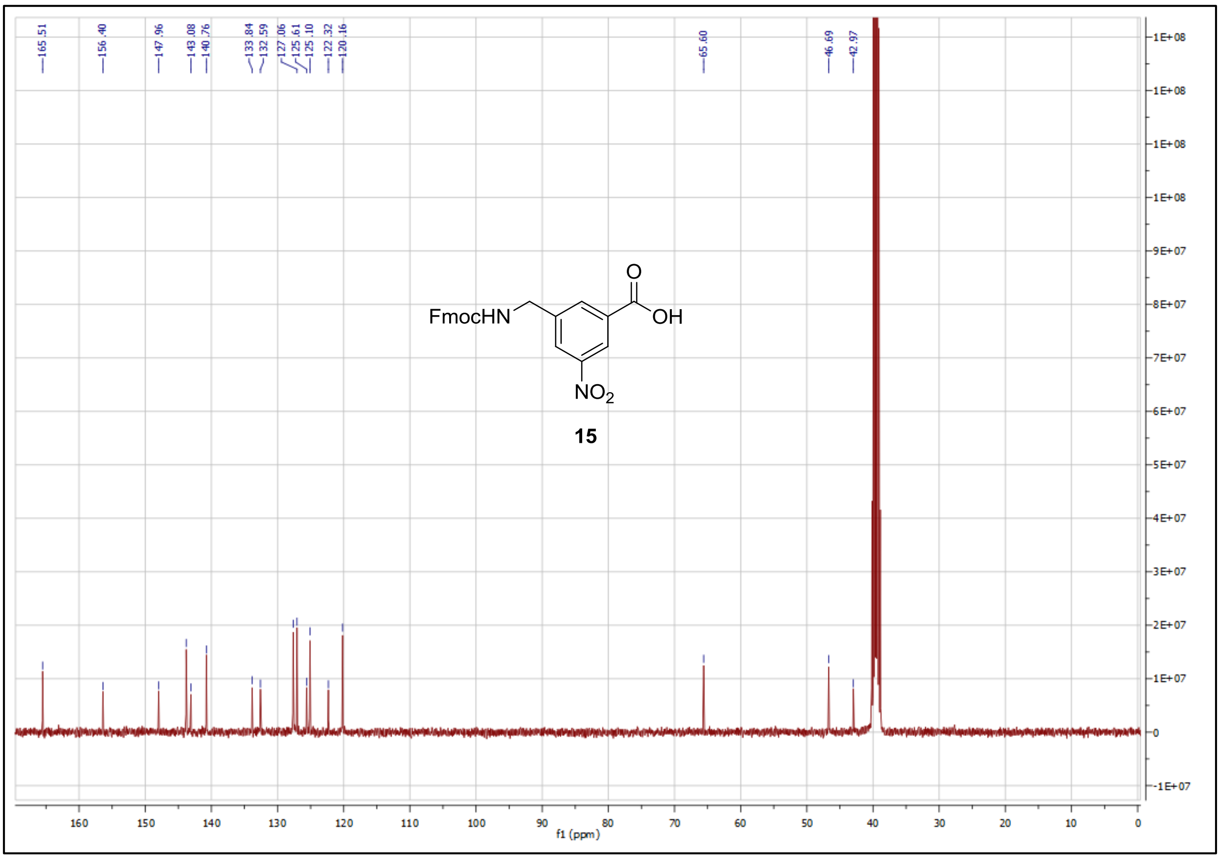

165.51
156.40
147.96
143.08
140.76
133.84
132.59
127.06
125.61
125.10
122.32
120.16
65.60
46.69
42.97
FmocHN
O
OH
NO2
15
f1 (ppm)

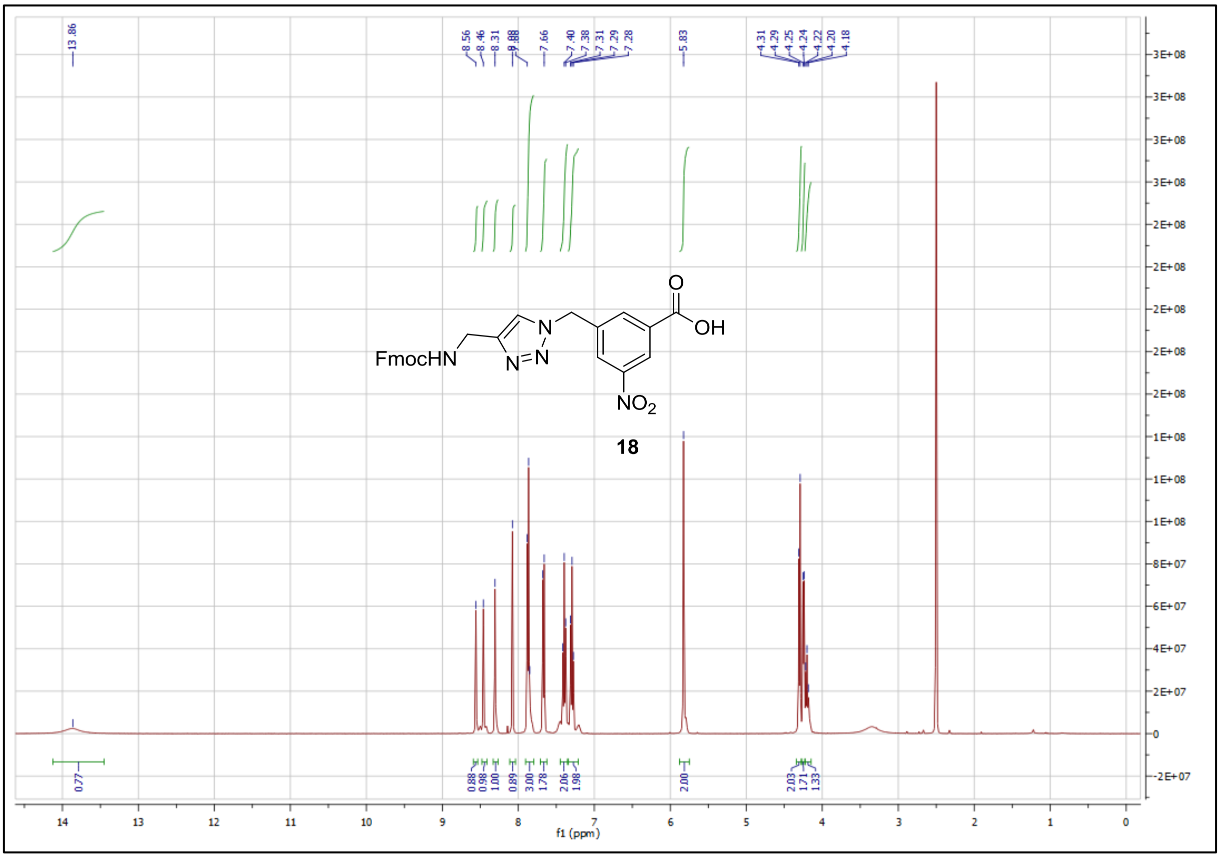

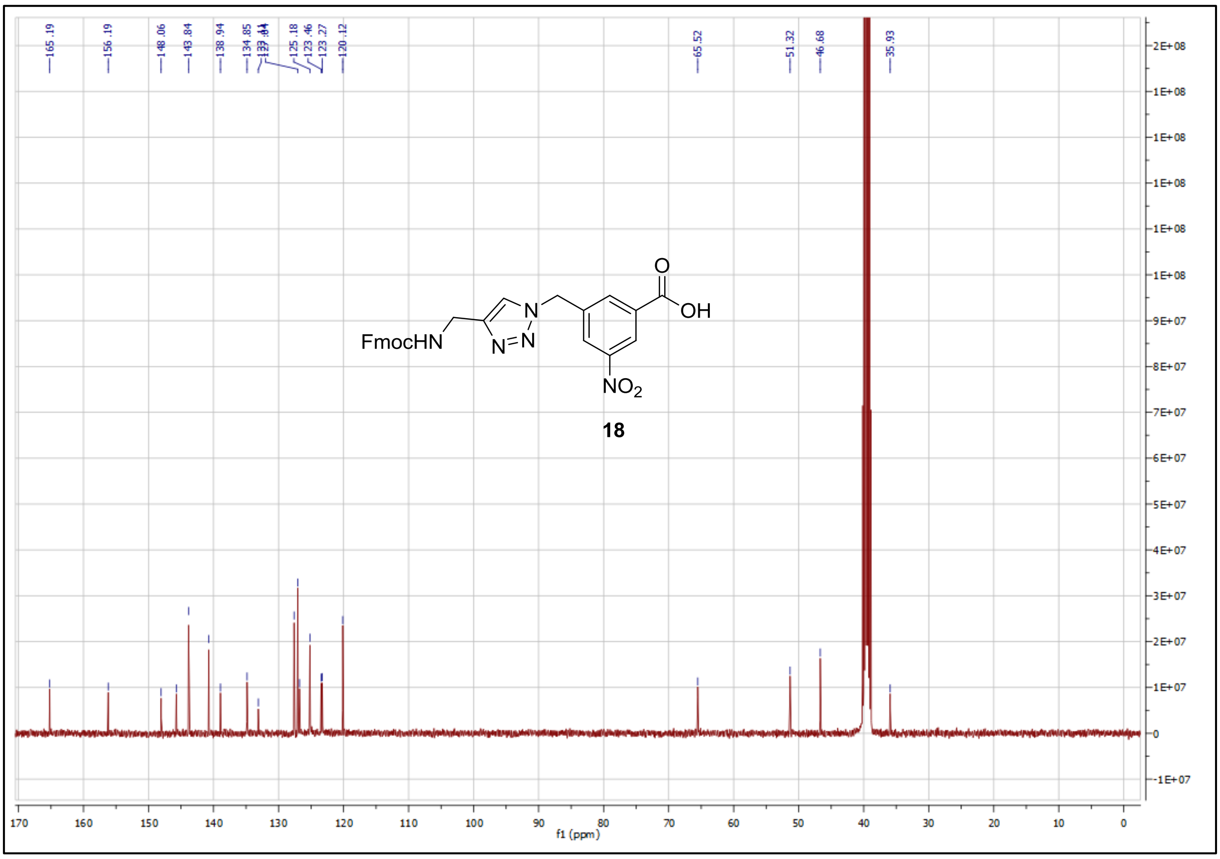

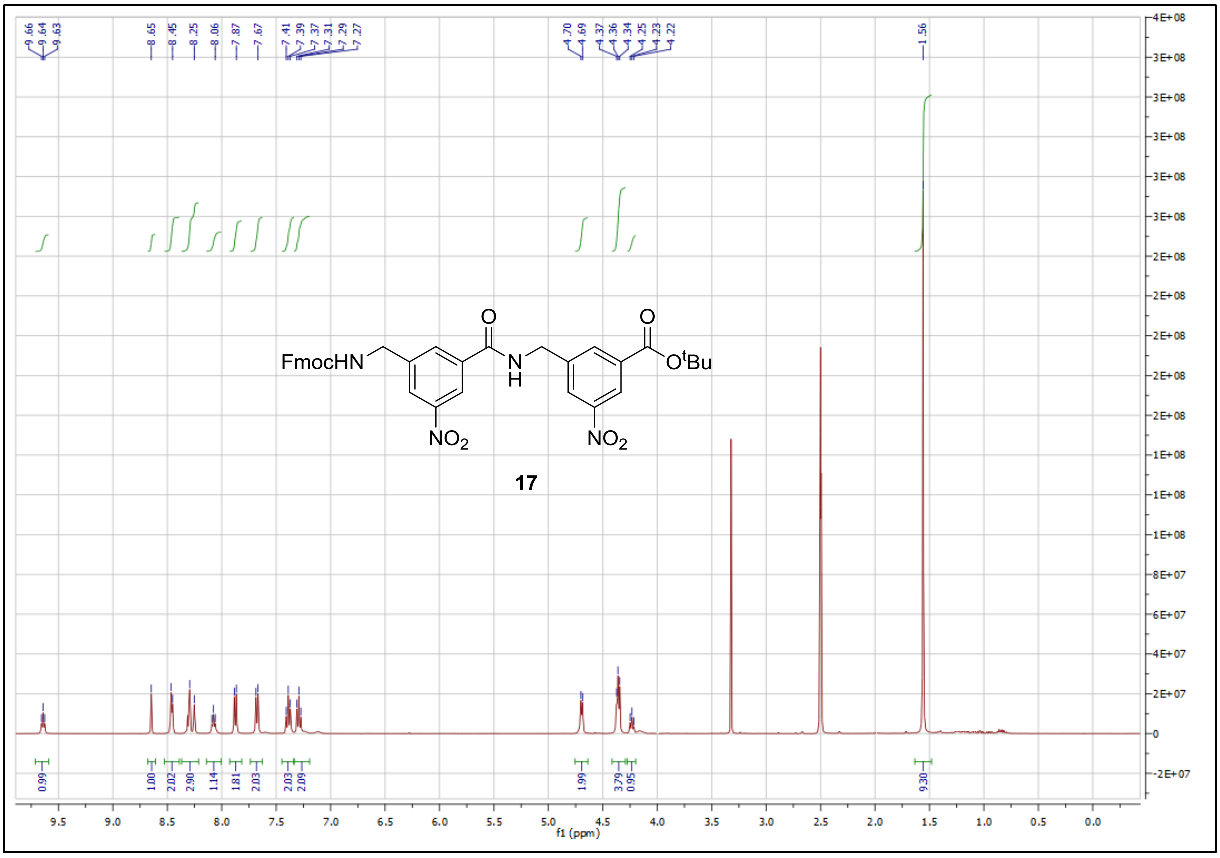
FmocHN
NO2
NO2
OtBu
17
f1 (ppm)

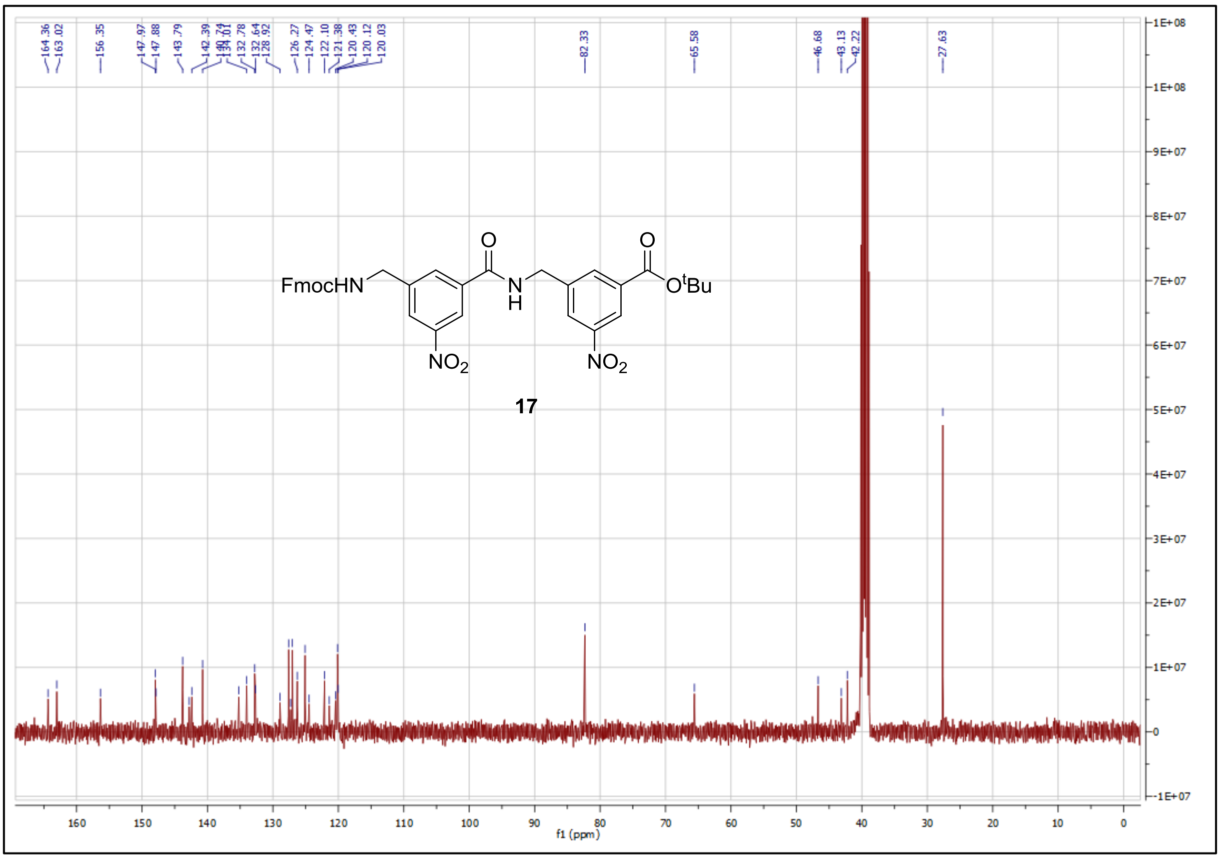
FmocHN
NO2
NO2
OtBu
17
f1 (ppm)

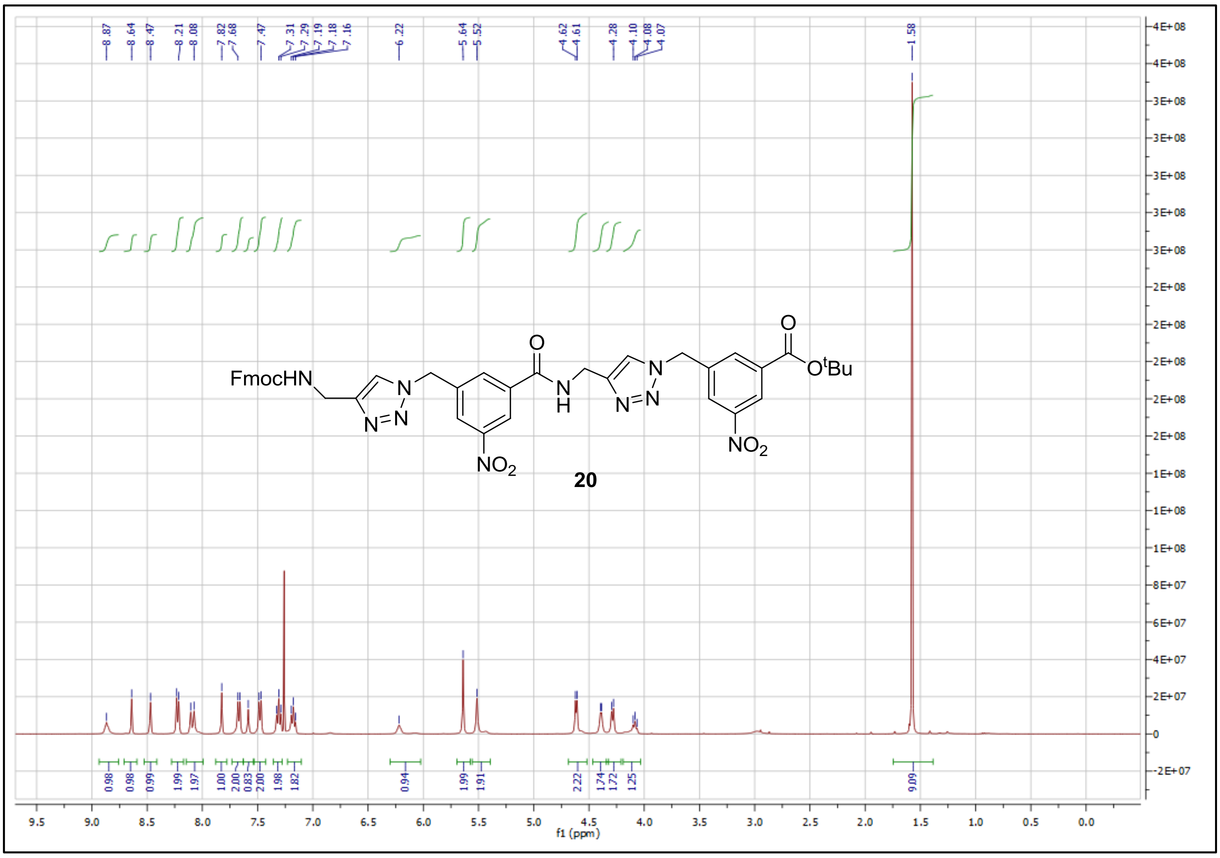
FmocHN
NO2
20
OtBu
f1 (ppm)

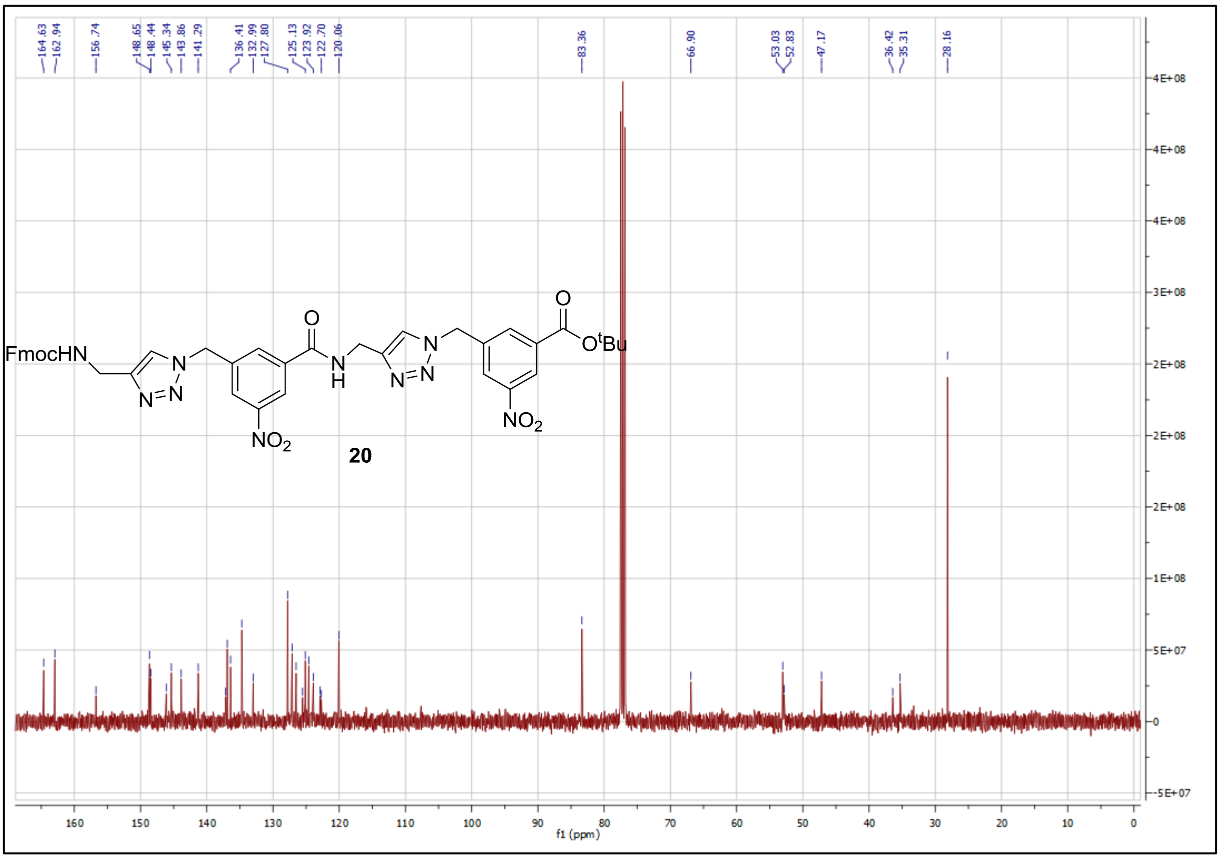
FmocHN
NO2
20
OtBu
f1 (ppm)

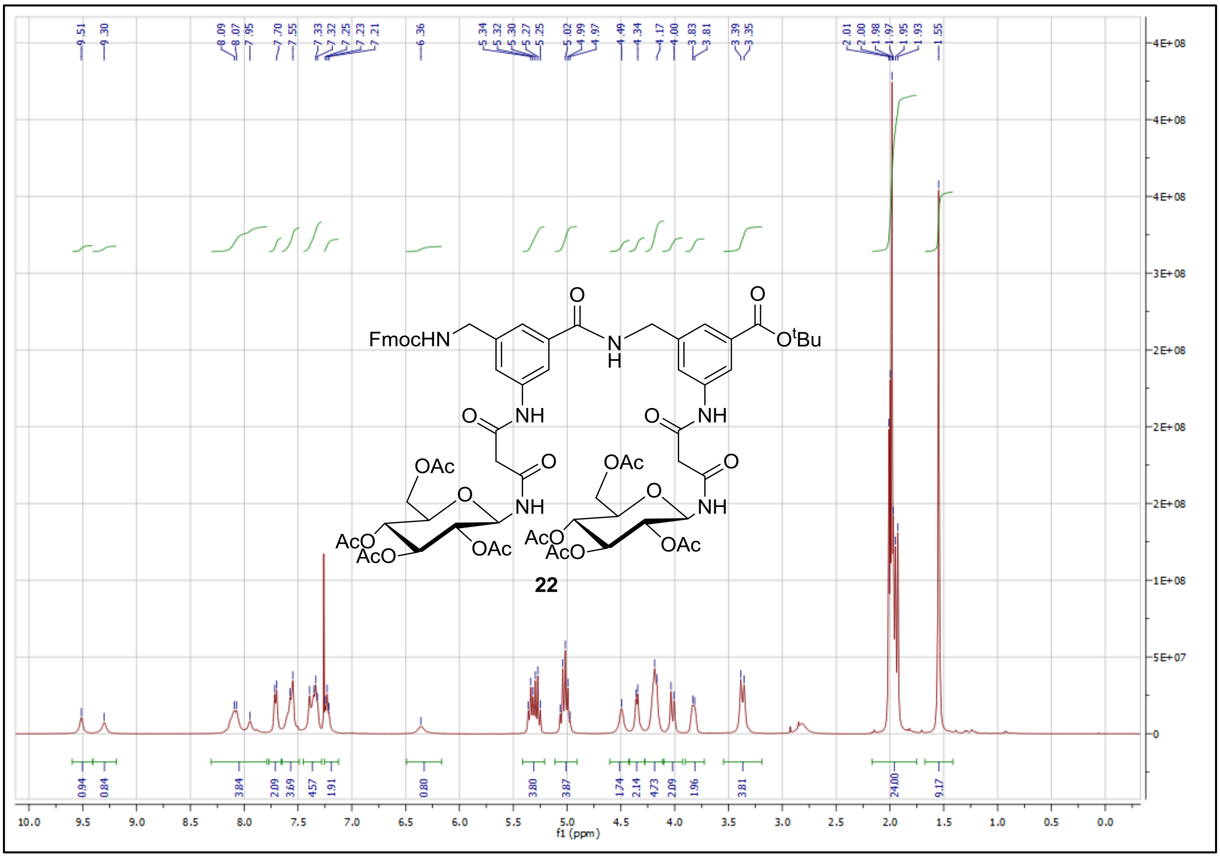
9.51
9.30
8.09
8.07
7.95
7.70
7.55
7.33
7.32
7.25
7.23
7.21
6.36
5.34
5.32
5.30
5.27
5.25
5.02
4.99
4.97
4.49
4.34
4.17
4.00
3.83
3.81
3.39
3.35
2.01
2.00
1.98
1.97
1.95
1.93
1.55
FmocHN
O
N
H
O
O^{t}Bu
NH
O
NH
O
O
O
OAc
O
NH
AcO
AcO
OAc
OAc
O
NH
AcO
AcO
OAc
22
0.94
0.84
3.84
2.09
3.69
4.57
1.91
0.80
3.80
3.87
1.74
2.14
4.73
2.09
1.96
3.81
24.00
9.17
f1 (ppm)

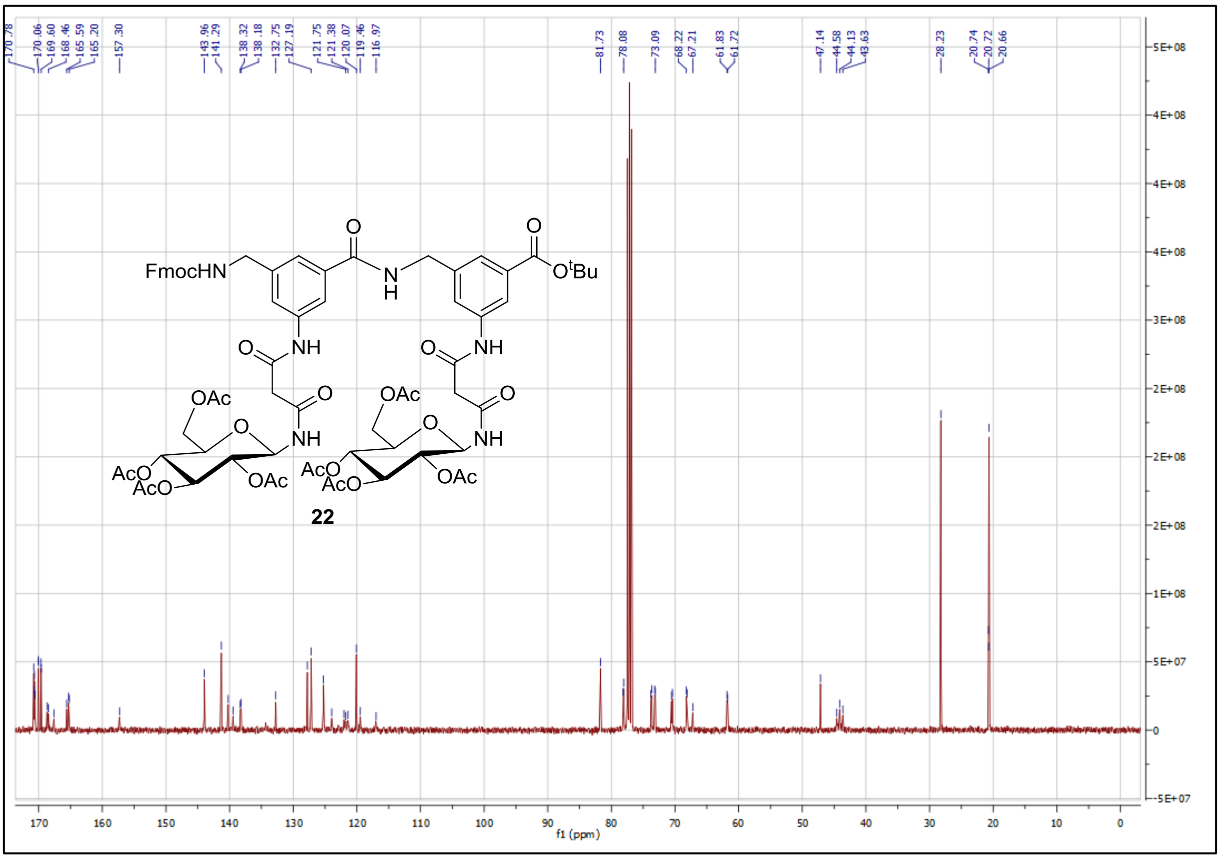
170.78
170.06
169.60
168.46
165.59
165.20
157.30
143.96
141.29
138.32
138.18
132.75
127.19
121.75
121.38
120.07
119.46
116.97
81.73
78.08
73.09
68.22
67.21
61.83
61.72
47.14
44.58
44.13
43.63
28.23
20.74
20.72
20.66
FmocHN
O
N
H
O
O^{t}Bu
NH
O
NH
O
O
O
OAc
O
NH
AcO
AcO
OAc
OAc
O
NH
AcO
AcO
OAc
22
f1 (ppm)

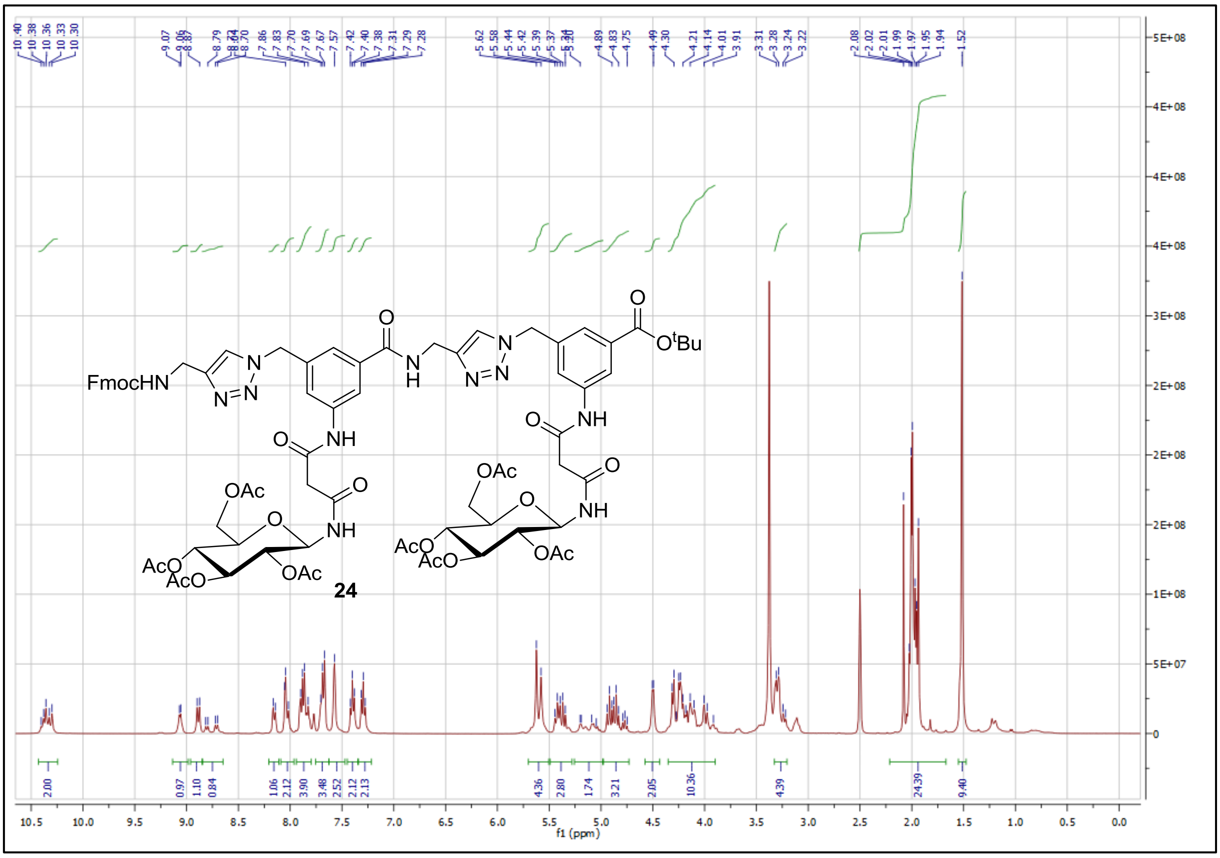

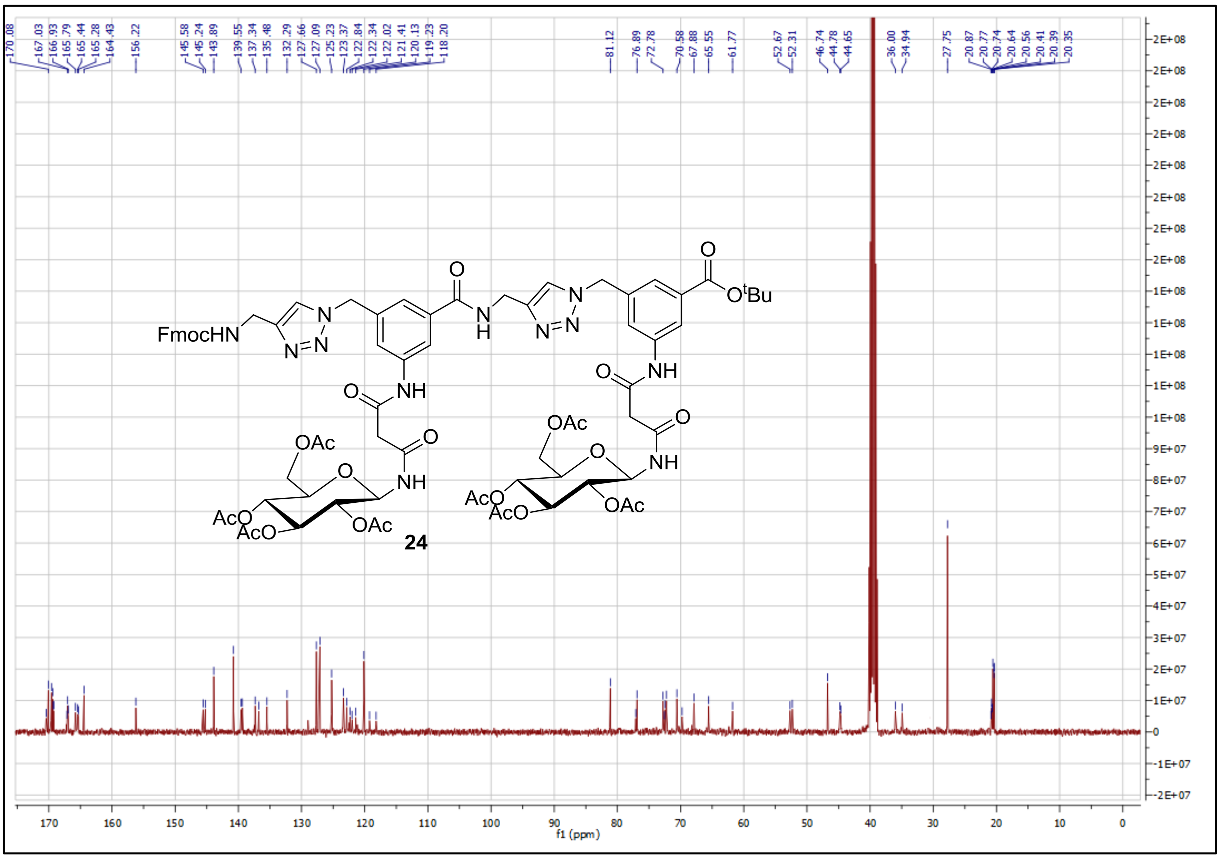

Supplement: File 2 — NMR Spectra. [file Beilstein_J_Org_Chem-10-2453-s002.pdf]
